# Divergent evolution in the genomes of closely related lacertids, *Lacerta viridis* and *L. bilineata*, and implications for speciation

**DOI:** 10.1093/gigascience/giy160

**Published:** 2018-12-10

**Authors:** Sree Rohit Raj Kolora, Anne Weigert, Amin Saffari, Stephanie Kehr, Maria Beatriz Walter Costa, Cathrin Spröer, Henrike Indrischek, Manjusha Chintalapati, Konrad Lohse, Gero Doose, Jörg Overmann, Boyke Bunk, Christoph Bleidorn, Annegret Grimm-Seyfarth, Klaus Henle, Katja Nowick, Rui Faria, Peter F Stadler, Martin Schlegel

**Affiliations:** 1 German Centre for Integrative Biodiversity Research (iDiv) Halle-Jena-Leipzig, Deutscher Platz 5e, Leipzig, 04103, Germany; 2 Bioinformatics Group, Department of Computer Science, and Interdisciplinary Center for Bioinformatics, Universität Leipzig, Härtelstrasse 16-18, Leipzig, 04107, Germany; 3 Molecular Evolution and Systematics of Animals, Institute of Biology, University of Leipzig, Talstrasse 33, Leipzig, 04103, Germany; 4 Max Planck Institute for Evolutionary Anthropology, Deutscher Platz 6, Leipzig, 04103, Germany; 5 Human Biology Group, Institute for Zoology, Department of Biology, Chemistry and Pharmacy, Freie Universität Berlin, Königin-Luise-Straße 1–3, Berlin, D-14195, Germany; 6 Embrapa Agroenergia, Parque Estacaeo Biologica (PqEB), Asa Norte, Brasilia/DF, 70770-901, Brazil; 7 Department of Microbial Ecology and Diversity Research, Leibniz Institute DSMZ-German Collection of Microorganisms and Cell Cultures, Inhoffenstrasse 7B, Braunschweig, 38124, Germany; 8 Max Planck Institute of Molecular Cell Biology and Genetics, Pfotenhauerstrasse 108, Dresden, 01307, Germany; 9 Max Planck Institute for Physics of Complex Systems, Noethnitzerstrasse 38, 01187 Dresden, Germany; 10 Center for Systems Biology Dresden, Pfotenhauerstrasse 108, 01397 Dresden, Germany; 11 Institute of Evolutionary Biology, University of Edinburgh, King's Buildings, Charlotte Auerbach Road, Edinburgh, EH9 3FL, United Kingdom; 12 Department of Animal Evolution and Biodiversity, University of Göttingen, Untere Karspüle 2, Göttingen, 37073, Germany; 13 Museo Nacional de Ciencias Naturales, Spanish National Research Council (CSIC), Madrid, 28006, Spain; 14 Department of Conservation Biology, UFZ - Helmholtz Center for Environmental Research, Permoserstrasse 15, Leipzig, 04318, Germany; 15 Plant Ecology and Nature Conservation, University of Potsdam, Am Mühlenberg 3, Potsdam, 14476, Germany; 16 Department of Animal and Plant Sciences, Alfred Building, University of Sheffield, Western Bank, Sheffield, S10 2TN, United Kingdom; 17 Competence Center for Scalable Data Services and Solutions Dresden/Leipzig, Universität Leipzig, Augustusplatz 12, Leipzig, 04107, Germany; 18 Max-Planck-Institute for Mathematics in the Sciences, Inselstrasse 22, Leipzig, 04103, Germany; 19 Fraunhofer Institut Für Zelltherapie Und Immunologie, Perlickstrasse 1, Leipzig, 04103, Germany; 20 Department of Theoretical Chemistry, University of Vienna, Währinger strasse 17, Wien, 1090, Austria; 21 Center for non-Coding RNA in Technology and Health, University of Copenhagen, Gronnegardsvej 3, Frederiksberg C, 1870, Denmark; 22 Santa Fe Institute, 1399 Hyde Park Road, Santa Fe, New Mexico, 87501, USA

**Keywords:** sister species, PacBio and Illumina, *de novo* hybrid assembly, transcripts, noncoding RNA, zinc fingers, positive selection, UV response, inversions, gene flow

## Abstract

**Background:**

*Lacerta viridis* and *Lacerta bilineata* are sister species of European green lizards (eastern and western clades, respectively) that, until recently, were grouped together as the *L. viridis* complex. Genetic incompatibilities were observed between lacertid populations through crossing experiments, which led to the delineation of two separate species within the *L. viridis* complex. The population history of these sister species and processes driving divergence are unknown. We constructed the first high-quality *de novo* genome assemblies for both *L. viridis* and *L. bilineata* through Illumina and PacBio sequencing, with annotation support provided from transcriptome sequencing of several tissues. To estimate gene flow between the two species and identify factors involved in reproductive isolation, we studied their evolutionary history, identified genomic rearrangements, detected signatures of selection on non-coding RNA, and on protein-coding genes.

**Findings:**

Here we show that gene flow was primarily unidirectional from *L. bilineata* to *L. viridis* after their split at least 1.15 million years ago. We detected positive selection of the non-coding repertoire; mutations in transcription factors; accumulation of divergence through inversions; selection on genes involved in neural development, reproduction, and behavior, as well as in ultraviolet-response, possibly driven by sexual selection, whose contribution to reproductive isolation between these lacertid species needs to be further evaluated.

**Conclusion:**

The combination of short and long sequence reads resulted in one of the most complete lizard genome assemblies. The characterization of a diverse array of genomic features provided valuable insights into the demographic history of divergence among European green lizards, as well as key species differences, some of which are candidates that could have played a role in speciation. In addition, our study generated valuable genomic resources that can be used to address conservation-related issues in lacertids.

## Introduction

Understanding what species are and the processes driving their emergence have been two central issues in biology [1]. During the last century, genes involved in reproductive isolation were mainly identified in model organisms, such as *Drosophila* [2]. These studies aiming at the so-called speciation genes revealed at least three general patterns: (i) many genes involved in post-zygotic incompatibilities show accelerated evolution [2]; (ii) incompatibilities often involve a disproportionate number of genes located on sex chromosomes [3, 4]; and (iii) mis-expression is often observed in hybrids, suggesting that gene regulation is an important component of speciation [5–7]. However, the identification of incompatibilities using laborious lab crosses was only possible for model organisms, and thus the identification of loci involved in reproductive isolation [8] in natural populations remained largely unknown.

The advent of high-throughput sequencing together with the development of novel approaches for whole genome analyses opened new research avenues to study the origin of species, including non-model organisms [9]. It has been shown that genes involved in adaptation and speciation are often found in regions of low recombination, such as genomic rearrangements, suggesting that they play an important role in species diversification [10]. Several *in silico* tools have been developed to detect structural variation with high precision using genomic data [11–13], thus enabling us to test evolutionary hypotheses such as the role of genomic rearrangements in speciation over a wider taxonomic range [14].

The assessment of divergence in regulatory elements and transcription factors between species further adds to a more complete understanding of the link between genotypes and phenotypes. In this respect, transcriptome sequencing offers an unprecedented resolution to investigate the general importance of divergence in gene regulation in speciation. In particular, zinc-finger genes, especially *Krüppel*-type zinc fingers (KZNFs), a family of transcription factors, were pinpointed as strong candidates to play a role in the speciation of other vertebrates [6]. In addition, various epigenetic mechanisms between species mediated by non-coding RNA (ncRNA) can also contribute to speciation [15–18].

Nevertheless, our understanding of how speciation unfolds, as well as the mechanisms involved, will remain limited without the knowledge of the demographic history between diverging taxa [8]. Model-based methods are now available to infer the demographic history of recently diverged taxa based on genome data from a few individuals of each species [19]. Thus, patterns of gene flow and population size changes during divergence can now be inferred without extensive sampling [20].

In summary, the identification of differences in genomic features between closely related species and their demographic history can now be assessed in a cost-effective manner. The resulting information is likely to provide insights about the main candidates playing a role in diversification, upon which more specific hypotheses concerning the mechanisms of divergence can be tested.

Lizards provide an excellent model for studying speciation due to the existing knowledge of their long-term demographics and adaptive morphologies, in addition to the ease of sample collection and experimental manipulations [21]. Lizards of the genus *Anolis*, in particular, have been studied in detail, as their distribution on islands coupled with repeated adaptive radiations offer a perfect framework for evolutionary ecology studies [22]. Not surprisingly, the first sequenced squamate genome was an anole lizard [23]. Comparative genomic analysis of *Anolis carolinensis* (anole lizard) with the genomes of birds and mammals was pivotal in identifying accelerated evolution of egg proteins associated with amniote evolution [23]. Further sauropsid genomes (birds and reptiles) were sequenced in recent years, now covering a broader taxonomic range of Squamata, Archosauria, and Chelonia [24–31]. For instance, the study of *Gekko japonicus* (gecko lizard) contributed to the understanding of evolution and adaptation of tail regeneration, clinging, nocturnal vision, and diversification of the olfactory system [26]. In addition, the genomes of *Pogona vitticeps* (bearded dragon lizard) and *Shinisaurus crocodilurus* (Chinese crocodile lizard) have recently been characterized [24, 27]. However, comparative genome analyses of closely related lizard species pairs have been limited to anoles, where adaptive evolution of genes related to brain development and behavior was recently reported [32].

The family Lacertidae (Sauropsida, Squamata) has been well covered in terms of phylogeographic studies, providing important information about the likely timing and geographic context of speciation [21]. Within this family, the *Lacerta viridis* complex shows an intricate evolutionary history with secondary contact zones [33, 34]. Here, we focus on the divergence between the western clade formally described as *Lacerta bilineata* (NCBI:txid95620) and the eastern clade of *L. viridis* (NCBI:txid65476) (corresponding to lineage B and lineage V, respectively, of Marzahn et al.) that currently occupy disjointed regions in Europe [34].

Adult individuals from the two taxa are very similar: throat coloration of hatchlings and early juveniles is the only described diagnostic trait so far [35]. Gene flow between these two species was previously hypothesized in studies of allozyme variation [36, 37]. However, recent analyses based on mtDNA and one nuclear marker (fibint7) have cast doubt on the taxonomic classification of the individuals analyzed in those studies and did not provide conclusive evidence either for or against gene flow between *L. viridis* and *L. bilineata* [34].

Hybrids between different main lineages within the *L. viridis* complex (northern Italy and Hungary) exhibit reduced fitness under laboratory conditions [38]. This suggests that at least partial reproductive isolation between *L. viridis* and *L. bilineata* can exist in the wild due to potential genomic Bateson-Dobzhansky-Muller incompatibilities (BDMIs). Previous models have suggested that after a secondary contact, BDMIs can be maintained and further accumulate within genome rearrangements [39, 40], thus avoiding species fusion [41]. High karyotypic variability has been observed in reptiles [42], also within the *L. viridis* complex [43], raising the prospect that genomic rearrangements could also be involved in their diversification [44]. Finally, lizard-specific KZNF genes have recently been predicted [45], making our focal pair of taxa an excellent case study of evolution in this class of genes and their role in speciation via changes in gene regulatory networks. Overall, the *L. viridis* complex comprises a very interesting system where different genomic components can be studied to elucidate the demographic history and possible processes involved in speciation.

Here, we combine short Illumina and long Pacific Biosciences (PacBio) read sequencing approaches to construct high-quality *de novo* genomes for both *L. bilineata* and *L. viridis*, with annotation support from transcriptomic data. We investigated the demographic history of divergence between the two lacertid taxa and performed a broad comparison of key genomic features providing important insights about their divergence that can be tested in future studies aiming to identify the mechanisms ultimately leading to speciation between this closely related species pair.

## Results

### Genomes of *L. viridis* and *L. bilineata*

We employed a hybrid strategy of combining Illumina and PacBio sequencing data to produce separate genome assemblies for the two lacertid species. Genome sequencing coverages of 34x Illumina and 14x PacBio for *L. viridis* and 37x Illumina and 11x PacBio for *L. bilineata* aided in the construction of high-quality genome assemblies (Supplement SI-1; Supplementary Figs. S2, S3). The genome assembly sizes were 1.44 Gbp and 1.42 Gbp for *L. viridis* and *L. bilineata*, respectively. The assembled lacertid genomes achieved better contiguity than the high-coverage illumina-only contigs of *G. japonicus* but lower than the chromosome-level assembly of *A. carolinensis* (368 kbp and 663 kbp for *L. bilineata* and *L. viridis*, respectively, vs 150 Mbp in *A. carolinensis*) (Supplementary Table S1). The Benchmarking Universal Single-Copy Orthologs completeness in terms of single-copy ortholog (SCO) genes with vertebrate core gene set were 96% and 94%, respectively, higher than in the available lizard genomes. Since the genome of *L. viridis* had better contiguity than *L. bilineata* (higher N50 and fewer contigs), *L. viridis* was used as the reference to predict genomic variants, structural variants (SVs) and single-nucleotide polymorphisms between the two taxa.

The *L. viridis* genome consisted of a higher number of large segmental duplications (>5 kbp) compared to *L. bilineata* (Supplementary Fig. S4). However, no significant differences were observed in segmental duplications (>1 kbp) between the two lacertid genomes (F-test: *P* = 0.35 and Wilcoxon test: *P* = 0.55). Hence, the occurrence of fewer large segmental duplications in *L. bilineata* could be the result of its more fragmented genome assembly. Synteny information was used to create unordered contig clusters (minimum size of 1 Mbp covering one-third of the *L. viridis* genome), which roughly represent positioning on the same chromosome (Supplementary File S2). The median synonymous substitution rate (Ks) and non-synonymous substitution rate (Ka) based on 7,030 SCOs between the two lacertid species were 0.021 and 0.016, respectively. A mutation rate of 1 × 10^−9^ substitutions per site per generation was estimated from the four-fold degenerate sites. This mutation rate observed in the ancestral lacertid lineage is similar to the ancestral bird lineage (1.15–1.23 × 10^−9^ per site per generation) [46, 47].

The identical structures of the HOX-cluster between the lacertid species and *A. carolinensis* confirmed the high genomic assembly quality since the HOX-clusters are highly conserved (Supplement SI-1). The number of chromosomes and the sex-determination system are different between *A. carolinensis* (2n = 36, 12 macro- and 24 microchromosomes; XY) and lacertid lizards (2n = 38, 36 macro- and 2 microchromosomes; ZW) [43, 48]. However, genomic contigs of both lacertid species were syntenic without breaks or inter-chromosomal transpositions to the macro-chromosomes of *A. carolinensis* (Supplementary Fig. S2), even though the lacertids and anoles split more than 150 million years ago (Mya) [49]. The only exception to this was a *L. viridis* contig that splits into two macro-chromosomes of the *A. carolinensis* genome. This particular contig of *L. viridis* was syntenic to five separate contigs in the *L. bilineata* assembly, confirming a higher fragmentation in genome assembly of the latter.

The assembled transcripts were crucial for gene annotations since the *ab initio* methods predicted more fragmented proteins and coding sequences (CDS) (38,000–55,000) when compared to the final gene models (22,100–22,500) (Supplementary Table S2). A majority of the longest *de novo* assembled transcript isoforms were from the ovarian tissue followed by the brain. Since the sequencing throughput was highest for the liver tissue in both species, this finding was not likely the result of sequencing artifacts. We identified 22,156 genes in *L. viridis* and 22,491 genes in *L. bilineata* supported by *de novo* assembled transcripts (Supplement SI-2; Supplementary Table S2). The larger number of genes in *L. bilineata* was due to the fragmentation of a few genes onto multiple contigs, which can be resolved in the future with scaffolding information. Compared to *A. carolinensis*, we observed an over-representation of genes involved in transfer RNA (tRNA) aminoacylation (Panther release 20170413, fold-enrichment = 2.13–2.25, *P*< 0.03) and tRNA metabolic process (Panther release 20170413, fold-enrichment = 1.84–1.89, *P*< 0.003) in both lacertids, indicating an expansion of tRNA-processing genes before their split. Putative Z-chromosome linked contigs consisted of few non-coding elements (7–11 microRNAs, 1 snoRNA, 2–3 snRNAs, and 46–53 functional tRNAs) (Supplement SI-3). The total length of the contigs assigned to the Z-chromosome in lacertids was larger (13.5–15.6 Mbp) than the Z-chromosomes of *P. vitticeps* (8 Mbp), but the number of identified genes were similar (205–221 and 219, respectively) [50].

The number of predicted members of the different non-coding RNA classes was similar in *L. viridis* and *L. bilineata* (Supplementary Table S3). Compared to other selected tetrapod species, there was an increase in the number of tRNAs (both functional and pseudo tRNAs) in the two lacertid species (Supplementary Figs. S5, S6). However, the numbers of tRNAs and pseudo tRNAs are known to vary significantly in eukaryotes [51]. We found an over-representation of tRNA-processing genes supported by the expansion of tRNA elements in both lacertid species maintained through deletion-duplication events. The microRNAs and snoRNAs in the lacertids exhibited losses compared to *A. carolinensis* (Supplementary Figs. S7, S8). Even though the numbers of snoRNAs and miRNAs were almost identical, the members in each ncRNA class diversified between the two sister species. Repeat content also differed between *L. viridis* and *L. bilineata*, with the latter exhibiting a gain of long-terminal repeat elements (Supplementary Tables S4, S5).

### Demographic history of divergence

Across all sites, mean heterozygosity was slightly lower in *L. bilineata* than in *L. viridis* (π = 0.0022 and 0.0029, respectively). d_xy_ was around 0.0123, pairwise F_st_ between *L. viridis* and *L. bilineata* was 0.688.

We inferred past divergence and gene flow between the two lacertid species using a composite likelihood (CL) method based on the site frequency spectrum of short sequence blocks, i.e., blockwise site frequency spectrum (bSFS) [20, 52]. Since the likelihood calculation assumes no recombination within blocks and an infinite sites mutation model, we partitioned the genome into short (i.e., 200 bases) blocks. Our dataset consisted of 5,654,020 blocks, of which 46,825 were filtered out (0.83%) since they contained both fixed differences and shared heterozygous sites, thus violating the 4-gametes criterion under the assumption of no recombination within blocks.

In total, we have 2,785 distinct mutational configurations, i.e., the counts of the four entries of the folded joint site frequency spectrum (heterozygous sites unique to *L. viridis* and *L. bilineata*; heterozygous sites shared by both lacertids; fixed differences) in each block. A total of 1,965 bSFS configurations appeared more than once in the data (Supplementary Information 3).

**Figure 1:**
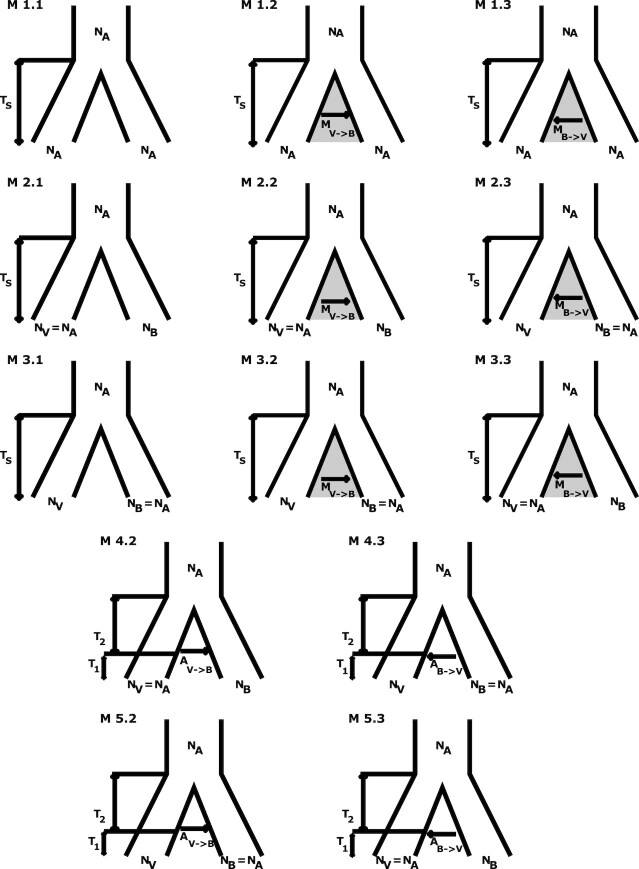
Thirteen different demographic scenarios were fitted. The models M1.1, M2.1, and M3.1 are strict divergence without gene flow; M1.2, M2.2, and M3.2 allow for post-divergence gene flow from *L. viridis* to *L. bilineata*; M1.3, M2.3, and M3.3 assume gene flow in the reverse direction, i.e., from *L. bilineata* to *L. viridis*. The models M4.2 and M5.2 allow for discrete admixture from *L. viridis* to *L. bilineata* and models M4.3 and M5.3 assume the admixture in the reverse direction (from *L. bilineata* to *L. viridis*). The effective population size is either assumed to be identical between both species and their ancestor (class M1.*) or one of the species has a different effective population size compared to the other species and ancestor (classes M2.*-5.*).

We compared the Akaike information criterion (AIC) scores of 13 different demographic scenarios (Fig. 1; Table 1) given the pattern of bSFS between the two lacertid species (Supplementary file 3). However, this composite likelihood computation does not account for the correlation between adjacent blocks due to the physical linkage. To correct for this, we assumed that every 1,000th block is effectively unlinked (Supplementary Information 3, Section LD), i.e., statistically independent, and corrected lnCL scores by a factor of 1/1000.

**Table 1:** The ΔAIC of the best model (i.e., IM 2 B(x)−>V) compared to the other scenarios .

Model ID	Model type	ΔAIC
M5.3	ADM 2B(x)−>V	−47.2
M3.2	ADM 2V (x)−>B	−141
M4.3	ADM 2B−>V (x)	−47.2
M3.2	ADM 2V −>B(x)	−141
**M3.3**	**IM 2B(x)−>V**	**0**
M3.2	IM 2V(x)−>B	−77.9
M3.1	DIV 2 b	−1,380
M2.3	IM 2B−>V (x)	−32.9
M2.2	IM 2V −>B(x)	−86.5
M2.1	DIV 2	−1,140
M1.3	IM 1B−>V	−487
M1.2	IM 1V −>B	−128
M1.1	DIV 1	−1,380

To account for the effect of physical linkage between blocks, we adjusted the AIC values of each model by only sampling every 1,000 blocks. The best model is highlighted in bold. ADM: isolation with discreet admixture; IM: isolation with migration; DIV: strict divergence without gene flow.

The best of the 13 models (M3.3) supports isolation between the two lacertid species with unidirectional gene flow from *L. bilineata* to *L. viridis* and fits significantly better than simpler scenarios such as divergence without gene flow (or just a single N_e_ parameter) or admixture (Table 1). This model (M3.3) also suggests a smaller effective population size of *L. bilineata* (Ne = 37,890) compared to its ancestor and *L. viridis* (Ne = 95,400) (Supplement SI-4; Supplementary Table S6) and a migration rate of M = 0.288 migrants per generation from *L. bilineata* to *L. viridis* (Supplementary Table S7).

Assuming a generation time of 3.5 years and a mutation rate of 1.77 × 10^−8^ (based on cyt-b gene) or 1 × 10^−9^ (based on the four-fold degenerate sites), our estimate of the split between *L. viridis* and *L. bilineata* corresponds to 1.15 Mya and 20.37 Mya, respectively (Supplementary Table S8).

### Detection of genomic rearrangements

**Figure 2:**
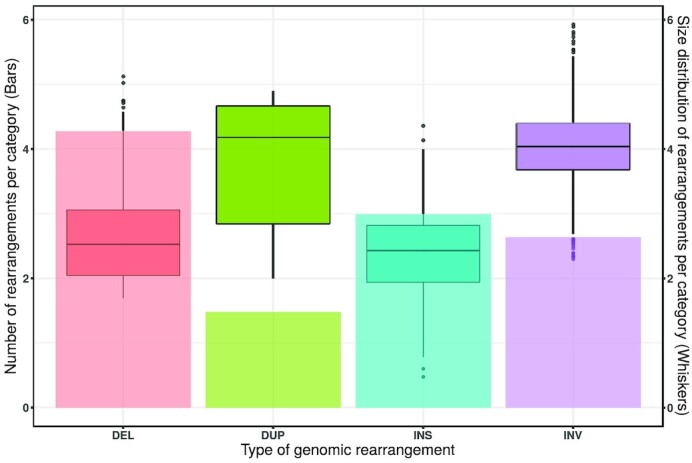
Total counts and length ranges (in bp) of genomic rearrangements of SVs between *L. viridis* and *L. bilineata*. The counts are represented by bars and length ranges by whiskers (y-axis is log10-scaled). The rearrangements plotted are categorized into deletions (DEL), duplications (DUP), insertions (INS), and inversions (INV).

We detected 20,160 genomic rearrangements or SVs longer than 50 bp between the two lacertids (Fig. 2; Supplementary Table S9) covering 39.4 Mbp of the *L. viridis* genome (2.7% of the genome). Compared to *L. bilineata*, 10.8 Mbp (0.7%) of the *L. viridis* genome was covered with large rearrangements affecting genes (covering the entire length of more than one gene). These regions were enriched for RNA-directed DNA polymerase activity (22.46 fold-enrichment, *P* = 5.11e-03).

Insertions-deletions (indels) are the most frequent genomic rearrangements mainly affecting introns, repeat elements, and pseudo-tRNAs (Supplement SI-5; Supplementary Table S10). This is similar to the observations made with respect to SVs in humans and pigs [12, 53]. Most SVs overlapping exons cover entire exons and do not result in frame shift mutations, with the exception of EXD2 and HERC2, suggesting that their functions can be complemented by other genes (Supplement SI-6).

### Structural selection of ncRNAs

MicroRNAs (miRNA) were the most structurally conserved family of ncRNAs followed by small nucleolar RNAs (snoRNA) (Supplementary Fig. S9). The four types of ncRNAs and the number of groups in each category are shown in Supplementary Table S11 (Supplement SI-7). High levels of diversity were observed in tRNAs, especially in pseudo-tRNA, which was further supported by high copy numbers of tRNAs with a low conservation among tRNA orthologs between the two lacertid species.

All ncRNAs with low structural diversity across orthologs were computationally tested for sites with positive selection in either species. The positively selected snoRNA families belong to the H/ACA box class, which can introduce changes in post-translational mechanisms and pseudouridylation between the two species [54]. SNORD61 (small nucleolar RNA, C/D Box 61) (Supplementary Fig. S10a) was inferred to have evolved under positive selection in *L. bilineata*. The human ortholog of SNORD61 occurs in the intron of a RBMX (RNA binding motif protein, X-linked gene), known to be involved in the dosage compensation and cohesion regulation of sister chromatids [55]. Two microRNAs showed signs of positive selection in *L. viridis*: MIR6516 (mir-6516-3p) (Supplementary Fig. S10b), associated with urea synthesis in pigs [56], and MIR27 (mir-27a and mir27-d) (Supplementary Fig. S10c), known to play a role in regeneration and osteoblast differentiation in mice [57, 58]. However, mir-27d was absent in *L. bilineata*, so the structural divergence in the mir-27 family between the two lacertid species can be due to the presence of an additional MIR27 sequence in *L. viridis*. Two long intergenic non-coding (lincRNA) orthologs (LiNC66 and LiNC29) overlapping genomic regions conserved across tetrapods were structurally divergent between the two species, as indicated by high selection scores and stable secondary structures (Supplementary Fig. S10d-e).

### Varying selection pressures in protein-coding genes

The visual opsins are pivotal for adaptation to diurnal habitats in Squamata [24, 59]. For instance, the nocturnal *G. japonicus* lost two of the five functional opsin paralogs compared to diurnal anoles [26]. All five paralogs of visual opsins in *A. carolinensis* (22 transcripts from ENSEMBL) were also present in *L. viridis* and *L. bilineata* (20 transcript sequences), indicating conservation of genes for diurnal vision. We observed high conservation of SWS1 (opsins related to UV vision), described to be involved in sexual selection [60, 61], and of the pigmentation protein MC1R, previously associated with adaptive coloration in sand lizards [62] (Supplement SI-8).

Genes involved in neuronal activity, behavior, auditory perception, and female reproductive system development were conserved in the lacertid ancestor, i.e., before the split between the two species (compared to five other vertebrates in the background). Genes with different selective constraints between the two species (i.e., differently influenced by purifying selection after their split) were related to brain and neural development, embryo and cartilage development, along with behavioral responses (Supplementary Table S12).

The test for positive selection in either of the two species was performed with the branch-site model of codeml (model M2) using a subset of other lizards as background branches. The number of genes with positively selected sites (PSS) in different foreground branches (*L. viridis*, *L. bilineata*, or the ancestor of *L. viridis* and *L. bilineata*) are shown in Supplementary Table S13 (Supplement SI-9). The predicted ontologies of genes with PSS in either of the two species indicate variation in growth and developmental processes, behavioral responses (temperature and pH), and transcriptional regulation (Supplementary Table S14). One of the genes with PSS in *L. bilineata* (STAR7) is located on the Z-chromosome. We identified two transcription factor genes, UBIP1 and RPA2, involved in gene silencing and reproductive functions [63, 64], with adaptive differences between the two species. Three genes with PSS overlapped inverted regions; GPR155 gene with PSS in *L. bilineata*, both TDRD3 and UGPA with PSS in *L. viridis*. GPR155 is involved in cognitive functions and expressed in mice forebrain [65], while TDRD3 is directly associated with oocyte formation and X-linked developmental disorders [66, 67]. Three genes, NASP, PDL11, and RTKN, were positively selected in the ancestor of the lacertid branch compared to background branches that include more distant classes such as mammals and birds (Supplement SI-9, Supplementary Table S15).

The prostacyclin synthase (PTGIS) involved in regeneration through prostaglandin synthesis is positively selected in *A. carolinensis* and *G. japonicus* [26]. This gene evolved under positive selection in the lacertid ancestor with *A. carolinensis* and *G. japonicus* as the background, hinting at evolutionary changes in regenerative mechanisms among lacertid lizards.

### Diversification of UV-responsive genes

We identified three paralogs of the hyaluronidases (HYAL1, HYAL2, and HYAL4) in both the lacertid genomes. Two genes (STIK1 and HYAL2) coding for proteins in the extra-cellular matrix of the skin reacting to UV-B light (GO:0071493) [68] were positively selected in the ancestral branch of the two species, while the HYAL1 paralog was positively selected in the *L. viridis* branch (Supplement SI-8). Arylsulfatase gene (ARSB), which is involved in the chondroitin sulfate biosynthesis pathway along with HYAL, was also positively selected in *L. viridis*. Significant pathway enrichment of chondroitin sulfate biosynthesis was observed for PSGs in *L. viridis* (*P* = 2.6e-06, *q* = 1.3e-05).

### Divergence of Kruppel-type zinc-finger proteins

To investigate the role of *Krüppel*-associated box (KRAB)-ZNFs in reproductive isolation of the two lacertid species, we compared the DNA-binding domains of KZNF orthologs. From the 53 KZNF orthologs, 6 C2H2 zinc-finger proteins showed binding-specific differences between the two lacertid species (Supplement SI-10). While the longest transcripts of these six KZNFs were assembled from ovarian tissues (Supplementary Table S16), they were also expressed in all the other tissues analyzed (brain, heart, liver, and kidneys).

### Impact of rearrangements on sequence evolution

Deletions are the most frequent type of SVs in the genome and occurred on both positively selected genes and those with no signs of positive selection. Duplications and insertions only occurred in genes evolving without signs of positive selection, while deletions and inversions occurred in genes irrespective of their selective regime. The ratio between number of regions with rearrangements or SVs to those with no detected rearrangements was not significantly different between genes under positive selection and those with no signs of positive selection (Boschloo's exact test, two-sided; difference in proportion = 0.125, *P* = 0.06, *q* = 0.1). Since this can be due to abundant indels obscuring the association in other categories of SVs, we tested the association between each SV category with PSGs separately, applying independent Boschloo exact-tests (Supplementary Table S17). An association of PSGs within inversions when compared to other SV categories was observed, but this did not remain significant after multiple testing (*P* = 0.028, *q* = 0.06). We also observed a significant association of PSGs over genes with no signs of positive selection within inversions compared to both non-rearranged regions (*P* = 0.009, *q* = 0.03) and collinear regions (*P* = 0.006, *q* = 0.03). The inversions overlapping PSGs seem to reflect independent events, since the inversions are located on different contigs in the genome with size ranges between 70 kbp and 700 kbp.

## Discussion

We provide high quality assembled genomes of two closely related lacertid species, *L. viridis* and *L. bilineata*, investigate their history of divergence, and analyze the patterns of genomic variation between these species.

The assembly contiguity was highest with partial error correction of PacBio reads (without splitting at chimeric junctions) followed by hybrid assembly through DBG2OLC implementing removal of chimeric joins. This hybrid assembly strategy aided in generating high-quality contig-level genomes with moderate genome coverages (∼35X Illumina and ∼15X PacBio). Our lacertid genome assemblies showed higher completeness than the available lizard genomes (Supplementary Table S1).

The time of population divergence between *L. viridis* and *L. bilineata* was estimated as at least 1.15 Mya (per generation), whereas the previously estimated mitochondrial divergence time was 2.6–3.4 Mya [33, 69]. *Lacerta viridis* and *L. bilineata* show a high level of genome-wide differentiation (F_ST_ = 0.688). The best demographic model (M3.3) supported unidirectional gene flow from *L. bilineata* to *L. viridis* and higher effective population size for *L. viridis* than *L. bilineata*, consistent with the difference in genetic diversity between the two lacertid species.

Species-specific diversity within various ncRNA classes and adaptive differences in ncRNA orthologs capable of altering their secondary structures are two important factors contributing to evolutionary divergence, since varying ncRNA structures imply functional changes [17]. Copy number variation and differences in the content of miRNA families hint at variability in gene regulatory networks between the lacertid sister species. Species-specific splicing mechanisms can be attributed to the losses of snoRNA families (SNORA17 and SNORA20) in *L. bilineata* and structural changes in SNORD61, which is involved in dosage compensation in humans [70].

Positive selection of sites in NASP and PDLIM1 in the lacertid ancestral branch compared to distant background branches, including mammals and birds, may indicate disparate evolutionary changes in the ancestor of *L. viridis* and *L. bilineata* with regard to reproductive processes, i.e., spermatogenesis, fertilization, and embryo implantation [71–74]. In contrast, positive selection acting on coding sites in just one lacertid species after their split suggests adaptive differences that could play a role in the speciation process [75–77].

UV-reflectance of plumages in birds is an important trait involved in the sexual selection of morphologically similar sibling species of Passeriformes [78]. Sexual selection in *L. viridis* has also been linked to UV-response. Males with more UV-reflective patches on the skin are preferably selected by the females [79, 80]. We show that hyaluronidases, known to be differentially expressed on exposure to UV-B in the skin of mice [68, 81, 82], evolved rapidly in *L. viridis*. We speculate that differential cutaneous response to UV through changes in the chondroitin sulfate biosynthesis pathway could be driven by mating preferences, which could ultimately contribute to speciation. Further studies are needed to test this hypothesis.

KRAB-ZNFs or KZNFs are transcriptional regulators confined to tetrapod vertebrates [83] and are known to play a role in species-specific changes in gene regulatory network through binding domain differences between humans and chimps [6, 84–87]. The divergence of transcription factors, especially differences in DNA-binding regions of KZNFs as observed here, could eventually have contributed to some degree of reproductive isolation between the two species, which should be further tested. This receives further support from adaptive differences in the transcription factors (UBIP1 and RPA2) crucial for spermatogenesis [63, 64]. Varying levels of purifying selection in genes influencing forebrain development and behavior suggest different selective constraints between *L. viridis* and *L. bilineata*. The behavioral differences can be related to varying ecological habitats and environmental conditions [35] after the split of *L. viridis* and *L. bilineata*. Selective differences in genes related to behavior and brain development have been reported to be involved in the diversification of anoles [32].

Genomic regions harboring inversions are known to suppress recombination in heterokaryotypes facilitating speciation in the presence of gene flow [88] and in maintaining favorable combinations of locally adapted alleles at different loci [89]. Genomic inversions between the two lacertid species are significantly associated with positively selected genes (PSGs). Two of the three PSGs occurring within inversions play a role in cognitive and reproductive functions (GPR155 and TDRD3), suggesting that they could be involved in speciation. However, it is currently unknown if these inversions represent fixed differences between the two species, and the lengths of these inversions is at a lower scale (less than 1 Mbp) than those known to play a role in adaptation and speciation [10]. Future studies should try to address these issues, as well as the role of these genes in reproductive isolation between *L. viridis* and *L. bilineata*.

## Conclusions

We assembled the first high-quality genomes of two closely related species of European green lizards with a cost-effective strategy. Genes related to transcriptional regulation, behavior, and neural and reproductive development have diversified the most between the two lacertid species. Species-specific diversity of ncRNAs, adaptive evolution in regulatory elements, and transcription factors (including binding domain differences in KZNFs) indicate variation in gene regulatory networks between the two species. Adaptive evolution of genes responsible for differential cutaneous response to UV exposure, in particular, could be driven by mate choice and ultimately contribute to reproductive isolation. Altogether, we provide the first comprehensive study of the evolutionary history and genic, structural, and regulatory differences between the genomes of two closely related lacertid species. This comprises an important baseline for understanding the genomic regions and mechanisms involved in the speciation of European green lizards. In addition to a detailed analysis of the demographic history and evolutionary scenario of European green lizards, our study provides valuable resources that will help establish conservation guidelines for lacertids experiencing population declines due to habitat loss [90].

## Materials and Methods

### Sampling

Two adult females were sampled for this study, a *L. viridis* from Tokaj, northeastern Hungary (21.39775^o^E, 48.11363^o^N) (September 2013) and a *L. bilineata* from Mâlain, France (4^o^48’2.01”E, 47^o^21’16.27”N) (July 2014). There is no known morphological variation between the individuals of the two species (Supplementary Fig. S1). These represent two of the four main clades within the *L. viridis* complex [33, 34, 38, 91]. Animals were captured with permits of the issuing authorities (please refer to the Acknowledgements) and handled according to the guidelines of the Herpetological Animal Care and Use Committee of the American Society of Ichthyologists and Herpetologists. Tissues from the brain, heart, liver, kidney, and ovaries were dissected for tissue-specific transcriptome sequencing, and the remaining tissues were stored separately at –80°C.

### Whole-genome and transcriptome sequencing

Tail tissue from each sample was digested with proteinase K, and genomic DNA was extracted using a chloroform-based method [92]. The whole genome was sequenced using both short (Illumina) and long read (PacBio) sequencing techniques. Short-read libraries with insert sizes of 380 bp and 450 bp were prepared for each individual separately. The Illumina paired-end sequences were double-indexed using a multiplexing sequencing protocol [93, 94] on a HiSeq2500. SMRTbell template library was prepared according to the instructions from PacBio (Menlo Park, CA), following the Procedure and Checklist—Greater Than 10 kb Template Preparation. Briefly, for preparation of 15 kb libraries 10 µg (*L. bilineata*) and 20 µg (*L. viridis*) genomic DNA was damage-repaired twice, end-repaired, and ligated overnight to hairpin adapters applying components from the DNA/Polymerase Binding Kit P6 from PacBio. Reactions were carried out according to the manufacturer's instructions. BluePippin Size-Selection to greater than 15 kb was performed according to the manufacturer's instructions (Sage Science, Beverly, MA). Conditions for annealing of sequencing primers and binding of polymerase to purified SMRTbell template were assessed with the Calculator in RS Remote, PacBio. Long-read sequencing was carried out for both genomes with 20 SMRT Cells applying P6-C4 chemistry on a PacBio RS*-*II sequencer. Average PacBio read lengths of 14 kb and 12 kb were retrieved for *L. viridis* and *L. bilineata*, respectively.

RNA from each tissue was extracted using Trizol Reagent (Life Technologies, Carlsbad, CA) and purified with the RNeasy Mini Kit (Qiagen, Hilden, Germany). The mRNA was purified using the Dynabeads mRNA Purification Kit (Life Technologies). The purity and concentration of RNA and cDNA were checked using Nanodrop and Bioanalyzer 2100 (Agilent Technologies, CA), and fragments of length 200–250 bp were obtained using Ambion RNA fragmentation reagent. The first and second strands of cDNA were synthesized using random hexamer primers with SuperScript II reverse transcriptase (Life Technologies) and DNA Polymerase I with RNase H treatment (Life Technologies) respectively.

*Lacerta viridis* was sequenced on a single lane for a more accurate estimate of genome size and repeat content. In order to avoid lane and run biases, sequencing was distributed over three lanes with all genomes and transcriptomes.

### Non-coding RNA annotation and repeat analysis

Small ncRNAs were annotated on the genomic contigs by performing an infernal cmscan v1.1.1 (Infernal, RRID:SCR_011809) using the RFAM covariance models as input, and homologous ncRNA genes were filtered with a cutoff of 1e-06 [95, 96].

Additionally, ncRNA class-specific annotation methods were used for tRNAs, snoRNAs, and miRNAs. tRNAs were annotated using tRNAscan-SE v1.3.1 software (tRNAscan-SE, RRID:SCR_010835) with default parameters [97]. The Basic Local Alignment Search Tool (BLAST)-based snoStrip pipeline [98] was used to annotate snoRNAs. A comprehensive set of snoRNAs from vertebrates and aves were used as query set [99]. To detect miRNAs, the avian set of miRNAs were used as query sequences for a BLAST search in the lizard genomes. All resulting blast hits were filtered for the conservation of the seed region. The annotated snoRNAs and miRNAs in lacertids were validated by blast searches against this reference database, and mature miRNA sequence homologies were used. In the case of overlapping miRNA and snoRNA annotations, both were retained as it is known that snoRNAs can be processed into small derived RNAs from miRNA-like RNAs [100]. Putative lincRNAs were predicted based on the transcripts with no coding potential as assessed by Transdecoder of the Trinity v2.6.5 suite (Trinity, RRID:SCR_013048) [101] and mapping on their respective genome without chimeric paths. Furthermore, only the conserved lincRNAs with one-to-one orthologs between lacertids were retained.

For comparison, ncRNA families (except lincRNA) were also annotated in other selected sauropsid genomes. A reference database was created using sequenced and annotated genomes from reptiles, aves, and other vertebrates. The program ePoPe [102] was used to understand the evolution of snoRNAs and miRNAs in the lacertids through the construction of phylogenetic trees based on the gains and losses of ncRNA families.

The RepeatModeler v1.0.4 pipeline (RepeatModeler, RRID:SCR_015027) [103] was used to predict repeats in the genomes of lacertids. The predicted repeat families were used as initial libraries for *de novo* annotation of repeats using RepeatMasker v4.0.5 (RepeatMasker, RRID:SCR_012954) [104]. The evolution of these repeats was investigated using the repeat library available for tetrapod species (Database: 20140131).

### Population histories, gene flow, and coalescence

To assess the demographic history between *L. viridis* and *L. bilineata*, we used the blockwise composite likelihood approach. We analytically computed the probabilities of mutational configurations in blocks of fixed length using the bSFS framework [20].

**Figure 3:**
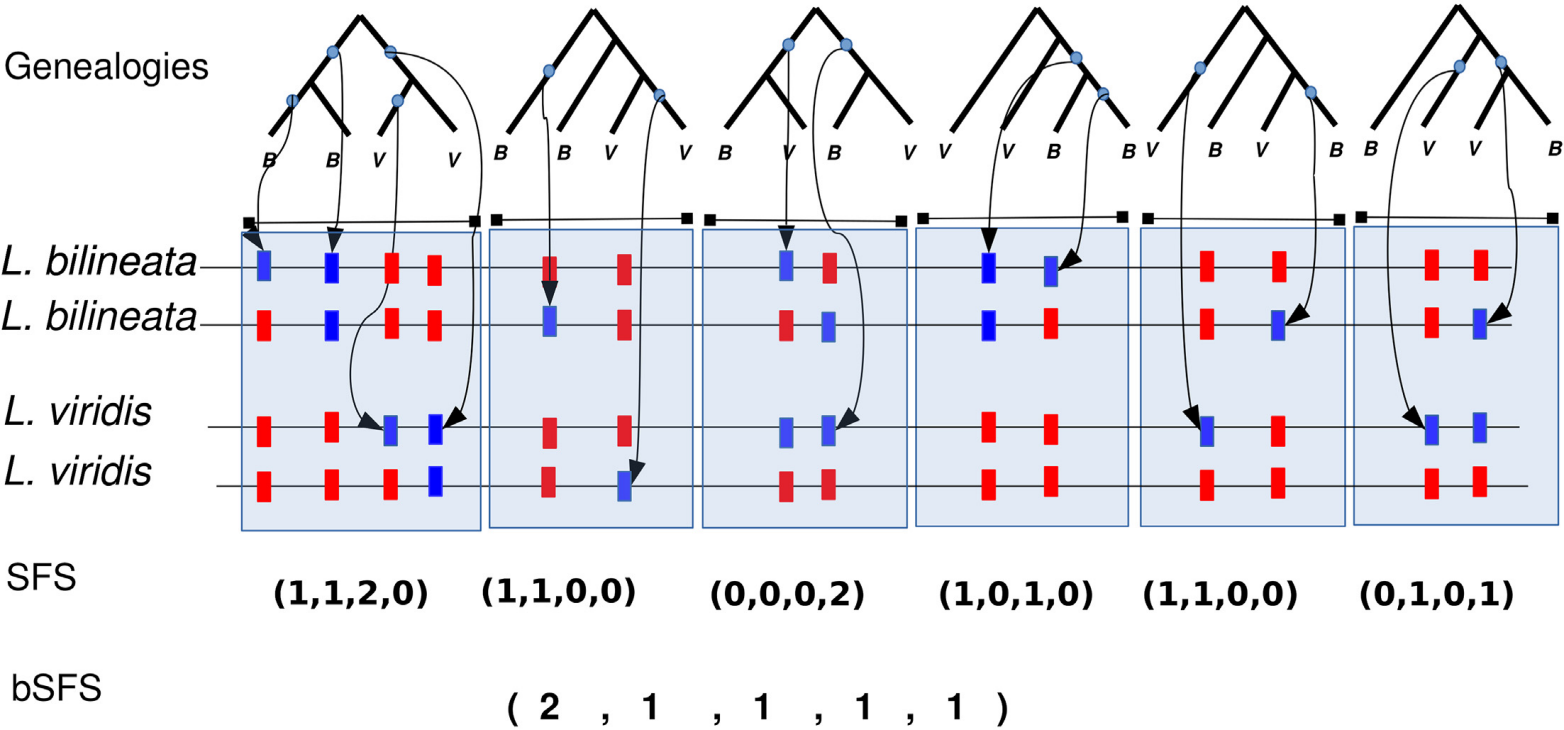
The folded blockwise site frequency spectrum (bSFS). The variation in alleles represented by different colors (the ancestral state showed in red). Given a single genealogy (a diploid genome from two populations can form six possible genealogies), each block contains four mutation types: (i) unique heterozygous sites in L*. bilineata*, (ii) unique heterozygous sites in *L. viridis*, (iii) shared heterozygous sites between *L. viridis* and *L. bilineata*, or (iv) homozygous sites that are different between *L. viridis* and *L. bilineata*, i.e., homozygous fixed differences. The bSFS (spectrum of SFS) has been calculated by counting the number of occurrences of each SFS.

We mapped the illumina reads from *L. viridis* and *L. bilineata* to the *L. viridis* reference genome with BWA MEM v0.7.12-r1039 software [105]. The homozygosity/heterozygosity of each site in both lacertids was predicted based on the reference genome with FreeBayes v9.9.13 (FreeBayes, RRID:SCR_010761) [106] with a minimum read support of five and minimum allele frequency of 0.2. The intergenic regions of the genome were chopped into blocks of length 200 bp; this resulted in 5,654,020 blocks in total. The number of four mutation types defined by the joint SFS (Fig. 3) were counted using Heffalump query ([107] commit 7773784). In total, 2,785 distinct mutational configurations were obtained, of which 1,965 appeared more than once. We then summarized the frequency of each polymorphism pattern across all blocks [108, 109]. This data summary is referred to as distribution of bSFS.

Blocks containing both fixed differences and shared heterozygous sites violate the four-gametes criterion, and 46,825 blocks (0.83%) were removed under the assumption of no recombination within blocks. To account for physical linkage between adjacent blocks, we assumed that every 1000th block is effectively unlinked, i.e., statistically independent, and corrected lnCL scores by a factor of 1/1000. We fitted 13 different demographic scenarios (Fig. 2) accounting for the presence or absence of gene flow, direction of gene flow, continuous or discrete migration, and changes in effective population sizes. Models were compared using the AIC of their composite log-likelihoods.

We estimated the generation length based on the mean age of the mothers of all offspring [110] given the age structure data by Elbing [111] and Saint Girons et al. [112] for three German populations of *L. viridis* and two French populations of *L. bilineata*, respectively. In captivity, females that breed for the first time lay on average 8.5 eggs, whereas older females lay 11.1 eggs [113]. Given this data, we estimated a mean generation length of 3.6 and 2.9 years for *L. viridis* and *L. bilineata*, respectively. We therefore assumed a generation time of about 3–4 years for both species. To scale our result in real time, we used a mutation rate of 1 × 10^−9^ per site per generation based on the four-fold degenerate sites of the single-copy gene orthologs between *L. viridis* and *L. bilineata* (Supplementary Methods SM-4). The lower limit of the mutation rate was assumed as 1.77 × 10^−8^ per site per generation (3.5 years as the generation time) based on the pairwise distance of 6.19% in the cytochrome b gene between *L. viridis* and *L. bilineata* [34]. This assumption is similar to the mutation rate of NADH-2 in *A. carolinensis* (1.3% mutations per million years) [114].

### Detection of genomic rearrangements from read-based pipelines and syntenic blocks

Genomic rearrangements between the lacertids were detected based on both read-based methods and syntenic blocks information. *Lacerta viridis* was used as the reference genome since the assembly was more contiguous for this species. Genomic reads from *L. bilineata* were used as the query, and the reads of *L. viridis* mapped against the reference were used as control.

#### Read-based pipelines

Genomic rearrangements were detected between lacertids using read mapping-based methods for Illumina paired-end reads and for PacBio-reads separately, followed by SV callers specifically developed to deal with short and long read sequences, respectively. In both approaches, reads of *L. bilineata* (query) and of *L. viridis* (control) were separately mapped against the same reference (*L. viridis*).

The alignment of Illumina reads was carried out with BWA MEM v0.7.12 [105], and rearrangements were detected with MetaSV v0.5.2 pipeline [115], which uses BREAKDANCER v1.1.2 (BREAKDANCER, RRID:SCR_001799) [116] to infer SVs using paired-end read information, CNVnator v0.3.1 (CNVnator, RRID:SCR_010821) [117] to predict copy-number variants from abnormal read-coverages and Pindel v0.2.4 (Pindel, RRID:SCR_000560) [118] to detect large SV-related breakpoint events. The insert size was estimated as 400±50 from 1 million observations based on the alignment of paired-end Illumina reads. A minimum support of five reads and mapping quality of 30 was set as the threshold to support SVs from BREAKDANCER. A bin size of 500 was used to run CNVnator, and only precise SV events were called. While for Pindel, only variants with minimum read support of five paired-reads were used. MetaSV pipeline was used to merge the SVs from these three different SV callers, and local *de novo* assemblies were constructed using the ABYSS assembler for insertions. In order to maintain a high level of sensitivity and specificity (>90%) in the detection of SVs, only the rearrangements called with a minimum support of eight uniquely mapped paired-end reads were used for further analyses [119].

The PacBio reads were aligned to the reference with NGMLR v0.2.1, and the alignment was fed to Sniffles v1.0.3 SV-caller [11] to call variants with a minimum support of seven reads (at least half of the PacBio genome coverage of 14X).

#### Syntenic blocks approach

In addition to read-based methods, rearrangements were also detected from the blocks of synteny obtained through the UCSC pipeline [120]. The alignments were converted to single-coverage genomes using single_cov2 of the MultiZ pipeline [121] to avoid spurious assignments. Strand changes within syntenic blocks were clustered as inversions (I) based on the orientation of the successive (I+1) and preceding (I-1) blocks. Regions with missing bases in the query alone were predicted to be deletions, while gaps in the reference genome alone were considered as insertions. Additionally, hierarchical alignment (HAL) format [122] of the single-coverage genomes was used to predict rearrangements with the halBranchMutations tool. This tool generates annotations for the location of rearrangements based on the branch of interest in the HAL file (between *L. viridis* and *L. bilineata* in our case). The events detected in both directions, i.e., *L. viridis* reference with *L. bilineata* as query and *L. bilineata* reference and *L. viridis* as query were retained. The length threshold was set to 50 bp, and the predicted rearrangements were filtered based on quality to reduce false positives (Supplement SM-7).

Segmental duplications in the two lacertid species were detected by self-aligning the two genomes separately with chained LASTZ [123] (step = 9, H = 3000, K = 5000). High identity matches (90% identity) within each genome of 1 kb or more were defined as segmental duplications.

### Structural selection in non-coding RNAs

The predicted ncRNAs (miRNA, snoRNA, tRNA, and lincRNA) in lacertids were tested for structural selection (selection of sites acting on secondary structure in either of the lacertids) with *Gekko japonicus* as outgroup. We used the Selection on the Secondary Structure (SSS) test [124], a statistical test that assigns selection scores for each given sequence based on the comparison between the structure of the given sequence and the structure of group consensus. It also provides a diversity value for the family that indicates its structural conservation. The diversity value (d-score) is the family's median base-pair distance to its consensus. The miRNAs, snoRNAs, and tRNAs were divided into sub-groups based on their families or their anti-codon sequences, and only those sub-groups with at least three sequences were tested. The groups that exhibited high structural diversity (median base pair distance to the consensus, d ≥10.0) were excluded from further analyses.

A ncRNA structural test to detect positively selected structures is only appropriate for structurally conserved groups. Low d-score values (d <10.0) were used to distinguish conservation chosen based on structural uniformity of the groups. This cutoff was based on the visual inspection of the secondary structures of families with d-scores of 1 to 20. Secondary structures of ncRNA sequences were predicted using RNAfold [125]. In a similar fashion, structures with selection scores of 0 to 30 were visually compared to the structure of their group consensus. High selection scores (s ≥10.0) were used to predict the positively selected sequences of small ncRNAs. Secondary structures with high selection scores were manually inspected to remove false positives. Specifically, the candidates with structures of low stability or those fundamentally dissimilar to the family consensus indicating loss of function were excluded.

The selection test was adapted for lincRNAs and performed only on the two lacertid species without any outgroup since lincRNA annotations of other closely related species were unavailable. Since the positive selection of secondary structure cannot be determined without outgroups, we instead detected divergence of lincRNA structure within the lacertids. Local conserved structure blocks were predicted for the orthologous lincRNA families, and these blocks were subjected to an adaptation of SSS test based on local structures. The structural selection for lincRNAs was assessed locally, since most base-pairings occur between nucleotides within a short distance [124, 126]. Local blocks of high structural diversity were excluded from further analysis. Since outgroups were not used for lincRNAs, a lower selection score threshold (s ≥4.0) was applied to detect divergent candidates that were visually inspected later to exclude false positives.

### Ortholog prediction and selection tests

In order to investigate the selection pressure in the lacertid branch (ancestor of *L. viridis* and *L. bilineata*) compared to other vertebrates, the CDS of five species, namely, anole lizard (*Anolis carolinensis*), chicken (*Gallus gallus*), frog (*Xenopus tropicalis*), spotted garfish (*Lepisosteus oculatus*), and human (*Homo sapiens*) were downloaded from the Ensembl database version 83 (Ensembl, RRID:SCR_002344) [127]. To keep the data consistent and avoid re-annotations, the CDS annotations were also extracted from the Ensembl database. The orthologs between the protein-coding sequences of the species were identified with ProteinOrtho V5 using the synteny option to reduce false orthologs assignments. The output was converted to run the POTION pipeline [128], which tests for selection acting on protein coding genes. Only the single-copy orthologs in each species were retained for each orthologous group.

The protein identity filtering in POTION was set to 30% in each orthologous group and sequence size limits to more than 10 times or less than 0.2 of the median size in the group. Only groups with at least four species were retained. The sequences in each orthologous group (after filtering paralogs) were aligned and gap trimmed; phylogenetic trees were constructed, and groups with recombinants were excluded from the selection tests. The intermediates files from the POTION pipeline were used to generate unrooted trees with lacertids (*L. viridis* and *L. bilineata*) in the foreground branches. The remaining species were used as the background to test for positive selection using the branch-site model of codeml within the PAML v4.8 package (PAML, RRID:SCR_014932) [129]. A likelihood ratio test based on χ^2^ distribution was used to detect genes with significant positive selection followed by multiple testing through the Benjamini–Hochberg procedure. Genes with *P <* 0.05 and *q <*0.05 were retained and referred to as being positively selected in the lacertid branch.

To detect adaptive evolution through positive selection within either lacertids, additional tests (PAML branch-site models) were performed with less distant outgroups using a set of five lizard species, namely, *L. viridis, L. bilineata*, *Anolis carolinensis*, *Gekko japonicus*, and *Pogona vitticeps*. The single-copy orthologs were identified with ProteinOrtho with a minimum protein identity of 70%, e-value of 1e-06, and minimum similarity of 0.99 for additional hits. The orthologous coding sequences from the five lizard species were aligned with MACSE while accounting for frame-shifts, and the stop codon at the end of the sequence was removed. Unrooted trees were generated with three different foreground branches: (i) lacertids (*L. viridis* and *L. bilineata*), (ii) *L. viridis* alone, and (iii) *L. bilineata* alone. The rest of the workflow for detection of recombinants, removal of gaps, and codeml tests was similar to the POTION pipeline followed by filtering for significant candidate genes (*P <* 0.05, *q <* 0.05). In order to avoid false predictions of PSS at the beginning or toward the end of alignments, where mismatches were allowed, the candidate genes predicted to contain PSS in either species were visually inspected.

## Availability of supporting data

The assembled genomes, their annotations, transcript data, variant calls (variant call formats), snapshots of the code, and other supporting datasets are available in Zenodo [130] and in the *GigaScience* GigaDB [131] repositories.

## Additional files

Additional file 1: This supplement contains methods SM1-SM11, information SI1-SI11, Figures S1–S10, Tables S1–S17 and References.

Additional file 2: The figure for the contig clusters in lacertids generated from synteny information between *L. viridis* and *L. bilineata*.

Additional file 3: *Mathematica* notebook containing the code used and other supporting information from the demography analysis of *L. viridis* and *L. bilineata*.

## Abbreviations

AIC: Akaike information criterion; BDMI: Bateson-Dobzhansky-Muller incompatibility; BLAST: Basic Local Alignment Search Tool; bSFS: blockwise site frequency spectrum; CDS: coding sequence; CL: composite likelihood; HAL: hierarchical alignment; HYAL: hyaluronidase; indels: insertions and deletions; KRAB: Krüppel-associated box; KZNF: Krüppel-type zinc finger; lincRNA: long intergenic non-coding RNA; miRNA: microRNA; Mya: Million years ago; NCBI: National Center for Biotechnology information; ncRNA: non-coding RNA; PacBio: Pacific Biosciences; PSG: positively selected gene; PSS: positively selected site; RBMX: RNA binding motif protein, X-linked gene; rRNA: ribosomal RNA; SCO: single-copy ortholog; SNORD61: small nucleolar RNA, C/D Box 61; SFS: site frequency spectrum; snoRNA: small nucleolar RNA; SSS: Selection on the Secondary Structure; SV: structural variants; tRNA: transfer RNA.

## Competing interests

The authors declare that they have no competing interests. The authors alone are responsible for the content and writing of the paper.

## Funding

This project was funded by the Deutsche Forschungsgemeinschaft (FZT 118, SFB 1052 and SPP 1738).

## Author contributions

K.H. and A.G. collected the samples; A.W. Illumina sequenced the genome and transcriptome; C.S., B.B., and J.O. were involved in the PacBio genome sequencing; S.R.R.K. performed the assembly and annotation; S.K. and S.R.R.K. annotated the non-coding RNA; M.B.W.C. analyzed selection in non-coding RNA; S.R.R.K. and H.I. performed the positive selection analysis; S.R.R.K. and M.C. analyzed structural variants; A.S. and K.L. analyzed the population histories; S.R.R.K., R.F., K.N., P.F.S., and M.S. wrote the initial draft of the manuscript; H.I., M.C., A.S., K.L., S.K., M.B. W.C., J.O., B.B., C.B., and K.H. edited the manuscript; R.F., K.N., K.H., P.F.S., and M.S. conceived the study.

## Supplementary Material

GIGA-D-18-00173_original_Submission.pdf

GIGA-D-18-00173_Revision_1.pdf

GIGA-D-18-00173_Revision_2.pdf

Response_to_Reviewer_Comments_Original_Submission.pdf

Response_to_Reviewer_Comments_Revision_1.pdf

Reviewer_1_Report_(Original_Submission) -- Ben Wielstra 6/20/2018 Reviewed

Reviewer_2_Report_(Original_Submission) -- Anthony Geneva 6/22/2018 Reviewed

Reviewer_2_Report_Revision_1 -- Anthony Geneva 10/28/2018 Reviewed

giy160_Supplemental_Files
