# Supplementary material for: Divergent evolution in the genomes of closely related lacertids, Lacerta viridis and L. bilineata, and implications for speciation
Source: Gigascience. 2018 Dec 10;8(2):giy160. doi: 10.1093/gigascience/giy160 (PMC6381762; doi:10.1093/gigascience/giy160)
Supplement: GIGA-D-18-00173_original_Submission.pdf [file giy160_giga-d-18-00173_original_submission.pdf]

GigaScience

Divergent evolution in the genomes of closely-related lacertids, Lacerta viridis and L. bilineata and implications for speciation

--Manuscript Draft--

**Manuscript Number:**	GIGA-D-18-00173
**Full Title:**	Divergent evolution in the genomes of closely-related lacertids, Lacerta viridis and L. bilineata and implications for speciation
**Article Type:**	Research
**Funding Information:**	Deutsche Forschungsgemeinschaft (FZT118) — Dr. Martin Schlegel
**Abstract:**	Background: Lacerta viridis and L. bilineata are sister species of European green lizards (eastern and western clades respectively), which until recently were grouped together as the L. viridis complex. Genetic incompatibilities were observed between lacertid populations through crossing experiments, which led to the delineation of two separate species within the L. viridis complex. The population histories of these sister species and processes driving divergence are unknown. We constructed high quality de novo genome assemblies for both L. viridis and L. bilineata through Illumina and PacBio sequencing, with annotation support provided from transcriptome sequencing of several tissues. To estimate gene flow between the two species and identify the intrinsic and extrinsic factors involved in reproductive isolation, we studied their evolutionary histories, identified cross-species genomic rearrangements, detected evolutionary pressures on non-coding RNA and genes undergoing varying selection pressures.<br>Findings: Here we show that the gene flow between the lacertid species was primarily unidirectional from L. viridis to L. bilineata since their split 2.7-3.05 Mya. Adaptive evolution of the non-coding repertoire, mutations in transcription factors, accumulation of divergence through inversions and selection on genes involved in neural development, reproduction and behavior have been critical for reduced reproductive success between lacertid species.<br>Conclusion: Divergent evolution between lacertid species is a result of adaptive evolution of non-coding elements, cognitive and reproductive genes. We propose that assortative mating in lacertids is influenced by diversification of genes responsible for cutaneous response on exposure to UV-B. Our results provide valuable insights into the demographic history and factors contributing to evolutionary divergence leading to speciation in European green lizards.
**Corresponding Author:**	Rohit Kolora<br><br>GERMANY
**Corresponding Author Secondary Information:**	
**Corresponding Author's Institution:**	
**Corresponding Author's Secondary Institution:**	
**First Author:**	Sree Rohit Rohit Kolora
**First Author Secondary Information:**	
**Order of Authors:**	Sree Rohit Rohit Kolora
	Anne Weigert
	Amin Saffari
	Stephanie Kehr
	Maria Beatriz Walter Costa
	Cathrin Spröer

Powered by Editorial Manager® and ProduXion Manager® from Aries Systems Corporation

	Henrike Indrischek
	Gero Doose
	Manjusha Chintalapati
	Konrad Lohse
	Jörg Overmann
	Boyke Bunk
	Christoph Bleidorn
	Klaus Henle
	Katja Nowick
	Rui Faria
	Peter F Stadler
	Martin Schlegel
**Order of Authors Secondary Information:**	
**Additional Information:**	
**Question**	**Response**
Are you submitting this manuscript to a special series or article collection?	No
**Experimental design and statistics**<br><br>Full details of the experimental design and statistical methods used should be given in the Methods section, as detailed in our Minimum Standards Reporting Checklist. Information essential to interpreting the data presented should be made available in the figure legends.<br><br>Have you included all the information requested in your manuscript?	Yes
**Resources**<br><br>A description of all resources used, including antibodies, cell lines, animals and software tools, with enough information to allow them to be uniquely identified, should be included in the Methods section. Authors are strongly encouraged to cite Research Resource Identifiers (RRIDs) for antibodies, model organisms and tools, where possible.<br><br>Have you included the information requested as detailed in our Minimum Standards Reporting Checklist?	Yes
**Availability of data and materials**	Yes

Powered by Editorial Manager® and ProduXion Manager® from Aries Systems Corporation

All datasets and code on which the conclusions of the paper rely must be either included in your submission or deposited in publicly available repositories (where available and ethically appropriate), referencing such data using a unique identifier in the references and in the "Availability of Data and Materials" section of your manuscript.<br><br>Have you have met the above requirement as detailed in our Minimum Standards Reporting Checklist?	

Manuscript
Click here to download Manuscript Lacertids_Manuscript-Gigascience.pdf

Divergent evolution in the genomes of closely-related lacertids, *Lacerta viridis* and *L. bilineata* and implications for speciation

Rohit Kolora, Anne Weigert[¥], Amin Saffari[¥], Stephanie Kehr, Maria Beatriz Walter Costa, Cathrin Spröer, Henrike Indrischek, Gero Doose, Manjusha Chintalapati, Konrad Lohse, Jörg Overmann, Boyke Bunk, Christoph Bleidorn, Klaus Henle, Katja Nowick, Rui Faria, Peter F Stadler, Martin Schlegel

[¥] These authors contributed equally to this work.

Abstract

Background: *Lacerta viridis* and *L. bilineata* are sister species of European green lizards (eastern and western clades respectively), which until recently were grouped together as the *L. viridis* complex. Genetic incompatibilities were observed between lacertid populations through crossing experiments, which led to the delineation of two separate species within the *L. viridis* complex. The population histories of these sister species and processes driving divergence are unknown. We constructed high quality *de novo* genome assemblies for both *L. viridis* and *L. bilineata* through Illumina and PacBio sequencing, with annotation support provided from transcriptome sequencing of several tissues. To estimate gene flow between the two species and identify the intrinsic and extrinsic factors involved in reproductive isolation, we studied their evolutionary histories, identified cross-species genomic rearrangements, detected evolutionary pressures on non-coding RNA and genes undergoing varying selection pressures.

Findings: Here we show that the gene flow between the lacertid species was primarily unidirectional from *L. viridis to L. bilineata* since their split 2.7-3.05 Mya. Adaptive evolution of the non-coding repertoire, mutations in transcription factors, accumulation of divergence

through inversions and selection on genes involved in neural development, reproduction and behavior have been critical for reduced reproductive success between lacertid species.

Conclusion: Divergent evolution between lacertid species is a result of adaptive evolution of non-coding elements, cognitive and reproductive genes. We propose that assortative mating in lacertids is influenced by diversification of genes responsible for cutaneous response on exposure to UV-B. Our results provide valuable insights into the demographic history and factors contributing to evolutionary divergence leading to speciation in European green lizards.

Introduction

Understanding what species are and the processes driving their emergence have been two central issues in biology [1]. Divergent evolution, which can eventually lead to speciation, is driven by various mechanisms, such as chromosomal rearrangements, polyploidy in plants, whole genome duplications followed by differential loss of genes and reduced hybrid viability or sterility through Bateson-Dobzhansky-Muller incompatibities (BDMIs) [2]. Both genetic drift and selection can lead to the emergence of reproductive barriers, in particular intrinsic BDMIs and, ultimately speciation.

During the last century, the genes involved in reproductive isolation were essentially identified through crosses in the lab [3]. Studies characterizing these so-called "speciation genes" in model organisms such as *Drosophila,* revealed several general patterns: i) genes involved in post-zygotic incompatibilities show signatures of accelerated evolution [3]; ii) incompatibilities often involve a disproportionate number of genes located on sex chromosomes [4, 5] and iii) mis-expression is often observed in hybrids, suggesting that gene regulation is an important component of speciation [6-8]. Genes involved in speciation can often be non-essential and reproductive isolation can be restricted to a few loci in the genome [9, 10].

The advent of high throughput sequencing has enabled comparative genomic analyses making it possible to identify genomic regions contributing to diversification. Additionally, their distribution across the genome provides crucial information to understand the genomic architecture of speciation [11]. For instance, genomic rearrangements can now be detected in model and non-model organisms using *in-silico* methods [12-14], allowing to test hypotheses about the role of genomic rearrangements in speciation [15]. Specifically theory suggests that rearranged regions of the genome can facilitate speciation and several empirical studies have shown that inversions show higher divergence and an enrichment for genetic incompatibilities between species compared to collinear regions [16-19]. Inversions are also known to facilitate speciation in the presence of gene flow in different ways: i) allow accumulation of genetic differences within rearrangements (including genes involved in reproductive isolation) despite gene flow [20, 21], ii) avoid species fusion after secondary contact [16] and iii) maintain favorable combinations of locally adapted alleles at different loci favoring spread of rearrangements [22].

Assessment of divergence in regulatory elements and transcription factors between species contributes to a more complete understanding of the link between genotypes and phenotypes. This opens the door to investigate the general importance of gene regulation in speciation, as well as more specific hypotheses such as the role of zinc-finger genes, especially *Krüppel*-type zinc fingers (KZNFs), a family of transcription factors in species diversification [7]. In addition to protein-coding regions in the genome, variations in non-coding RNAs (ncRNAs) act as precursors of speciation through differences in epigenetic mechanisms [23]. These functional differences are primarily attributed to species-specific diversity of ncRNA classes and structural evolution in conserved ncRNAs [24-26].

The demographic history of recently diverged taxa can now be inferred from genome-wide sequencing data. Different model-based methods are now available [27], including some that

make use of genomic data from a single individual from each species to test for migration as well as population size changes during divergence [28]. These methodological advances allow for a better interpretation of the genomic landscape of speciation and the evolutionary processes involved [29].

Lizards provide an excellent model for the study of speciation due to the existing knowledge on their long-term demographics and adaptive morphologies, in addition to the ease of sample collection and experimental manipulations [30]. Lizards of the genus *Anolis* have especially been studied in detail, as their distribution on islands coupled with repeated events of adaptive radiations offered a perfect framework for evolutionary ecology studies. Not surprisingly, the first sequenced squamate genome was an anole lizard [31]. Comparative genomic analysis of *Anolis carolinensis* (anole lizard) with the genomes of birds and mammals was pivotal in identifying accelerated evolution of egg proteins associated with amniote evolution [31]. Further sauropsid genomes (birds and reptiles) were sequenced in recent years, now covering a broader taxonomic range of Squamata, Archosauria and Chelonia [32-39]. The study of *Gekko japonicus* (gecko lizard) contributed to the understanding of evolution and adaptation of tail regeneration, clinging, nocturnal vision and diversification of the olfactory system [34]. In addition, the genomes of *Pogona vitticeps* (bearded dragon lizard) and *Shinisaurus crocodilurus* (Chinese crocodile lizard) have recently been characterized [32, 35]. However, comparative genome analyses of closely-related lizard species pairs have only been undertaken recently in anoles where adaptive evolution of genes related to brain development and behavior was reported [40].

In particular, the family Lacertidae (Sauropsida, Squamata) has been well covered in-terms of phylogeographic studies, providing important information about the likely timing and geographic context of speciation [30]. Within this family, the *Lacerta viridis* complex shows an intricate evolutionary history with secondary contact zones [41, 42]. Here, we focus on the

divergence between the western clade formally described as *L. bilineata* (corresponding to lineage B) and the eastern clade of *L. viridis* (lineage V) that currently occupy disjoint regions in Europe [42].

Adult individuals from the two taxa are very similar: throat coloration of hatchlings and early juveniles is the only described diagnostic trait so far [43]. Although ongoing gene flow between these two species was previously hypothesized in studies of allozyme variation [44, 45], recent analyses based on mtDNA and one nuclear marker (fibint7) have cast doubt on the taxonomic classification of the individuals analyzed in those studies [42]. Furthermore, the limited power of these two markers did not provide conclusive evidence either for or against gene flow between *L. viridis* and *L. bilineata*.

Hybrids between different main lineages within the *L. viridis* complex (northern Italy and Hungary) exhibit reduced fitness under laboratory conditions [46]. This suggests at least partial reproductive isolation between *L. viridis* and *L. bilineata* in the wild which can arise due to BDMIs. This raises the prospect that genomic rearrangements could be involved in the diversification of the *Lacerta viridis* complex [47, 48]. Lizard-specific KZNF genes have recently been predicted [49], making our focal pair of taxa an excellent case study of evolution in this class of genes and their role in speciation between via changes in gene regulatory networks.

Here we combine short Illumina and long PacBio read sequencing approaches to construct high quality *de novo* genomes for both *L. bilineata* and *L. viridis*, with annotation support from transcriptomic data. We investigated the demographic history of divergence between the two lacertid taxa, performed a broad comparison of genomic features contributing to species divergence and quantified selection in lacertid evolution.

Results

The genomes of *L. viridis* and *L. bilineata*

We employed a hybrid strategy of combining Illumina and PacBio sequencing data to produce separate genome assemblies for the two lacertid species. Genome sequencing coverages of 34x Illumina and 14x PacBio for *L. viridis*; 37x Illumina and 11x PacBio for *L. bilineata* aided in the construction of high quality genome assemblies (supplement SI-1; Figure S2, S3). The assembled lacertid genomes achieved better contiguity than the high coverage illumina-only contigs of *A. carolinensis* and *G. japonicus* (Table S1). The contig N50's of genomic assemblies of *L. viridis* and *L. bilineata* were 368kb and 663kb respectively, while the BUSCO (Benchmarking Universal Single-Copy Orthologs) completeness were 96% and 94% respectively which was higher than the available lizard genomes. Since the genome of *L. viridis* had better contiguity than *L. bilineata*, *L. viridis* was used as the reference to predict genomic variants (structural variants (SVs) and single nucleotide polymorphisms (SNPs) between the two taxa. There were no observable differences in segmental duplications between the lacertid genomes (Figure S4). The syntenic blocks between the genomes of *L. viridis* and *L. bilineata* had an identity of 98.2% (87% represented by pair-wise syntenies). Synteny information was used to create unordered contig clusters (min. size of 1 Mbp covering one-third of the *L. viridis* genome) which roughly represent positioning on the same chromosome (Suppl. File S2). The median synonymous substitution rate (Ks) and non-synonymous substitution rate (Ka) between the two lacertid species were 0.021 and 0.016 respectively. A divergence time of 2.5-2.9 million years was estimated between the two species based on 4d-sites.

The identical structures of the HOX-cluster between the lacertid species and *A. carolinensis* confirms the high genomic assembly quality since the HOX-clusters are highly conserved

(supplement SI-1). The number of chromosomes and the sex-determination system are different between anole lizards (2n=36, 12 macro- and 24 microchromosomes; XY) and lacertid lizards (2n=38; 36 macro- and 2 microchromosomes; ZW) [50, 51]. However, genomic contigs of both lacertid species were syntenic without breaks or inter-chromosomal transpositions to the macro-chromosomes of *A. carolinensis* (Figure S2), though the lacertids and anoles split more than 150 Mya. An exception to this was a single *L. viridis* contig which split over two macro-chromosomes of the *A. carolinensis* genome. This particular contig of *L. viridis* was syntenic to five separate contigs in *L. bilineata* assembly, demonstrating higher fragmentation in genome assembly of the latter.

The assembled transcripts were crucial for gene annotations since the *ab initio* methods predicted fragmented proteins and coding sequences (CDS). A majority of the longest *de novo* assembled transcript isoforms were from the ovarian tissue followed by the brain. Since the sequencing throughput was highest for the liver tissue in both the species, the formation of longest isoforms in the ovaries was confirmed as not an artifact of sequencing. We identified 22156 genes in *L. viridis* and 22491 genes in *L. bilineata* supported by *de novo* assembled transcripts (supplement SI-2; Table S2). The higher number of genes in *L. bilineata* was due to the fragmentation of genes onto multiple contigs, which can be resolved with scaffolding information. Compared to *A. carolinensis*, we observed an over-representation of genes in transfer RNA (tRNA) aminoacylation (Panther release 20170413, fold-enrichment=2.13-2.25, $p<0.03$) and tRNA metabolic process (Panther release 20170413, fold-enrichment=1.84-1.89, $p<0.003$) in both lacertids, indicating an expansion of tRNA-processing genes. Putative Z-chromosome linked contigs consisted of few non-coding elements (supplement SI-3). The total length of the contigs assigned to the Z-chromosome in lacertids was longer (13.5-15.6 Mbp) than the assigned Z-chromosomes of *P. vitticeps* (8 Mbp) but the number of identified genes were similar (205-221 in lacertids and 219 in *P. vitticeps*) [52].

The number of predicted members of the different non-coding RNA classes was similar in *L. viridis* and *L. bilineata* (Table S3). Compared to other selected tetrapod species, there was a substantial increase in the number of tRNAs (both functional- and pseudo-tRNAs) in the two lacertid species (Figure S5, S6). However, the number of tRNAs and pseudo-tRNAs are known to vary significantly in eukaryotes[53]. We found an over-representation of tRNA-processing genes supported by the expansion of tRNA elements in both lacertid species maintained through deletion-duplication events within lacertids. miRNA and snoRNA in the lacertids exhibited losses compared to the *A. carolinensis* (Figure S7, S8). Even though the number of snoRNA and miRNA were almost identical within lacertids, the members in each ncRNA class diversified between the two sister species. Repeat content also differed between *L. viridis* and *L. bilineata*; with the later exhibiting a gain of long-terminal repeat (LTR) elements (Table S5).

Demography and gene flow during divergence

Across all sites, mean Heterozygosity was slightly higher in *L. bilineata* than in *L. viridis* (pi=0.0014 and 0.0013 respectively). Absolute divergence per site between the two species as measured by D_{xy} was around 0.0110. These estimates correspond to a pairwise F_{ST} between *L. viridis* and *L. bilineata* of 0.77.

We inferred past divergence and gene flow between the two lacertid species using a likelihood method based on the site frequency spectrum of short sequence blocks i.e. blockwise site frequency spectrum (bSFS) [28, 54]. Since the likelihood calculation assumes no recombination within blocks and an infinite sites mutation model, we partitioned the genome into short (i.e. 200 bases) blocks. Our dataset consisted of 18,059 informative blocks (i.e. not all sites in the block are missing data), of which a mere 95 were filtered out due to the evidence of recombination (they contained both shared heterozygous sites and fixed differences which

violates the 4-gamete criterion). Less than 1% of the total sites were filtered out due to recombination reducing the average per-site heterozygosity in both species slightly (Table S6).

The counts of the four entries of the folded joint site frequency spectrum (heterozygous (het.) sites unique to A and B; het. sites shared by A and B; fixed differences) for each block defined 304 different configurations, 196 of which appeared more than once in the data.

We compared the support between different demographic scenarios (Figure 1) that assume either complete isolation or isolation with unidirectional migration between the two lacertid species and co-estimates all parameter under each model (supplement SI-4; Table S7) by maximizing the likelihood across blocks (*Mathematica* code available in supplemental file 3).

The best model supports gene flow between the two species with the assumption of two different effective population sizes (M4.1 and M4.2). The overall best model (M4.1) supports isolation between the two lacertid species with unidirectional gene flow from *L. viridis* to *L. bilineata* and fits significantly better than simpler models without gene flow (or just a single N_e parameter) (Table 1). This model (M4.1) also suggests a larger effective population size of *L. bilineata* (N_e=29,546) compared to its ancestor and *L. viridis* (N_e=14,764) (Table S7 Parameter b) and a migration rate per generation of 0.11 from *L. viridis* to *L. bilineata*.

Assuming a generation time of 3-3.5 years and a mutation rate of 1.14e-8, our estimate of the split between *L. viridis* and *L. bilineata* corresponds to 2.7-3.05 Mya (Table S8).

Detection of genomic rearrangements

We detected 20,160 genomic rearrangements or structural variants (SVs) longer than 50bp between the two lacertids (Figure 2; Table S9). These rearrangements covered 39.4Mb of the *L. viridis* genome accounting to 2.7% of the genome. These rearranged regions had a higher GC-content (47.1%) compared to regions with no detected rearrangements (44.5%). This

contrast in GC-content has been observed in genomic breakpoints, copy-number variants (CNVs) and somatic rearrangements before [55-58]. 10.8 Mb of the *L. viridis* genome (0.07%) was detected to be covered with large rearrangements affecting genes (covering the entire length of more than one gene) compared to *L. bilineata*, but these regions had a slightly smaller GC (44.4%). These large regions were enriched for RNA-directed DNA polymerase activity (22.46 fold-enrichment, p=5.11e-03).

Indels are the most frequent genomic rearrangements mainly affecting introns, repeat elements and pseudo-tRNAs (supplement SI-5; Table S10). This concedes with the observations made with respect to SVs in human populations and pigs [13, 59]. Most SVs overlapping exons cover entire exons and do not result in frame-shift mutations, with the exception of EXD2 and HERC2 which were either non-essential or their functions can be complemented by other genes (supplement SI-6).

Structural selection of ncRNAs

MicroRNAs (miRNA) were the most structurally conserved family of ncRNAs followed by small non-coding RNAs (snoRNA) (Figure S9). The four types of ncRNAs and the number of groups in each category are tabulated in Table S11 (supplement SI-7). High levels of diversity were observed in tRNAs, especially in pseudo-tRNA, which was further supported by high copy numbers of tRNAs with low conservation between the two lacertid species.

All ncRNAs with low diversity across orthologs were computationally tested for sites with positive selection in either lacertids. The divergent snoRNA families belong to the H/ACA box class which can introduce change posttranslational mechanisms and pseudouridylation between the two species [60]. SNORD61 (Small Nucleolar RNA, C/D Box 61) (Figure S10a) was inferred to have evolved under positive selection in *L. bilineata*. The human ortholog of SNORD61 occurs in the intron of a RBMX (RNA binding motif protein, X-linked gene), known

to be involved in the dosage compensation and cohesion regulator of sister chromatids [61]. Two microRNAs showed signs of positive selection in *L. viridis*: MIR6516 (mir-6516-3p) (Figure S10b) associated with urea synthesis[62] and MIR27 (mir-27a and mir27-d) (Figure S10c) which is known to play a role in regeneration and osteoblast differentiation in mice [63, 64]. However, mir-27d was absent in *L. bilineata*, so the indication of divergence between lacertids can be due to the presence of an additional MIR27 sequence in *L. viridis*. Two lincRNAs orthologs (LiNC66 and LiNC29) overlapping with conserved regions across tetrapods were structurally divergent between the lacertids, since both had high selection scores and passed the visual filtering (Figure S10d-e).

Purifying selection in lacertids

The visual opsins are pivotal for adaptation to diurnal habitats in Squamata [32, 65]. Moreover, the nocturnal *G. japonicus* lost two of the five functional opsin paralogs [34]. All five paralogs of visual opsins of *A. carolinensis* (22 transcripts from ENSEMBL) were also present in *L. viridis* and *L. bilineata* (20 transcript sequences), indicating similar diurnal adaptations. We observed high conservation of SWS1 (opsin) which is crucial for sexual selection [66, 67] and of the pigmentation protein MC1R linked to adaptive coloration [68] within the lacertids (supplement SI-8).

Varying selection pressures in protein-coding genes between lacertids

Genes involved in neuronal activity, behavior, auditory perception and female reproductive system development were conserved in the lacertid ancestor i.e. before the split between the two lacertid species (compared to five other vertebrates as background). Genes with different selection constraint between the two lacertid species (i.e. difference in purifying selection after the split between *L. viridis* and *L. bilineata*) were related to brain and neural development, embryo and cartilage development along with behavioral responses (Table S12).

The test for positive selection in either of the lacertid species was performed with branch-site model of codeml (model M2) using a sub-set of other lizards as background branches. The number of genes with positively selected sites (PSS) in different foreground branches (*L. viridis*; *L. bilineata*; or the ancestor of *L. viridis* and *L. bilineata*) are tabulated in the Table S13 (supplement SI-9). One of the genes with PSS in *L. bilineata* (STAR7) was present on Z-chromosome. The predicted ontologies of genes with PSS in either of the two lacertid species indicate potential variation in growth and developmental processes, behavioral responses (temperature and pH) and transcriptional regulation (Table S14). Three genes (NASP, PDL11 and RTKN) were positively selected in the ancestor of the lacertid branch compared to background branches that include more distant classes such as mammals and birds (supplement SI-9, Table S15).

The prostacyclin synthase (PTGIS) involved in regeneration through prostaglandin synthesis is positively selected in *A. carolinensis* and *G. japonicus* [34]. This gene evolved under positive selection in the lacertid ancestor with *A. carolinensis* and *G. japonicus* as the background, hinting at evolutionary changes in regenerative mechanisms between lizards.

Diversification of UV-responsive genes

We identified three paralogs of the hyaluronidases (HYAL1, HYAL2 and HYAL4) in both the lacertid genomes. Two genes (STIK1 and HYAL2) coding for proteins in the extra-cellular matrix of the skin reacting to UV-B light (GO:0071493) [69] were positively selected in ancestral branch of the lacertids, while the HYAL1 paralog was positively selected in *L. viridis* (supplement SI-8). Arylsulfatase gene (ARSB) which is involved in the chondroitin sulfate biosynthesis pathway along with HYAL was also positively selected in *L. viridis*. Significant pathway enrichment of chondroitin sulfate biosynthesis was observed for PSGs in *L. viridis* (p=2.6e-06, q=1.3e-05).

Divergence of Kruppel-type zinc-finger proteins and species-specific alternative splicing

KRAB-ZNFs or KZNFs (zinc finger proteins with a *Krüppel*-associated box (KRAB)) are transcriptional regulators are confined to tetrapod vertebrates [70] and are known to play a role in reproductive isolation through binding domain differences as well as in the shaping of recombination landscape across the primate genomes [7, 71-74]. To investigate the role of KRAB-ZNFs in the reproductive isolation of our two lacertid species, we compared the KZNF orthologs for differences in their DNA-binding domains and found divergent six orthologous C2H2 zinc-finger proteins (supplement SI-10). Interestingly, all of these KZNFs had their longest transcripts assembled from ovarian tissues (Table S16) and these were not tissue-specific since they were expressed in all the five tissues.

The genes with significantly varying splice forms between the lacertid species were enriched for spliceosomal activity (supplement SI-10). These differences in alternative splicing were predicted based on the presence of alternative splice junction read support in all the five tissue transcriptomes (brain, heart, liver, kidneys and ovaries).

Impact of rearrangements on sequence evolution

Deletions are the most frequent type of SVs in the genome and occurred on both positively selected and neutrally evolving genes. Duplications and insertions only occurred in genes evolving neutrally while deletions and inversions occurred in genes irrespective of their selective regime. The ratio of regions with rearrangements or SVs to those with no detected rearrangements was different between genes under positive selection and neutrally evolving genes (Boschloo's exact test, two-sided; difference in proportion=0.125, p=0.06). This implies low significance in the association of genes under positive selection (PSGs) with SVs. Since this can be due to indels over-showing other categories of SVs due to higher abundance, we tested the individual effect of each SV category with positive selection separately. The

association between PSGs and each SV category (or rearrangements) was performed with independent Boschloo exact-tests (Table S17). Tests between different categories of SVs over PSGs and neutrally evolving genes (NGs) showed significantly higher occurrence of inversions with PSGs compared to other SV categories (p=0.028). These inversions overlapping with PSGs were independent events on different contigs. We also observed a significant occurrence of inversions with PSGs over NGs compared to both non-rearranged regions (p=0.009) and collinear regions (p=0.006).

Discussion

We provide the assembled genomes of two closely-related lacertid species, *L. viridis* and *L. bilineata*, investigated the population history and determined the patterns of genomic divergence between these species.

The error correction of PacBio reads (15-18% error rate) with Illumina data (<0.1% error rate) provided a clear improvement in the genome assembly. The assembly contiguity was highest with partial error correction of PacBio reads (without splitting at chimeric junctions) followed by hybrid assembly through DBG2OLC implementing removal of chimeric joins. This hybrid assembly strategy aided in generating high quality contig-level genomes with moderate genome coverages (~35X Illumina and ~15X PacBio). The quality of the lacertid genome assemblies was higher than the available lizard genomes (Table S1).

The estimated time of split between *L. viridis* and *L. bilineata* was 2.7-3.05 Mya which is similar to the earlier predicted split time of 2.6-3.4 Mya based on mitochondrial genomes which are 95% identical [41, 75]. The genetic divergence between the two lacertid species (F_{ST}=0.77) is slightly higher than between species divergence in primates (F_{ST}=0.54-0.74 between

chimpanzees and bonobos) and within species divergence of *L. agilis* populations (F_{ST}=0.299). Therefore, our results support the separate species status of *L. viridis* and *L. bilineata*.

The best demographic model confirmed unidirectional gene flow predominantly in the direction from *L. viridis* to *L. bilineata*. We also infer a higher effective population size for *L. bilineata* which can be explained by greater population subdivision compared to *L. viridis* [42, 76, 77]. The eastern clade (*L. viridis*) possesses smaller effective population size indicating lower genetic diversity in our sampled population (Hungary), in line with previous studies. Another possibility is that *L. viridis* is strongly structured into meta-populations that are affected by local extinction and recolonization events[78].

Diversity within various ncRNA classes and adaptive differences in ncRNA orthologs capable of altering their secondary structures are leading factors contributing to evolutionary divergence since varying ncRNA structures imply functional changes [25]. Copy number variation and differences in the content of miRNA families hints at variability in gene regulatory networks between the lacertid sister species. Species-specific splicing mechanisms can be attributed to the losses of snoRNA families (SNORA 17 and SNORA20) in *L. bilineata* and structural changes in SNORD61 whose human ortholog occurs in RBMX gene (catalytic site 2 spliceosome) involved in dosage compensation [79]. This is supported by significant enrichment of alternative splicing differences for spliceosomal complex related genes. Although differential alternative splicing was observed in all extracted tissues of both species (without reference bias), this needs to be further investigated with more biological replicates.

Positive selection of sites in NASP and PDLIM1 compared to distant background branches including mammals and birds indicate disparate evolutionary changes in both *L. viridis* and *L. bilineata* with regard to reproductive processes i.e. spermatogenesis, fertilization and embryo implantation [80-83]. Positive selection of sites in the genes of either lacertid species after their

split from a common ancestor indicates adaptive differences leading to speciation if selection occurred before complete reproductive isolation [84-86].

UV-reflectance of plumages in birds an important trait in the sexual selection of morphologically similar sibling species [87]. Sexual selection in *L. viridis* has been linked to UV-response, males with more UV-reflective patches on the skin are preferably selected by the females [88, 89]. Hyaluronidases, known to be differentially expressed on exposure to UV-B in the skin of mice [69, 90, 91] evolve rapidly in lacertids. We speculate that differential cutaneous response as a result of adaptive differences in chondroitin sulfate (CS) biosynthesis pathway drives preferential mating in these lacertid species.

The divergence of transcription factors, especially differences in DNA-binding regions of KZNFs as observed here, might have contributed to the reduced reproductive success between lacertid species. This receives further support from adaptive differences in the transcription factors (UBIP1 and RPA2) crucial for spermatogonia formation [92, 93]. Varying levels of purifying selection in genes influencing forebrain development and behavior indicate dissimilarities between *L. viridis* and *L. bilineata* [94-98]. These differences can arise from adaptations to varying ecological habitats and environmental conditions [43]. Adaptive changes in genes involved in habitat preference, behavior and viability after the split of *L. viridis* and *L. bilineata* seem to be elemental in their divergence. Similar observations were made between species of anoles through selective differences in genes related to behavior and brain development [40].

Genomic regions harboring inversions are known to suppress recombination in heterokaryotypes [99] facilitating speciation in the presence of gene flow. Genomic inversions between the two lacertids are significantly associated with positively selected genes and may play a role in reproductive isolation. In particular, adaptation of genes related to cognitive and

reproductive genes (GPR155 and TDRD3) may contribute to reproductive isolation through association with inversions. Despite observing association of inversions with PSGs which can lead to reproductive isolation, we are currently unaware of fixed inversion differences between lacertids.

SV-polymorphisms also occur within populations [13] and sequencing of multiple individuals from different populations of each species is required before drawing far-reaching conclusions. Assessing the frequencies of these inversions within and between lacertid populations would be crucial in understanding their relevance to speciation. In addition to a detailed analysis of the demographic history and evolutionary scenario of European green lizards, our study provides valuable data that will help establish conservation guidelines for lacertids which are declining [100] due to habitat loss.

Conclusions

We assembled the first high quality genomes of two closely-related species of European green lizards produced with a cost-effective strategy. Genes related to with transcriptional regulation, behavior, neural and reproductive development have diversified the most between the lacertids. Species-specific diversity of ncRNAs, adaptive evolution in regulatory elements and transcription factors (especially KZNFs) indicate variation in gene regulatory networks pointing to reproductive isolation between the two species. Preferential mate selection between lacertids is driven by adaptation of genes responsible for differential cutaneous response to UV-exposure. Reproductive isolation between *L. viridis* and *L. bilineata* seems to be also driven by accumulated divergence through inversions and their association with genes under positive selection. Altogether, we provide a comprehensive study of the evolutionary history; genic,

structural and regulatory differences between the genomes of two closely-related lacertid species.

Materials and Methods

Sampling

Two adult females were sampled for this study, a *L. viridis* from Tokaj, north-eastern Hungary (21.39775^{o}E, 48.11363^{o}N) (September 2013) and a *L. bilineata* from Malain, France (4°48'2.01"E, 47°21'16.27"N) (July 2014). There is no known morphological variation between the individuals of the two species (Figure S1). These represent two of the four main clades within the *L. viridis* complex [41, 42, 46, 101]. The specimens were transported in a cotton bag and kept at room temperature over night to avoid extreme stress responses. Tissues from the brain, heart, liver, kidney and ovaries were dissected for tissue-specific transcriptome sequencing and the remaining body tissues were stored separately at -80°C.

Whole-genome and transcriptome sequencing

Tail tissue from each sample was digested with proteinase K and genomic DNA was extracted using a chloroform-based method [102]. The whole genome was sequenced using both short (Illumina) and long read (PacBio) sequencing techniques. Short-read libraries with insert sizes of 380bp and 450bp were prepared for each individual separately. The Illumina paired-end sequences were double-indexed using a multiplexing sequencing protocol [103, 104] on a HiSeq2500. SMRTbell™ template library was prepared according to the instructions from PacificBiosciences, Menlo Parl, CA, USA, following the Procedure and Checklist – Greater Than 10 kb Template Preparation. Briefly, for preparation of 15kb libraries 10µg (*L. bilineata*) and 20µg (*L. viridis*) genomic DNA was damage-repaired twice, end-repaired and ligated

overnight to hairpin adapters applying components from the DNA/Polymerase Binding Kit P6 from Pacific BioSciences, Menlo Park, CA, USA. Reactions were carried out according the manufacturer's instructions. BluePippin™ Size-Selection to greater than 15kb was performed according to the manufacturer's instructions (Sage Science, Beverly, MA, USA). Conditions for annealing of sequencing primers and binding of polymerase to purified SMRTbell™ template were assessed with the Calculator in RS Remote, PacificBiosciences, Menlo Park, CA, USA. Long-read sequencing was carried out for both genomes with 20 SMRT Cells applying P6-C4 chemistry on a PacBio RS-*II* sequencer. Average PacBio read lengths of 14kb and 12kb were retrieved for *L. viridis* and *L. bilineata*, respectively.

RNA from each tissue was extracted using Trizol Reagent (Life Technologies, Carlsbad, CA, USA) and purified with the RNeasy® Mini Kit (Qiagen, Hilden, Germany). The mRNA was purified using the Dynabeads® mRNA Purification Kit (Life Technologies, Carlsbad, CA, USA). The purity and concentration of RNA and cDNA were checked using Nanodrop and Bioanalyzer 2100 (Agilent Technologies, CA, USA) and fragments of length 200-250bp were obtained using Ambion® RNA fragmentation reagent. The first and second strands of cDNA were synthesized using random hexamer primers with SuperScript® II reverse transcriptase (Life Technologies, Carlsbad, CA, USA) and DNA Polymerase I with RNase H treatment (Life Technologies, Carlsbad, CA, USA) respectively.

L. viridis was sequenced on a single lane for a more elaborated estimation of the genome size and repeat content. In order to avoid lane- and run-biases, sequencing was distributed over three lanes with all genomes and transcriptomes.

Non-coding RNA (ncRNA) annotation and Repeat analysis

Small ncRNAs were annotated on the genomic contigs by performing an infernal cmscan (V1.1.1) using the RFAM covariance models as input and homologous ncRNA genes were filtered with a cut-off of 1e-06 [105, 106].

Additionally, ncRNA class-specific annotation methods were used for tRNAs, snoRNAs and miRNAs. tRNAs were annotated using tRNAscan-SE with default parameters [107]. The BLAST-based snoStrip pipeline [108] was used to annotate snoRNAs. A comprehensive set of snoRNAs from vertebrates and aves were used as query set [109]. To detect miRNAs, the avian set of miRNAs were used as query sequences for a BLAST search in the lizard genomes. All resulting blast hits were filtered for conservation of the seed region. The annotated snoRNAs and miRNAs in lacertids were validated by blast searches against this reference database and mature miRNA sequence homologies were used. In the case of overlapping miRNA and snoRNA annotations, both were retained as it is known that snoRNAs can be processed into small derived RNAs (sdRNAs) from miRNA-like RNAs [110]. Putative lincRNAs were predicted based on the transcripts with no coding potential as assessed by Transdecoder [111] and mapping on their respective genome without chimeric paths. Furthermore, only the conserved lincRNAs with one-to-one orthologs between lacertids were retained.

For comparison, ncRNA families (except lincRNA) were also annotated in other selected sauropsid genomes. A reference database was created using sequenced and annotated genomes from reptiles, aves and other vertebrates. The program ePoPe [112] was used to understand the evolution of snoRNAs and miRNAs in the lacertids through the construction of phylogenetic trees based on the gains and losses of ncRNA families.

The Repeatmodeler pipeline [113] was used to predict repeats in the genomes of lacertids. The predicted repeat-families were used as initial libraries for *de novo* annotation of repeats using Repeatmasker [114]. The evolution of these repeats was investigated using the repeat library available for tetrapod species (Database: 20140131).

Population histories, gene flow and coalescence

To infer the history of divergence and gene flow between *L. viridis* and *L. bilineata* we used the blockwise composite likelihood approach. We analytically computed the probabilities of mutational configurations in blocks of fixed length using the blockwise site frequency spectrum (bSFS) framework [28].

We mapped the illumina reads from *L. viridis* and *L. bilineata* to the *L. viridis* genome as reference with BWA mem [115]. The homozygosity/heterozygosity of each site in both lacertids was predicted based on the reference genome with freebayes [116] with a minimum read support of 5. For each block of length 200 bp, we counted the number of the four mutation types defined by the joint SFS (Figure 3). We then summarized the frequency of each polymorphism pattern across all blocks [117, 118]. This data summary is referred to as distribution of bSFS.

Blocks containing both fixed differences and shared heterozygous sites, violate the 4-gametes criterion and were removed given the assumption of no recombination within blocks. To correct for the extent of linkage disequilibrium which includes correlation between adjacent blocks, we followed a conservative approach for the correction of maximum-likelihood of each model in every 114 blocks. We fitted seven different demographic scenarios (Figure 2): divergence with the same effective population sizes in two separated populations and their ancestor (M1), divergence with different effective population sizes in one lineage compared to its ancestor (M2.1, M2.2), divergence with continuous unidirectional gene flow with fixed effective population size (M3.1, M3.2), and divergence with continuous unidirectional gene flow with different effective population sizes in one lineage compared to its ancestor (M4.1, M4.2). The best fitting scenario was chosen based on the difference in composite Log likelihoods between models.

To calculate the time of split between *L. viridis* and *L. bilineata*, we assumed a mutation rate of 1.14e-08 based on the within-lineage divergence estimate of *L. viridis* from the pairwise distances of cytochrome *b* gene [42]. This assumption was similar to the estimation of mutation rate in *A. carolinensis* [119]. No published estimate of the generation time for *L. viridis* and *L. bilineata*. However, this can be calculated approximately as the mean age of the mothers of all offspring [120] given the age structure data by Elbing [121] and Saint Girons *et al*. [122] for three German populations of *L. viridis* and two French populations of *L. bilineata,* respectively. In captivity, females that breed for the first time lay on overage 8.5 eggs, whereas older females lay 11.1 eggs [123]. Given this data we estimated a mean generation length of 3.6 and 2.9 years for *L. viridis* and *L. bilineata,* respectively. We therefore assumed a generation time of about 3-3.5 years for both species.

Detection of genomic rearrangements from read-based pipelines and syntenic blocks

Genomic rearrangements between the lacertids were detected based on both read-based methods and syntenic blocks information. *L. viridis* was used as the reference genome since the assembly was more contiguous. Genomic reads from *L. bilineata* were used as the query and the reads of *L. viridis* mapped against the reference were used as control.

Read-based pipelines: Genomic rearrangements were detected between lacertids using read mapping based methods for Illumina paired-end reads and for PacBio-reads separately, followed by SV callers specifically developed to deal with short and long read sequences, respectively. In both approaches, reads of *L. bilineata* (query) and of *L. viridis* (control) were separately mapped against the same reference (*L. viridis*).

The alignment of Illumina reads was carried out with BWA mem [115] and rearrangements were detected with MetaSV [124] pipeline which uses Breakdancer [125] to infer structural variants (SVs) using paired-end read information, CNVnator [126] to predict copy-number

variants (CNVs) from abnormal read-coverages and Pindel [127] to detect large SV-related breakpoint events. The insert-size was estimated as 400±50 from one million observations based on the alignment of paired-end Illumina reads. A minimum support of five reads and mapping quality of 30 was set as the threshold to support SVs from Breakdancer. A bin-size of 500 was used to run CNVnator and only precise SV-events were called. While for Pindel, only variants with minimum read support of 5 paired-reads were used. MetaSV pipeline was used to merge the SVs from these three different SV-callers and local *de novo* assemblies were constructed using the ABYSS assembler for insertions. In order to maintain a high level of sensitivity and specificity (>90%) in the detection of SVs, only the rearrangements called with a minimum support of 8 uniquely mapped paired-end reads were used for further analyses [128].

The PacBio reads were aligned to the reference with NGMLR and the alignment was fed to Sniffles SV-caller [12] to call variants with a minimum support of seven reads (atleast half of the PacBio genome coverage of 14X).

Syntenic blocks approach: In addition to read-based methods, rearrangements were also detected from the blocks of synteny obtained through the UCSC pipeline [129]. The alignments were converted to single-coverage genomes using single_cov2 of the MultiZ pipeline [130] to avoid spurious assignments. Strand changes within syntenic blocks were clustered as inversions (I) based on the orientation of the successive (I+1) and preceding (I-1) blocks. Regions with missing bases in the query alone were predicted to be deletions while gaps in the reference genome alone were considered as insertions. Additionally, Hierarchical Alignment (HAL) format [131] of the single-coverage genomes was used to predict rearrangements with halBranchMutations tool. This tool generates annotations for the location of rearrangements based on the branch of interest in the HAL file (between *L. viridis* and *L. bilineata* in our case). The events detected with in both directions i.e. *L. viridis* reference with *L. bilineata* as query

and *L. bilineata* reference and *L. viridis* as query were retained. The length threshold was set to 50bp and the predicted rearrangements were filtered based on quality to reduce false-positives (supplement SM-7).

Segmental duplications in the two lacertid species were detected by self-aligning the two genomes separately with chained LASTZ[132] (step=9, H=3000, K=5000). High identity matches (90% identity) within each genome of 1kb or more were defined as segmental duplications.

Structural selection in non-coding RNAs (ncRNA)

The predicted ncRNAs (miRNA, snoRNA, tRNA and lincRNA) in lacertids were tested for structural selection (selection of sites acting on secondary structure in either of the lacertids) with *G. japonicus* as outgroup. We used the Selection on the Secondary Structure test (SSS-test) [133], a statistical test that assigns selection scores for each given sequence based on the comparison between the structure of the given sequence and the structure of group consensus. It also provides a diversity value for the family that indicates its structural conservation. The diversity value (d-score) is the family's median vase-pair distance to its consensus. The miRNAs, snoRNAs and tRNAs were divided into sub-groups based on their families or their anti-codon sequences, and only those sub-groups with at least three sequences were tested. The groups that exhibited high structural diversity (median base pair distance to the consensus, $d \geq 10.0$) were excluded from further analyses.

A ncRNA structural test to detect positively selected structures is only appropriate for structurally conserved groups. Low d-score values ($d<10.0$) were used to distinguish conservation chosen based on structural uniformity of the groups. This cut-off was based on the visual inspection of the secondary structures of families with d-scores of 1 to 20. Secondary structures of ncRNA sequences were predicted using RNAfold [134]. In a similar fashion,

structures with selection scores of 0 to 30 were visually compared to the structure of their group consensus. High selection scores (s≥10.0) were used to predict the positively selected sequences of small ncRNAs. Secondary structures with high selection scores were manually inspected to remove false positives. Specifically, the candidates with structures of low stability or those fundamentally dissimilar to the family consensus indicating loss of function were excluded.

The selection test was adapted for lincRNAs and performed only on the two lacertid species without any outgroup since lincRNA annotations of other closely-related species were unavailable. Since positive selection of secondary structure cannot be determined without outgroups, we instead detected divergence of lincRNA structure within the lacertids. Local conserved structure blocks were predicted for the orthologous lincRNA families and these blocks were subjected to an adaptation of SSS-test based on local structures. The structural selection for lincRNAs was assessed locally, since most base-pairings occur between nucleotides within a short distance [133, 135]. Local blocks of high structural diversity were excluded from further analysis. Since outgroups were not used for lincRNAs, a lower selection score threshold (s≥4.0) was applied to detect divergent candidates which were visually inspected later to exclude false-positives.

Ortholog prediction and selection tests

In order to investigate the selection pressure in the lacertid branch (ancestor of *L. viridis* and *L. bilineata*) compared to other vertebrates, the coding sequences (CDS) of five species, namely anole lizard (*Anolis carolinensis*), chicken (*Gallus gallus*), frog (*Xenopus tropicalis*), spotted gar fish (*Lepisosteus oculatus*) and human (*Homo sapiens*) were downloaded from the Ensembl database version 83 [136]. To keep the data consistent and avoid re-annotations, the CDS annotations were also extracted from the Ensembl database. The orthologs between the coding sequences of the species were identified with ProteinOrtho V5 using the synteny option to reduce false orthologs assignments. The output was converted to run the POTION pipeline

[137] which tests for selection acting on protein coding genes. Only the single-copy orthologs in each species was retained for each orthologous group.

The protein identity filtering in POTION was set to 30% in each orthologous group and sequence size limits to more than 10 times or less than 0.2 of the median size in the group. Only groups with at least 4 species were retained. The sequences in each orthologous group (after filtering paralogs) were aligned, gap trimmed, phylogenetic trees were constructed and groups with recombinants were excluded from the selection tests. The intermediates files from the POTION pipeline were used to generate unrooted trees with lacertids (*L. viridis* and *L. bilineata*) in the foreground branches. The remaining species were used as the background to test for positive-selection using branch-site model of codeml within the PAML package[138]. A likelihood ratio test (LRT) based on χ^2 distribution was used to detect genes with significant positive selection followed by multiple testing through Benjamini–Hochberg (BH) procedure. Genes with $p<0.05$ and $q<0.05$ were retained and referred to as being positively selected in the lacertid branch.

To detect adaptive evolution through positive selection within either lacertids, additional tests (PAML branch-site models) were performed with less distant outgroups using a set of five lizard species, namely *L. viridis, L. bilineata, Anolis carolinensis, Gekko japonicus* and *Pogona vitticeps*. The single-copy orthologs were identified with ProteinOrtho with a minimum protein identity of 70%, e-value of 1e-06 and minimum similarity of 0.99 for additional hits. The orthologous coding sequences from the five lizard species were aligned with MACSE while accounting for frame-shifts and the stop codon at the end of the sequence was removed. Unrooted trees were generated with three different foreground branches: i) lacertids (*L. viridis* and *L. bilineata*) ii) *L. viridis* alone and iii) *L. bilineata* alone. The rest of the workflow for detection of recombinants, removal of gaps and codeml tests was similar to the POTION pipeline followed by filtering for significant candidate genes ($p<0.05$, $q<0.05$). In order to

avoid false predictions of positively selected sites (PSS) at the beginning or towards the end of alignments, where mismatches were allowed, the candidate genes predicted to contain PSS in either species were visually inspected.

Data Access

The genome assembly, transcript data, DNA and RNA sequencing reads have been deposited in the European Nucleotide Archive under the Bioproject PRJEB24178.

GCA_900245905 - *L. viridis* genome assembly

GCA_900245895 - *L. bilineata* genome assembly

The transcript assemblies, genome browser and online BLAST databases for the lacertid data are hosted at http://lacerta.bioinf.uni-leipzig.de

Genome annotations, variant calls (VCFs) and other supporting datasets are available at http://doi.org/10.5281/zenodo.1219810

Declarations

Acknowledgements

The authors thank Ms. A. Grimm for the sample collection and providing the pictures of the lacertids; Dr. C. Helm, Dr. D. Bernhard, Dr. M. Gerth, Mr. R. Wolf and Mr. S. Schaffer for their help in the laboratory; Prof. S. Hoffmann and Dr. T. Hackl for valuable discussions and Mr. S. Peyrégne for helpful suggestions and proofreading. The specimen from Hungary was kindly collected and provided by the team of Dr. Szabolcs Lengyel, Centre for Ecological

Research, Hungarian Academy of Science. Capture permit (No. 13778-7/ 2013) was issued by the North Hungarian Environmental Protection, Nature Conservation and Water Management Inspectorate. Capture permit (No. 36) for the French specimen was issued by the Préfet de la Côte d'Or. RF was financed by FCT [grant number SFRH/BPD/89313/2012] and is currently funded by the European Union's Horizon 2020 research and innovation programme, under the Marie Sklodowska-Curie grant agreement number 706376.

Funding

This project was funded by the Deutsche Forschungsgemeinschaft (FZT 118).

Declaration of interest

The authors report no conflicts of interest. The authors alone are responsible for the content and writing of the paper.

Additional files

Additional file 1: This supplement contains methods SM1-SM12, information SI1-SI10, Figures S1–S10, Tables S1–S17 and References.

Additional file 2: The figure for the contig clusters in lacertids generated from synteny information between *L. viridis* and *L. bilineata*.

Additional file 3,4: *Mathematica* notebooks containing the code used and other supporting information from the demography analysis of *L. viridis* and *L. bilineata*.

References

1. Coyne JA, Orr HA: **Speciation**: Sinauer Associates; 2004.
2. Orr HA: **Dobzhansky, Bateson, and the Genetics of Speciation**. *Genetics* 1996, **144**(4):1331-1335.
3. Presgraves DC: **The molecular evolutionary basis of species formation**. *Nat Rev Genet* 2010, **11**(3):175-180.
4. Charlesworth B, Coyne JA, H. Barton N: **The Relative Rates of Evolution of Sex Chromosomes and Autosomes**. *American naturalist* 1987, **130**(1):113-146.
5. Masly JP, Presgraves DC: **High-resolution genome-wide dissection of the two rules of speciation in Drosophila**. *PLoS Biol* 2007, **5**(9):e243.
6. Mack KL, Campbell P, Nachman MW: **Gene regulation and speciation in house mice**. *Genome Res* 2016, **26**(4):451-461.
7. Nowick K, Carneiro M, Faria R: **A prominent role of KRAB-ZNF transcription factors in mammalian speciation?** *Trends Genet* 2013, **29**(3):130-139.
8. Ortiz-Barrientos D, Kane NC: **Meeting review: American Genetics Association Symposium on the genetics of speciation**. *Mol Ecol* 2007, **16**(14):2852-2854.
9. Mayr E: **Wu's genic view of speciation**. *Journal of Evolutionary Biology* 2001, **14**(6):866-867.
10. Chung W, Ting C-T: **Genes and speciation**. *Nature reviews Genetics* 2004, **5**(2):114.
11. Seehausen O, Butlin RK, Keller I, Wagner CE, Boughman JW, Hohenlohe PA, Peichel CL, Saetre GP, Bank C, Brannstrom A *et al*: **Genomics and the origin of species**. *Nat Rev Genet* 2014, **15**(3):176-192.
12. Sedlazeck FJ, Rescheneder P, Smolka M, Fang H, Nattestad M, von Haeseler A, Schatz MC: **Accurate detection of complex structural variations using single-molecule sequencing**. *Nature Methods* 2018.
13. Sudmant PH, Rausch T, Gardner EJ, Handsaker RE, Abyzov A, Huddleston J, Zhang Y, Ye K, Jun G, Hsi-Yang Fritz M *et al*: **An integrated map of structural variation in 2,504 human genomes**. *Nature* 2015, **526**(7571):75-81.
14. Alkan C, Coe BP, Eichler EE: **Genome structural variation discovery and genotyping**. *Nat Rev Genet* 2011, **12**(5):363-376.
15. Davey JW, Barker SL, Rastas PM, Pinharanda A, Martin SH, Durbin R, McMillan WO, Merrill RM, Jiggins CD: **No evidence for maintenance of a sympatric Heliconius species barrier by chromosomal inversions**. *Evolution Letters* 2017, **1**(3):138-154.
16. Noor MA, Grams KL, Bertucci LA, Reiland J: **Chromosomal inversions and the reproductive isolation of species**. *Proc Natl Acad Sci U S A* 2001, **98**(21):12084-12088.
17. Navarro A, Barton NH: **Chromosomal speciation and molecular divergence--accelerated evolution in rearranged chromosomes**. *Science* 2003, **300**(5617):321-324.
18. Kirkpatrick M: **How and why chromosome inversions evolve**. *PLoS Biol* 2010, **8**(9).
19. McGaugh SE, Noor MA: **Genomic impacts of chromosomal inversions in parapatric Drosophila species**. *Philos Trans R Soc Lond B Biol Sci* 2012, **367**(1587):422-429.
20. Lohse K, Clarke M, Ritchie MG, Etges WJ: **Genome-wide tests for introgression between cactophilic Drosophila implicate a role of inversions during speciation**. *Evolution* 2015, **69**(5):1178-1190.
21. Navarro A, Barton NH: **Accumulating postzygotic isolation genes in parapatry: a new twist on chromosomal speciation**. *Evolution* 2003, **57**(3):447-459.
22. Kirkpatrick M, Barton N: **Chromosome inversions, local adaptation and speciation**. *Genetics* 2006, **173**(1):419-434.
23. Frías-Lasserre D, Villagra CA: **The Importance of ncRNAs as Epigenetic Mechanisms in Phenotypic Variation and Organic Evolution**. *Frontiers in Microbiology* 2017, **8**:2483.
24. Walter Costa MB, Honer Zu Siederdissen C, Tulpan D, Stadler PF, Nowick K: **Temporal ordering of substitutions in RNA evolution: Uncovering the structural evolution of the Human Accelerated Region 1**. *J Theor Biol* 2017, **438**:143-150.

25. Bhartiya D, Scaria V: **Genomic variations in non-coding RNAs: Structure, function and regulation**. *Genomics* 2016, **107**(2-3):59-68.
26. Gardner PP, Fasold M, Burge SW, Ninova M, Hertel J, Kehr S, Steeves TE, Griffiths-Jones S, Stadler PF: **Conservation and losses of non-coding RNAs in avian genomes**. *PLoS One* 2015, **10**(3):e0121797.
27. Sousa V, Hey J: **Understanding the Origin of Species with Genome-Scale Data: the Role of Gene Flow**. *Nature reviews Genetics* 2013, **14**(6):404-414.
28. Lohse K, Chmelik M, Martin SH, Barton NH: **Efficient Strategies for Calculating Blockwise Likelihoods Under the Coalescent**. *Genetics* 2016, **202**(2):775-786.
29. Ravinet M, Faria R, Butlin RK, Galindo J, Bierne N, Rafajlović M, Noor MAF, Mehlig B, Westram AM: **Interpreting the genomic landscape of speciation: a road map for finding barriers to gene flow**. *Journal of Evolutionary Biology* 2017, **30**(8):1450-1477.
30. Camargo A, Sinervo B, Sites JW, Jr.: **Lizards as model organisms for linking phylogeographic and speciation studies**. *Mol Ecol* 2010, **19**(16):3250-3270.
31. Alfoldi J, Di Palma F, Grabherr M, Williams C, Kong L, Mauceli E, Russell P, Lowe CB, Glor RE, Jaffe JD *et al*: **The genome of the green anole lizard and a comparative analysis with birds and mammals**. *Nature* 2011, **477**(7366):587-591.
32. Gao J, Li Q, Wang Z, Zhou Y, Martelli P, Li F, Xiong Z, Wang J, Yang H, Zhang G: **Sequencing, de novo assembling, and annotating the genome of the endangered Chinese crocodile lizard Shinisaurus crocodilurus**. *GigaScience* 2017, **6**(7):1-6.
33. Rice ES, Kohno S, John JS, Pham S, Howard J, Lareau LF, O'Connell BL, Hickey G, Armstrong J, Deran A *et al*: **Improved genome assembly of American alligator genome reveals conserved architecture of estrogen signaling**. *Genome Res* 2017, **27**(5):686-696.
34. Liu Y, Zhou Q, Wang Y, Luo L, Yang J, Yang L, Liu M, Li Y, Qian T, Zheng Y *et al*: **Gekko japonicus genome reveals evolution of adhesive toe pads and tail regeneration**. 2015, **6**:10033.
35. Georges A, Li Q, Lian J, O'Meally D, Deakin J, Wang Z, Zhang P, Fujita M, Patel HR, Holleley CE *et al*: **High-coverage sequencing and annotated assembly of the genome of the Australian dragon lizard Pogona vitticeps**. *GigaScience* 2015, **4**(1):45.
36. Castoe T, Koning A, Hall K, Card D, Schield D, Fujita M: **The Burmese python genome reveals the molecular basis for extreme adaptation in snakes**. *Proc Natl Acad Sci U S A* 2013, **110**.
37. Vonk F, Casewell N, Henkel C, Heimberg A, Jansen H, McCleary R: **The king cobra genome reveals dynamic gene evolution and adaptation in the snake venom system**. *Proc Natl Acad Sci U S A* 2013, **110**.
38. Shaffer H, Minx P, Warren D, Shedlock A, Thomson R, Valenzuela N: **The western painted turtle genome, a model for the evolution of extreme physiological adaptations in a slowly evolving lineage**. *Genome Biol* 2013, **14**.
39. Wang Z, Pascual-Anaya J, Zadissa A, Li W, Niimura Y, Huang Z: **The draft genomes of soft-shell turtle and green sea turtle yield insights into the development and evolution of the turtle-specific body plan**. *Nat Genet* 2013, **45**.
40. Tollis M, Hutchins ED, Stapley J, Rupp SM, Eckalbar WL, Maayan I, Lasku E, Infante CR, Dennis SR, Robertson JA *et al*: **Comparative Genomics Reveals Accelerated Evolution in Conserved Pathways during the Diversification of Anole Lizards**. *Genome Biology and Evolution* 2018, **10**(2):489-506.
41. Böhme MU, Fritz U, Kotenko T, Georg D, Ljubisavljević K, Tzankov N, Berendonk TU: **Phylogeography and cryptic variation within the Lacerta viridis complex (Lacertidae, Reptilia)**. *Zoologica Scripta* 2007, **36**(2):119-131.
42. Marzahn E, Mayer W, Joger U, Ilgaz Ç, Jablonski D, Kindler C, Kumlutaş Y, Nistri A, Schneeweiss N, Vamberger M *et al*: **Phylogeography of the Lacerta viridis complex: mitochondrial and nuclear markers provide taxonomic insights**. *Journal of Zoological Systematics and Evolutionary Research* 2016, **54**(2):85-105.

43. Tvrtkovi N, Lazar B, Tome S, Grbac I: **The western green lizard *Lacerta* (*viridis*) *bilineata* Daudin, 1804 (Sauria: Lacertidae) in Slovenia and Croatia**. *Natura Croatica* 1998, **7**(4):363-369.
44. Amann T, Rykena S, Joger U, Nettmann HK, Veith M: **Zur artlichen Trennung von Lacerta bilineata DAUDIN, 1802 und L. viridis (LAURENTI, 1768)** *Salamandra* 1997, **33**(4):255-268.
45. Joger U, Amann T, Veith M: **Phylogeographie und genetische differenzierung im Lacerta viridis/bilineata komplex**. *Mertensiella* 2001, **13**:60-68.
46. Rykena S: **Kreuzungsexperimente zur Prüfung der Artgrenzen im Genus Lacerta sensu stricto**. *Zoosystematics And Evolution* 1991, **67**(1):55-68.
47. Olmo E: **Rate of chromosome changes and speciation in reptiles**. *Genetica* 2005, **125**(2-3):185-203.
48. Kupriyanova L, Kuksin A, Odierna G: **Karyotype, chromosome structure, reproductive modalities of three Southern Eurasian populations of the common lacertid lizard, Zootoca vivipara (Jacquin, 1787)**. *Acta Herpetologica; Vol 3, No 2 (2008)* 2008.
49. Liu H, Chang LH, Sun Y, Lu X, Stubbs L: **Deep Vertebrate Roots for Mammalian Zinc Finger Transcription Factor Subfamilies**. *Genome Biol Evol* 2014, **6**(3):510-525.
50. Chiarelli AB, Capanna E: **Cytotaxonomy and Vertebrate Evolution Edited by A.B. Chiarelli and E. Capanna**; 1973.
51. Olmo E, Odierna G, Cobror O: **C-band variability and phylogeny of Lacertidae**. *Genetica* 1986, **71**:63-74.
52. Deakin JE, Edwards MJ, Patel H, O'Meally D, Lian J, Stenhouse R, Ryan S, Livernois AM, Azad B, Holleley CE *et al*: **Anchoring genome sequence to chromosomes of the central bearded dragon (Pogona vitticeps) enables reconstruction of ancestral squamate macrochromosomes and identifies sequence content of the Z chromosome**. *BMC Genomics* 2016, **17**:447.
53. Bermudez-Santana C, Attolini CS-O, Kirsten T, Engelhardt J, Prohaska SJ, Steigele S, Stadler PF: **Genomic organization of eukaryotic tRNAs**. *BMC Genomics* 2010, **11**(1):270.
54. Bunnefeld L, Frantz LA, Lohse K: **Inferring Bottlenecks from Genome-Wide Samples of Short Sequence Blocks**. *Genetics* 2015, **201**(3):1157-1169.
55. Bauters M, Van Esch H, Friez MJ, Boespflug-Tanguy O, Zenker M, Vianna-Morgante AM, Rosenberg C, Ignatius J, Raynaud M, Hollanders K *et al*: **Nonrecurrent MECP2 duplications mediated by genomic architecture-driven DNA breaks and break-induced replication repair**. *Genome Research* 2008, **18**(6):847-858.
56. Froyen G, Belet S, Martinez F, Santos-Rebouças Cíntia B, Declercq M, Verbeeck J, Donckers L, Berland S, Mayo S, Rosello M *et al*: **Copy-Number Gains of HUWE1 Due to Replication- and Recombination-Based Rearrangements**. *American Journal of Human Genetics* 2012, **91**(2):252-264.
57. Fukami M, Tsuchiya T, Vollbach H, Brown KA, Abe S, Ohtsu S, Wabitsch M, Burger H, Simpson ER, Umezawa A *et al*: **Genomic Basis of Aromatase Excess Syndrome: Recombination- and Replication-Mediated Rearrangements Leading to CYP19A1 Overexpression**. *The Journal of Clinical Endocrinology and Metabolism* 2013, **98**(12):E2013-E2021.
58. Drier Y, Lawrence MS, Carter SL, Stewart C, Gabriel SB, Lander ES, Meyerson M, Beroukhim R, Getz G: **Somatic rearrangements across cancer reveal classes of samples with distinct patterns of DNA breakage and rearrangement-induced hypermutability**. *Genome Research* 2013, **23**(2):228-235.
59. Zhao P, Li J, Kang H, Wang H, Fan Z, Yin Z, Wang J, Zhang Q, Wang Z, Liu J-F: **Structural Variant Detection by Large-scale Sequencing Reveals New Evolutionary Evidence on Breed Divergence between Chinese and European Pigs**. 2016, **6**:18501.
60. Kiss T, Fayet-Lebaron E, Jády BE: **Box H/ACA Small Ribonucleoproteins**. *Molecular Cell* 2010, **37**(5):597-606.

61. Matsunaga S, Takata H, Morimoto A, Hayashihara K, Higashi T, Akatsuchi K, Mizusawa E, Yamakawa M, Ashida M, Matsunaga TM *et al*: **RBMX: a regulator for maintenance and centromeric protection of sister chromatid cohesion**. *Cell Rep* 2012, **1**(4):299-308.
62. Sun R-P, Xi Q-Y, Sun J-J, Cheng X, Zhu Y-L, Ye D-Z, Chen T, Wei L-M, Ye R-S, Jiang Q-Y *et al*: **In low protein diets, microRNA-19b regulates urea synthesis by targeting SIRT5**. *Scientific Reports* 2016, **6**:33291.
63. Wang T, Xu Z: **miR-27 promotes osteoblast differentiation by modulating Wnt signaling**. *Biochem Biophys Res Commun* 2010, **402**(2):186-189.
64. Clevers H, Loh KM, Nusse R: **Stem cell signaling. An integral program for tissue renewal and regeneration: Wnt signaling and stem cell control**. *Science* 2014, **346**(6205):1248012.
65. Ricklefs RE, Losos JB, Townsend TM: **Evolutionary diversification of clades of squamate reptiles**. *J Evol Biol* 2007, **20**(5):1751-1762.
66. van Hazel I, Sabouhanian A, Day L, Endler JA, Chang BSW: **Functional characterization of spectral tuning mechanisms in the great bowerbird short-wavelength sensitive visual pigment (SWS1), and the origins of UV/violet vision in passerines and parrots**. *BMC Evolutionary Biology* 2013, **13**:250-250.
67. de Lanuza GPi, Font E: **Ultraviolet vision in lacertid lizards: evidence from retinal structure, eye transmittance, SWS1 visual pigment genes and behaviour**. *The Journal of Experimental Biology* 2014, **217**(16):2899.
68. Laurent S, Pfeifer SP, Settles ML, Hunter SS, Hardwick KM, Ormond L, Sousa VC, Jensen JD, Rosenblum EB: **The population genomics of rapid adaptation: disentangling signatures of selection and demography in white sands lizards**. *Molecular Ecology* 2016, **25**(1):306-323.
69. Tobiishi M, Sayo T, Yoshida H, Kusaka A, Kawabata K, Sugiyama Y, Ishikawa O, Inoue S: **Changes in epidermal hyaluronan metabolism following UVB irradiation**. *Journal of Dermatological Science* 2011, **64**(1):31-38.
70. Urrutia R: **KRAB-containing zinc-finger repressor proteins**. *Genome Biol* 2003, **4**.
71. Wolfe SA, Nekludova L, Pabo CO: **DNA recognition by Cys2His2 zinc finger proteins**. *Annu Rev Biophys Biomol Struct* 2000, **29**.
72. Englbrecht CC, Schoof H, Böhm S: **Conservation, diversification and expansion of C2H2 zinc finger proteins in the Arabidopsis thaliana genome**. *BMC Genomics* 2004, **5**(1):39.
73. Emerson RO, Thomas JH: **Adaptive evolution in zinc finger transcription factors**. *PLoS Genet* 2009, **5**(1):e1000325.
74. Nowick K, Fields C, Gernat T, Caetano-Anolles D, Kholina N, Stubbs L: **Gain, loss and divergence in primate zinc-finger genes: a rich resource for evolution of gene regulatory differences between species**. *PLoS One* 2011, **6**(6):e21553.
75. Kolora SR, Faria R, Weigert A, Schaffer S, Grimm A, Henle K, Sahyoun AH, Stadler PF, Nowick K, Bleidorn C *et al*: **The complete mitochondrial genome of *Lacerta bilineata* and comparison with its closely related congener L. viridis**. *Mitochondrial DNA Part A* 2017, **28**(1):116-118.
76. Eyre-Walker A, Keightley PD, Smith NGC, Gaffney D: **Quantifying the Slightly Deleterious Mutation Model of Molecular Evolution**. *Molecular Biology and Evolution* 2000, **19**(12):2142-2149.
77. Charlesworth B: **Effective population size and patterns of molecular evolution and variation**. *Nat Rev Genet* 2009, **10**(3):195-205.
78. Maruyama T, Kimura M: **Genetic variability and effective population size when local extinction and recolonization of subpopulations are frequent**. *Proceedings of the National Academy of Sciences of the United States of America* 1980, **77**(11):6710-6714.
79. Sperveslage J, Hoffmeister M, Henopp T, Klöppel G, Sipos B: **Establishment of robust controls for the normalization of miRNA expression in neuroendocrine tumors of the ileum and pancreas**. *Endocrine* 2014, **46**(2):226-230.
80. Finn RM, Ellard K, Eirin-Lopez JM, Ausio J: **Vertebrate nucleoplasmin and NASP: egg histone storage proteins with multiple chaperone activities**. *FASEB J* 2012, **26**(12):4788-4804.

81. Nagatomo H, Kohri N, Akizawa H, Hoshino Y, Yamauchi N, Kono T, Takahashi M, Kawahara M: **Requirement for nuclear autoantigenic sperm protein mRNA expression in bovine preimplantation development**. *Anim Sci J* 2016, **87**(3):457-461.
82. Shang Y, Wang H, Jia P, Zhao H, Liu C, Liu W, Song Z, Xu Z, Yang L, Wang Y *et al*: **Autophagy regulates spermatid differentiation via degradation of PDLIM1**. *Autophagy* 2016, **12**(9):1575-1592.
83. Yin J, Ni B, Tian ZQ, Yang F, Liao WG, Gao YQ: **Regulatory effects of autophagy on spermatogenesis**. *Biol Reprod* 2017, **96**(3):525-530.
84. Chen J, Shishkin AA, Zhu X, Kadri S, Maza I, Guttman M, Hanna JH, Regev A, Garber M: **Evolutionary analysis across mammals reveals distinct classes of long non-coding RNAs**. *Genome Biol* 2016, **17**:19.
85. Wiberg RA, Halligan DL, Ness RW, Necsulea A, Kaessmann H, Keightley PD: **Assessing Recent Selection and Functionality at Long Noncoding RNA Loci in the Mouse Genome**. *Genome Biol Evol* 2015, **7**(8):2432-2444.
86. Zhen Y, Andolfatto P: **Methods to detect selection on noncoding DNA**. *Methods Mol Biol* 2012, **856**:141-159.
87. Bleiweiss R: **Ultraviolet plumage reflectance distinguishes sibling bird species**. *Proceedings of the National Academy of Sciences of the United States of America* 2004, **101**(47):16561.
88. Bajer K, Molnár O, Török J, Herczeg G: **Female European green lizards (Lacerta viridis) prefer males with high ultraviolet throat reflectance**. *Behavioral Ecology and Sociobiology* 2010, **64**(12):2007-2014.
89. Bajer K, Molnár O, Török J, Herczeg G: **Ultraviolet nuptial colour determines fight success in male European green lizards (Lacerta viridis)**. *Biology Letters* 2011, **7**(6):866-868.
90. Averbeck M, Gebhardt CA, Voigt S, Beilharz S, Anderegg U, Termeer CC, Sleeman JP, Simon JC: **Differential Regulation of Hyaluronan Metabolism in the Epidermal and Dermal Compartments of Human Skin by UVB Irradiation**. *Journal of Investigative Dermatology* 2007, **127**(3):687-697.
91. Kurdykowski S, Mine S, Bardey V, Danoux L, Jeanmaire C, Pauly G, Brabencova E, Wegrowski Y, Maquart FX: **Ultraviolet-B irradiation induces differential regulations of hyaluronidase expression and activity in normal human keratinocytes**. *Photochem Photobiol* 2011, **87**(5):1105-1112.
92. Elmayan T, Proux F, Vaucheret H: **Arabidopsis RPA2: a genetic link among transcriptional gene silencing, DNA repair, and DNA replication**. *Curr Biol* 2005, **15**(21):1919-1925.
93. Kim JS, Chae JH, Cheon YP, Kim CG: **Reciprocal localization of transcription factors YY1 and CP2c in spermatogonial stem cells and their putative roles during spermatogenesis**. *Acta Histochem* 2016, **118**(7):685-692.
94. Mengesdorf T, Proud CG, Mies G, Paschen W: **Mechanisms underlying suppression of protein synthesis induced by transient focal cerebral ischemia in mouse brain**. *Exp Neurol* 2002, **177**(2):538-546.
95. Sfakianos MK, Eisman A, Gourley SL, Bradley WD, Scheetz AJ, Settleman J, Taylor JR, Greer CA, Williamson A, Koleske AJ: **Inhibition of Rho via Arg and p190RhoGAP in the postnatal mouse hippocampus regulates dendritic spine maturation, synapse and dendrite stability, and behavior**. *J Neurosci* 2007, **27**(41):10982-10992.
96. Jacquet BV, Muthusamy N, Sommerville LJ, Xiao G, Liang H, Zhang Y, Holtzman MJ, Ghashghaei HT: **Specification of a Foxj1-dependent lineage in the forebrain is required for embryonic-to-postnatal transition of neurogenesis in the olfactory bulb**. *J Neurosci* 2011, **31**(25):9368-9382.
97. Huang Y, Xu Z, Cao J, Cao H, Zhang S: **The expression of FOXJ1 in neurogenesis after transient focal cerebral ischemia**. *Can J Neurol Sci* 2013, **40**(3):403-409.
98. Lu B, Gehrke S, Wu Z: **RNA metabolism in the pathogenesis of Parkinsons disease**. *Brain Res* 2014, **1584**:105-115.
99. Rieseberg LH, Burke JM: **A genic view of species integration**. *Journal of Evolutionary Biology* 2001, **14**(6):883-886.

100. Böhme MU, Schneeweiß N, Fritz U, Schlegel M, Berendonk TU: **Small edge populations at risk: genetic diversity of the green lizard (Lacerta viridis viridis) in Germany and implications for conservation management**. *Conservation Genetics* 2007, **8**(3):555-563.
101. Joger U, Fritz U, Guicking D, Kalyabina-Hauf S, Nagy ZT, Wink M: **Phylogeography of western Palaearctic reptiles – Spatial and temporal speciation patterns**. *Zoologischer Anzeiger - A Journal of Comparative Zoology* 2007, **246**(4):293-313.
102. Weigert A, Helm C, Meyer M, Nickel B, Arendt D, Hausdorf B, Santos SR, Halanych KM, Purschke G, Bleidorn C *et al*: **Illuminating the base of the annelid tree using transcriptomics**. *Mol Biol Evol* 2014, **31**(6):1391-1401.
103. Kircher M, Sawyer S, Meyer M: **Double indexing overcomes inaccuracies in multiplex sequencing on the Illumina platform**. *Nucleic Acids Res* 2012, **40**(1):e3.
104. Meyer M, Kircher M: **Illumina sequencing library preparation for highly multiplexed target capture and sequencing**. *Cold Spring Harb Protoc* 2010, **2010**(6):pdb prot5448.
105. Nawrocki EP, Eddy SR: **Infernal 1.1: 100-fold faster RNA homology searches**. *Bioinformatics* 2013, **29**(22):2933-2935.
106. Nawrocki EP, Burge SW, Bateman A, Daub J, Eberhardt RY, Eddy SR, Floden EW, Gardner PP, Jones TA, Tate J *et al*: **Rfam 12.0: updates to the RNA families database**. *Nucleic Acids Res* 2015, **43**(Database issue):D130-137.
107. Lowe TM, Eddy SR: **tRNAscan-SE: a program for improved detection of transfer RNA genes in genomic sequence**. *Nucleic Acids Res* 1997, **25**.
108. Bartschat S, Kehr S, Tafer H, Stadler PF, Hertel J: **snoStrip: a snoRNA annotation pipeline**. *Bioinformatics* 2014, **30**(1):115-116.
109. Jorjani H, Kehr S, Jedlinski DJ, Gumienny R, Hertel J, Stadler PF, Zavolan M, Gruber AR: **An updated human snoRNAome**. *Nucleic Acids Res* 2016, **44**(11):5068-5082.
110. Taft RJ, Glazov EA, Lassmann T, Hayashizaki Y, Carninci P, Mattick JS: **Small RNAs derived from snoRNAs**. *RNA* 2009, **15**(7):1233-1240.
111. Haas BJ, Papanicolaou A, Yassour M, Grabherr M, Blood PD, Bowden J, Couger MB, Eccles D, Li B, Lieber M *et al*: **De novo transcript sequence reconstruction from RNA-Seq: reference generation and analysis with Trinity**. *Nature protocols* 2013, **8**(8):10.1038/nprot.2013.1084.
112. Hertel J, Stadler PF: **The Expansion of Animal MicroRNA Families Revisited**. *Life (Basel)* 2015, **5**(1):905-920.
113. Smit A, Hubley R: **RepeatModeler Open-1.0**. 2008-2015.
114. Smit A, Hubley R, Green P: **RepeatMasker Open-4.0**. 2013-2015.
115. Li H: **Aligning sequence reads, clone sequences and assembly contigs with BWA-MEM**. *arXiv:13033997* 2013.
116. Garrison E, Marth G: **Haplotype-based variant detection from short-read sequencing**. *arXiv preprint* 2012, **arXiv:1207.3907**([q-bio.GN]*).
117. Lohse K, Harrison RJ, Barton NH: **A general method for calculating likelihoods under the coalescent process**. *Genetics* 2011, **189**(3):977-987.
118. Nurnberger B, Lohse K, Fijarczyk A, Szymura JM, Blaxter ML: **Para-allopatry in hybridizing fire-bellied toads (Bombina bombina and B. variegata): Inference from transcriptome-wide coalescence analyses**. *Evolution* 2016, **70**(8):1803-1818.
119. Tollis M, Ausubel G, Ghimire D, Boissinot S: **Multi-Locus Phylogeographic and Population Genetic Analysis of Anolis carolinensis: Historical Demography of a Genomic Model Species**. *PLoS One* 2012, **7**(6):e38474.
120. Caughley G: **Parameters for Seasonally Breeding Populations**. *Ecology* 1967, **48**(5):834-839.
121. Elbing K: **Zur Wiederbesiedlung brandgeschädigter Teilhabitate durch Smaragdeidechsen (Lacerta viridis)**. *Salamandra* 2000, **36**(3):175-184.
122. Saint Girons H, Castanet J, Bradshaw D, Baron J-P: **Démographie comparée de deux populations françaises de Lacerta viridis (Laurenti, 1768)**. *Journal of Ecology* 1989, **44**(4):361-386.
123. Rykena S, Nettmann HK: **Lacerta viridis (Laurenti, 1768) – Smaragdeidechse**, vol. 2; 1984.

124. Mohiyuddin M, Mu JC, Li J, Bani Asadi N, Gerstein MB, Abyzov A, Wong WH, Lam HY: **MetaSV: an accurate and integrative structural-variant caller for next generation sequencing**. *Bioinformatics* 2015, **31**(16):2741-2744.

125. Chen K, Wallis JW, McLellan MD, Larson DE, Kalicki JM, Pohl CS, McGrath SD, Wendl MC, Zhang Q, Locke DP *et al*: **BreakDancer: an algorithm for high-resolution mapping of genomic structural variation**. *Nat Methods* 2009, **6**(9):677-681.

126. Abyzov A, Urban AE, Snyder M, Gerstein M: **CNVnator: an approach to discover, genotype, and characterize typical and atypical CNVs from family and population genome sequencing**. *Genome Res* 2011, **21**(6):974-984.

127. Ye K, Schulz MH, Long Q, Apweiler R, Ning Z: **Pindel: a pattern growth approach to detect break points of large deletions and medium sized insertions from paired-end short reads**. *Bioinformatics* 2009, **25**(21):2865-2871.

128. Xie C, Tammi MT: **CNV-seq, a new method to detect copy number variation using high-throughput sequencing**. *BMC Bioinformatics* 2009, **10**:80-80.

129. Kent WJ, Baertsch R, Hinrichs A, Miller W, Haussler D: **Evolution's cauldron: duplication, deletion, and rearrangement in the mouse and human genomes**. *Proc Natl Acad Sci U S A* 2003, **100**(20):11484-11489.

130. Blanchette M, Kent WJ, Riemer C, Elnitski L, Smit AF, Roskin KM, Baertsch R, Rosenbloom K, Clawson H, Green ED *et al*: **Aligning multiple genomic sequences with the threaded blockset aligner**. *Genome Res* 2004, **14**(4):708-715.

131. Hickey G, Paten B, Earl D, Zerbino D, Haussler D: **HAL: a hierarchical format for storing and analyzing multiple genome alignments**. *Bioinformatics* 2013, **29**(10):1341-1342.

132. Harris R: **Improved pairwise alignment of genomic DNA.** . *Ph.D. Thesis.* The Pennsylvania State University.; 2007.

133. Walter Costa MB, Höner zu Siederdissen C, Stadler PF, Nowick K: **SSS-test: a novel test for detecting selection on the secondary structures of non-coding RNAs. (submitted)**. *Nucleic Acids Res* 2017.

134. Lorenz R, Bernhart SH, Höner zu Siederdissen C, Tafer H, Flamm C, Stadler PF, Hofacker IL: **ViennaRNA Package 2.0**. *Algorithms for Molecular Biology* 2011, **6**(1):26.

135. Lange SJ, Maticzka D, Mohl M, Gagnon JN, Brown CM, Backofen R: **Global or local? Predicting secondary structure and accessibility in mRNAs**. *Nucleic Acids Res* 2012, **40**(12):5215-5226.

136. Yates A, Akanni W, Amode MR, Barrell D, Billis K, Carvalho-Silva D, Cummins C, Clapham P, Fitzgerald S, Gil L *et al*: **Ensembl 2016**. *Nucleic Acids Res* 2016, **44**(D1):D710-716.

137. Hongo JA, de Castro GM, Cintra LC, Zerlotini A, Lobo FP: **POTION: an end-to-end pipeline for positive Darwinian selection detection in genome-scale data through phylogenetic comparison of protein-coding genes**. *BMC Genomics* 2015, **16**:567.

138. Yang Z: **PAML 4: phylogenetic analysis by maximum likelihood**. *Mol Biol Evol* 2007, **24**(8):1586-1591.

Figure Legends

Figure 1. Demographic models for the divergence between *L. viridis* and *L. bilineata*. Divergence at time T with different N_e in one group (M2.1, M2.2) and divergence at time T with continuous unidirectional gene flow with different N_e in one group (M4.1, M4.2). The grey area indicates gene flow between both species and the similar shading of the branches indicates similar effective population sizes (N_e). $N_{A,B}$ - effective population size compared to ancestor, T - split time.

Figure 2. Total counts and length ranges (in bp) of genomic rearrangements of SVs between L. viridis and L. bilineata. The counts are represented by bars and length ranges by whiskers (y-axis is log10-scaled). The rearrangements plotted are categorized into deletions (DEL), duplications (DUP), insertions (INS) and inversions (INV).

Figure 3. The folded blockwise site frequency spectrum (bSFS). The variation in alleles represented by different colours (the ancestral state showed in red). Given a single genealogy (a diploid genome from two populations can form six possible genealogies), each block contains four mutation types: i) unique heterozygous sites in L. bilineata, ii) unique heterozygous sites in L. viridis, iii) shared heterozygous sites between L. viridis and L. bilineata or iv) homozygous sites which are different between L. viridis and L. bilineata i.e. homozygous fixed differences. The bSFS (spectrum of SFS) has been calculated by counting the number of occurrences of each SFS.

Tables

Table 1. Comparison of different demographic models for divergence between *L. viridis* and *L. bilineata*. The Δ in log likelihood of each model is given relative to the best model. V - *L. viridis*, B - *L. bilineata*. '→' indicates the direction of gene flow between the two species; 2Ne - two different effective population sizes assumed; IM - isolation with migration; DIV_V - divergence without gene flow assuming dissimilar effective population size in *L. viridis* compared to the lacertid ancestor and *L. bilineata*; DIV_B - divergence without gene flow assuming dissimilar effective population size in L. bilineata compared to the lacertid ancestor and *L. viridis*.

**M2 (without gene flow)**		**M4 (presence of gene flow)**	
**DIV2NeV**	**DIV2NeB**	**IM2NeV→B**	**IM2NeB→V**
-9.44	-9.45	0	-3.04
-12.1	-12.0	0	-1.27
-16.9	-16.7	0	-1.16

Supplementary File 1

Click here to access/download
Supplementary Material
Lacertids_Manuscript-Gigascience_Supplement.pdf

Supplementary File 2

Click here to access/download
Supplementary Material
Additional_File_2.pdf

Figure 1

Click here to access/download
Supplementary Material
Figure_1.svg

Figure 2

Click here to access/download
Supplementary Material
Figure_2.svg

Figure 3

Click here to access/download
Supplementary Material
Figure_3.svg

Click here to access/download
Supplementary Material
Additional_file_3.nb

Mathematica Notebook 2

Click here to access/download

Supplementary Material

Additional_file_4.nb
