# Supplementary material for: Divergent evolution in the genomes of closely related lacertids, Lacerta viridis and L. bilineata, and implications for speciation
Source: Gigascience. 2018 Dec 10;8(2):giy160. doi: 10.1093/gigascience/giy160 (PMC6381762; doi:10.1093/gigascience/giy160)
Supplement: GIGA-D-18-00173_Revision_1.pdf [file giy160_giga-d-18-00173_revision_1.pdf]

GigaScience

Divergent evolution in the genomes of closely-related lacertids, Lacerta viridis and L. bilineata and implications for speciation

--Manuscript Draft--

**Manuscript Number:**	GIGA-D-18-00173R1	
**Full Title:**	Divergent evolution in the genomes of closely-related lacertids, Lacerta viridis and L. bilineata and implications for speciation	
**Article Type:**	Research	
**Funding Information:**	Deutsche Forschungsgemeinschaft (FZT118)	Dr. Martin Schlegel
**Abstract:**	Background: Lacerta viridis and L. bilineata are sister species of European green lizards (eastern and western clades respectively), which until recently were grouped together as the L. viridis complex. Genetic incompatibilities were observed between lacertid populations through crossing experiments, which led to the delineation of two separate species within the L. viridis complex. The population history of these sister species and processes driving divergence are unknown. We constructed high quality de novo genome assemblies for both L. viridis and L. bilineata through Illumina and PacBio sequencing, with annotation support provided from transcriptome sequencing of several tissues. To estimate gene flow between the two species and identify factors involved in reproductive isolation, we studied their evolutionary history, identified genomic rearrangements, detected signature of selection on non-coding RNA and protein-coding genes.<br>Findings: Here we show that the gene flow was primarily unidirectional from L. bilineata to L. viridis after their split at least 1.15 Mya. We detected positive selection of the non-coding repertoire; mutations in transcription factors; accumulation of divergence through inversions; selection on genes involved in neural development, reproduction and behavior, as well as in UV-response possibly driven by sexual selection, whose contribution to reproductive isolation between these lacertid species need to be further evaluated.<br>Conclusion: The combination of short and long sequence reads resulted in one of the most complete lizard genome assemblies. The characterization of a diverse array of genomic features provided valuable insights into the demographic history of divergence among European green lizards, as well as key species differences, some of which are candidates that could have played a role in speciation. In addition, our study generated valuable genomic resources that can be used to address conservation related issue in lacertids.	
**Corresponding Author:**	Sree Rohit Raj Kolora<br><br>GERMANY	
**Corresponding Author Secondary Information:**		
**Corresponding Author's Institution:**		
**Corresponding Author's Secondary Institution:**		
**First Author:**	Sree Rohit Rohit Kolora	
**First Author Secondary Information:**		
**Order of Authors:**	Sree Rohit Rohit Kolora	
	Anne Weigert	
	Amin Saffari	
	Stephanie Kehr	
	Maria Beatriz Walter Costa	
	Cathrin Spröer	
	Henrike Indrischek	

Powered by Editorial Manager® and ProduXion Manager® from Aries Systems Corporation

	Gero Doose
	Manjusha Chintalapati
	Konrad Lohse
	Jörg Overmann
	Boyke Bunk
	Christoph Bleidorn
	Klaus Henle
	Katja Nowick
	Rui Faria
	Peter F Stadler
	Martin Schlegel
**Order of Authors Secondary Information:**	
**Response to Reviewers:**	Dear Dr. Scott Edmunds, Executive Editor of GigaScience:<br><br>We would like to resubmit the revised manuscript entitled "Divergent evolution in the genomes of closely-related lacertids, Lacerta viridis and L. bilineata and implications for speciation", previously submitted to GigaScience (GIGA-D-18-00173). We believe we have addressed all the reviewers' helpful and constructive points, performed the necessary modifications and corrections with additional analysis. We would like to thank the reviewers for their time invested in reviewing our manuscript. Below, we present detailed responses to all comments, hoping that the current version meets the quality standards for being published in GigaScience.<br><br>Sincerely,<br>Sree Rohit Raj Kolora, on behalf of all authors<br><br>Reviewer suggestions:<br><br>Reviewer #1: Kolora and colleagues assemble the genomes of two sister species of green lizards to determine the basis of the genomic incompatibility between them. The study is quite explorative and descriptive, testing a whole bunch of stuff that turned out to be of interest in other taxa. The MS is generally well-written but there are some textual issues listed below.<br>In general I find this study lacking a clear focus. In lines 116-120 a clear aim of this study is missing. You do a whole bunch of analyses and the MS is quite long-winded, but the common thread is missing or at least obscured. The elements are all there though, so I would like to ask you to make this a bit tighter and to the point. The green lizard hybrid zone is a great system so leverage that in your advantage.<br>To address this we have rewritten parts of our introduction and discussion to clarify the main aim of the study which is: "identifying candidates and traits involved in divergent evolution of sister species" – namely UV-reflective differences influencing sexual selection, structural divergence in ncRNAs, association of inversions with PSGs, adaptive differences in transcription factors and genes related to brain development and behavior.<br>The green lizard hybrid zone though interesting is hard to define due to the presence of multiple contact zones (Marzahn et. al. 2016). The demography of the hybrid zones is still not completely clear and this can only be resolved by sequencing the individuals from this region, for which our study provides the reference genomes to compare.<br><br>Lines 55-61: I find this section confusing. Your point ii simply seems a restatement of what you want to argue. From point iii it does not follow why this should prevent species merger, because could this not actually drive interspecific introgression?<br>This part has been corrected to remove portions that have not been described in the manuscript. The basic notion was to explain how inversions can contribute to

divergence between species despite ongoing gene flow or admixture. We tried to clarify the third point to avoid confusion.

Lines 71-76: For example, this is all very general and what is lacking is a clear reasoning why you are doing this analysis for the green lizards in particular.
Our apologies for the confusion, this and the previous paragraph are intended to describe the current methods that we can employ to get information from the genomes of two closely related species about their divergence process, including their demographic history (in this particular paragraph). The system of green lizards is introduced in the following paragraph and their features of interest are described.

Line 338-344: Your argument here is not clear (to me). The Ne of Lbil is larger than Lvir because of more structure in Lbil but then you appear to be talking about a subpopulation of Lvir (eastern clade) so that suggests Lvir has plenty of substructure?
This objection is correct and we performed an extensive analysis with more blocks data. This supports Lvir to have a higher Ne compared to Lbil with gene flow from Lbil to Lvir. This part has been rewritten with this additional analysis which includes the model for admixture.

Textual:
Line 46: The "non-essential" is a bit misleading of course, it depends on what perspective (something similar in abstract too).
This has been removed.

Line 105: The "Furthermore" does not follow, it is a disadvantage of the mtDNA/nuDNA sequence study over allozymes that you highlight here. Your aim is to show that there are still uncertainties.
This has been modified accordingly.

Line 109 and 111: Two times "This" is confusing. I don't see how it follows here that genomic re-arrangements are likely.
“This” has been removed and the sentences rewritten, genomic rearrangements were hypothesized earlier between lacertids due to changes in chromosome morphology (citation added).

Line 333-334: "which are 95% identical", these words are unnecessary and make for a confusing sentence (identical to what exactly?).
The sentence has been corrected.

Line 362: word "is" missing
This has been corrected.

Line 398: word "with" redundant
This has been corrected.

Reviewer #2: In the manuscript "Divergent evolution in the genomes of closely-related lacertids, Lacerta viridis and L. bilineata and implications for speciation" Kolora and colleagues present de novo genome assemblies of two species of green lizards based on a combination of Illumina short reads and PacBio sequencing. This resulted in a pair of impressively contiguous and complete genomes which they annotate using a set of tissue-specific transcriptome sequencing. The authors go on to perform an exhaustive array of analyses to characterize structural, copy number, and sequence divergence between these two species, the ancestral lineage of these two species, and a selection of additional existing genomes. While I feel this work has the potential to be a great contribution, there are number of issues (detailed below) that I feel must first be addressed.

Line 18: "…identify intrinsic and extrinsic factors involved in reproductive isolation"
While the divergence between these species is well characterized in this manuscript, the specific factors involved in reproductive isolation are not identified. The authors do suggest a number of viable hypotheses regarding the basis of isolation. Indeed, the authors discuss in their introduction how speciation genes have largely been identified in model organisms due to the tractability of genetic crosses. These speciation genes

	are specifically those show to play a direct role in reproductive isolation and not all genes that have experienced divergence or selection between lineages. I would consider revising this sentence and elsewhere to reflect this distinction.<br>The factors for reproductive isolation have been mentioned in lines 303, 370, 383, 397-398. Additional corrections were made in parts of discussion and in the conclusion to include these factors.<br><br>Line 25: "…have been critical for reduced reproductive success". Same comment as above. Considering adding "may" to the start of this phrase.<br>This has been corrected.<br><br>Line 49: "diversification" Perhaps the authors mean divergence or speciation?<br>“diversification” replaced by “divergence eventually leading to speciation”<br><br>Line 52: "allowing to test" This sentence is missing a subject.<br>Subject “us” has been added.<br><br>Line 53: A comma is needed after "Specifically".<br>This has been corrected.<br><br>Line 65: First mention of KZNFs here without clear explanation of why they are singled out.<br>KZNFs have been removed from the sentence. The rationale was that KZNFs are known to be involved in species differences between primates, this has been referred to in the discussion.<br><br>Line 98: "Corresponding to lineage B" please add detail here on the citation of lineage B and V.<br>The detailed citations have been added.<br><br>Results: Please report the total size of each assembly in the text.<br>This information has been added.<br><br>Line 129: The Anolis carolinensis genome was not generated via high-coverage Illumina sequencing.<br>This has been corrected.<br><br>Line 129 (and Table S1): Also, while it is accurate that contig N50 in the current study exceed contig N50 for Anolis carolinensis, the scaffold N50 for the carolinensis genome is multiple orders of magnitude more contiguous (151 Mb - note this has been often misreported at 4Mb in the literature, see Tollis et al 2018 for correct scaffold N50). I suggest making this point clear.<br>This has been corrected in the supplement. An additional sentence on higher N50 of anolis genome was added.<br><br>Line 147: The reported karyotype is that of Anolis carolinensis, but is not shared by all anoles. Perhaps rephrase?<br>A. carolinensis has been specifically mentioned.<br><br>Line 151: Please provide a citation for a divergence time of 150Mya or more.<br>Citation has been provided.<br><br>Line 186: This estimate of Fst should be considered a maximum as using heterozygosity from single individuals will have the effect of maximizing the estimate of Dxy and minimizes estimates of pi. I suggest making it clear that actual Fst is likely lower.<br>The heterozygosity of an individual is an unbiased estimate of pairwise pi (given a panmictic population) and d_xy is similarly sample size independent. Hence we would like to kindly inform that our estimate of F_st is indeed unbiased and not some upper lower bound.<br><br>Line 187, 203, 489: While I commend the authors for including their Mathematica code as a supplement, I think addition detail on this method and results should be provided. What are the likelihoods of each model? How much better than other models was

	model M4.1? How were more complex models compared to models with fewer parameters?<br>This was previously present in the supplement. We have moved it to the main text including additional analysis with an admixture model.<br><br>Line 254: The maintenance of opsin paralogs over long periods of evolutionary times certainly suggest they are functionally important, but the phrase "similar diurnal adaptations" suggests parallel evolution, where the more likely scenario (I think) is simply conservation.<br>We completely agree and did not intend to imply parallel evolution. We have corrected this sentence to imply conservation.<br><br>Line 305: "Neutrally evolving genes" Please be specific about how genes are classified as "neutrally evolving". Does this mean they are unconstrained or that there is no evidence of positive selection in branch-site tests?<br>We have corrected this to imply genes with no signs of positive selection.<br><br>Line 311: "indels over-showing" I'm not clear what is meant by "over-showing". Please consider rephrasing?<br>This has been corrected with “obscuring”.<br><br>Line 316: How were multiple Boschloo exact-tests tests corrected?<br>FDRs were calculated and the values have been added.<br><br>Line 337: "Therefore, our results support the separate species status of L. viridis and L. bilineata." Which species concept is used to underpin this statement?<br>We rewrote this statement more carefully, since we do not want to indulge in any discussion of the numerous and controversial species concepts here.<br><br>Line 338: "Confirmed" The demographic modeling supported unidirectional gene flow, but confirmed seems too strong of a sentiment here.<br>Agreed, this has been replaced with “supports”.<br><br>Line 340: While population subdivision can increase heterozygosity and estimates of effective population size via the Wahlund effect, the heterozygosity of a single individual (a member of just a single subpopulation) would not reflect this increase heterozygosity. I would suggest the authors consider other possible explanations. For example, their hypothesis of unidirectional gene flow into L. bilineata could, by itself, explain elevated heterozygosity if the sequenced individual contains L. viridis alleles due to introgression.<br>We have re-analysed the demographic patterns with additional data and more extensive models including simple divergence, isolation with migration and admixture. In this improved analyses, the best model supports gene flow predominantly from L. bilineata to L. viridis. Since gene flow occurs in this direction, L. viridis could have a higher effective population size.<br><br>Line 508: "on overage" Typo?<br>This has been corrected.<br><br>Line 509: "… this data" should be "… these data"<br>This has been corrected.<br><br>Line 640: Please cite any relevant animal use and care authorizations.<br>The capture permits were added in the acknowledgements, and the animal care for experimentation has been specified in the methods. The permits can be provided to the editor upon request. We do not have a specific ethics committee at our institution, hence we followed the guidelines provided by the Herpetological Animal Care and Use Committee (HACC) of the American Society of Ichthyologists and Herpetologists.
**Additional Information:**	
**Question**	**Response**
Are you submitting this manuscript to a special series or article collection?	No

**Experimental design and statistics**<br><br>Full details of the experimental design and statistical methods used should be given in the Methods section, as detailed in our Minimum Standards Reporting Checklist. Information essential to interpreting the data presented should be made available in the figure legends.<br><br>Have you included all the information requested in your manuscript?	Yes
**Resources**<br><br>A description of all resources used, including antibodies, cell lines, animals and software tools, with enough information to allow them to be uniquely identified, should be included in the Methods section. Authors are strongly encouraged to cite Research Resource Identifiers (RRIDs) for antibodies, model organisms and tools, where possible.<br><br>Have you included the information requested as detailed in our Minimum Standards Reporting Checklist?	Yes
**Availability of data and materials**<br><br>All datasets and code on which the conclusions of the paper rely must be either included in your submission or deposited in publicly available repositories (where available and ethically appropriate), referencing such data using a unique identifier in the references and in the “Availability of Data and Materials” section of your manuscript.<br><br>Have you have met the above requirement as detailed in our Minimum Standards Reporting Checklist?	Yes

Powered by Editorial Manager® and ProduXion Manager® from Aries Systems Corporation

Manuscript

Click here to view linked References

Click here to access/download;Manuscript;Lacertids_Manuscript-

Divergent evolution in the genomes of closely-related lacertids, *Lacerta viridis* and *L. bilineata* and implications for speciation

Rohit Kolora, Anne Weigert[¥], Amin Saffari[¥], Stephanie Kehr, Maria Beatriz Walter Costa, Cathrin Spröer, Henrike Indrischek, Gero Doose, Manjusha Chintalapati, Konrad Lohse, Jörg Overmann, Boyke Bunk, Christoph Bleidorn, Klaus Henle, Katja Nowick, Rui Faria, Peter F Stadler, Martin Schlegel

[¥] These authors contributed equally to this work.

Abstract

Background: *Lacerta viridis* and *L. bilineata* are sister species of European green lizards (eastern and western clades respectively), which until recently were grouped together as the *L. viridis* complex. Genetic incompatibilities were observed between lacertid populations through crossing experiments, which led to the delineation of two separate species within the *L. viridis* complex. The population history of these sister species and processes driving divergence are unknown. We constructed high quality *de novo* genome assemblies for both *L. viridis* and *L. bilineata* through Illumina and PacBio sequencing, with annotation support provided from transcriptome sequencing of several tissues. To estimate gene flow between the two species and identify factors involved in reproductive isolation, we studied their evolutionary history, identified genomic rearrangements, detected signature of selection on non-coding RNA and protein-coding genes.

Findings: Here we show that the gene flow was primarily unidirectional from *L. bilineata to L. viridis* after their split at least 1.15 Mya. We detected positive selection of the non-coding repertoire; mutations in transcription factors; accumulation of divergence through inversions; selection on genes involved in neural development, reproduction and behavior, as well as in

UV-response possibly driven by sexual selection, whose contribution to reproductive isolation between these lacertid species need to be further evaluated.

Conclusion: The combination of short and long sequence reads resulted in one of the most complete lizard genome assemblies. The characterization of a diverse array of genomic features provided valuable insights into the demographic history of divergence among European green lizards, as well as key species differences, some of which are candidates that could have played a role in speciation. In addition, our study generated valuable genomic resources that can be used to address conservation related issue in lacertids.

Introduction

Understanding what species are and the processes driving their emergence have been two central issues in biology [1]. During the last century, genes involved in reproductive isolation were mainly identified in model organisms, such as *Drosophila* [2]. These studies aiming at the so-called "speciation genes" revealed at least three general patterns: i) genes involved in post-zygotic incompatibilities show signatures of accelerated evolution [2]; ii) incompatibilities often involve a disproportionate number of genes located on sex chromosomes [3, 4] and iii) mis-expression is often observed in hybrids, suggesting that gene regulation is an important component of speciation [5-7]. However, the identification of incompatibilities using laborious lab crosses was only possible for model organisms, and thus the identification of loci involved in reproductive isolation [8] in natural populations remained largely unknown.

The advent of high throughput sequencing together with the development of novel approaches for whole genome analyses opened new research avenues to study the origin of species, including non-model organisms [9]. It has been shown that genes involved in adaptation and speciation are often found in regions of low recombination, such as genomic rearrangements,

suggesting that they play an important role in species diversification [10]. Several *in-silico* tools have been developed to detect structural variation with high precision using genomic data [11-13], thus enabling us to test evolutionary hypotheses such as the role of genomic rearrangements in speciation over a wider taxonomic range [14].

The assessment of divergence in regulatory elements and transcription factors between species further adds to a more complete understanding of the link between genotypes and phenotypes. In this respect, transcriptome sequencing offers an unprecedented resolution to investigate the general importance of divergence in gene regulation in speciation. In particular, zinc-finger genes, especially *Krüppel*-type zinc fingers (KZNFs), a family of transcription factors were pinpointed as strong candidates to play a role in the speciation of other vertebrates [6]. In addition, various epigenetic mechanisms between species mediated by non-coding RNA (ncRNA) can also contribute to speciation [15-18].

Nevertheless, our understanding of how speciation unfolds, as well as the mechanisms involved will remain limited without the knowledge of the demographic history between diverging taxa [8]. Model-based methods are now available to infer the demographic history of recently diverged taxa based on genome data from a few individuals of each species [19]. Thus, patterns of gene flow and population size changes during divergence can now be inferred without extensive sampling [20].

In summary, the identification of differences in genomic features between closely related species and their demographic history can now be assessed in a cost-effective manner. The resulting information is likely to provide insights about the main candidates playing a role in diversification, upon which more specific hypotheses concerning the mechanisms of divergence can be tested.

Lizards provide an excellent model for studying speciation due to the existing knowledge on their long-term demographics and adaptive morphologies, in addition to the ease of sample

collection and experimental manipulations [21]. Lizards of the genus *Anolis*, in particular, have been studied in detail, as their distribution on islands coupled with repeated adaptive radiations offer a perfect framework for evolutionary ecology studies [22]. Not surprisingly, the first sequenced squamate genome was an anole lizard [23]. Comparative genomic analysis of *Anolis carolinensis* (anole lizard) with the genomes of birds and mammals was pivotal in identifying accelerated evolution of egg proteins associated with amniote evolution [23]. Further sauropsid genomes (birds and reptiles) were sequenced in recent years, now covering a broader taxonomic range of Squamata, Archosauria and Chelonia [24-31]. For instance, the study of *Gekko japonicus* (gecko lizard) contributed to the understanding of evolution and adaptation of tail regeneration, clinging, nocturnal vision and diversification of the olfactory system [26]. In addition, the genomes of *Pogona vitticeps* (bearded dragon lizard) and *Shinisaurus crocodilurus* (Chinese crocodile lizard) have recently been characterized [24, 27]. However, comparative genome analyses of closely-related lizard species pairs have been limited to anoles, where adaptive evolution of genes related to brain development and behaviour was recently reported [32].

The family Lacertidae (Sauropsida, Squamata) has been well covered in-terms of phylogeographic studies, providing important information about the likely timing and geographic context of speciation [21]. Within this family, the *Lacerta viridis* complex shows an intricate evolutionary history with secondary contact zones [33, 34]. Here, we focus on the divergence between the western clade formally described as *L. bilineata* and the eastern clade of *L. viridis* (corresponding to lineage B and lineage V respectively of Marzahn *et al.*) that currently occupy disjoint regions in Europe [34].

Adult individuals from the two taxa are very similar: throat colouration of hatchlings and early juveniles is the only described diagnostic trait so far [35]. Gene flow between these two species was previously hypothesized in studies of allozyme variation [36, 37]. However, recent

analyses based on mtDNA and one nuclear marker (fibint7) have cast doubt on the taxonomic classification of the individuals analyzed in those studies and did not provide conclusive evidence either for or against gene flow between *L. viridis* and *L. bilineata* [34].

Hybrids between different main lineages within the *L. viridis* complex (northern Italy and Hungary) exhibit reduced fitness under laboratory conditions [38]. This suggests that at least partial reproductive isolation between *L. viridis* and *L. bilineata* can exist in the wild due to genomic Bateson-Dobzhansky-Muller incompatibilities (BDMIs). Previous models have suggested that after a secondary contact, BDMIs can be maintained and further accumulate within genome rearrangements [39, 40], thus avoiding species fusion [41]. High karyotypic variability has been observed in reptiles [42], also within the *L. viridis* complex [43], raising the prospect that genomic rearrangements could also be involved in their diversification [44]. Finally, lizard-specific KZNF genes have recently been predicted [45], making our focal pair of taxa an excellent case study of evolution in this class of genes and their role in speciation via changes in gene regulatory networks. Overall, the *L. viridis* complex comprises a very interesting system where different genomic components can be studied to elucidate the demographic history and possible processes involved in speciation.

Here, we combine short Illumina and long PacBio read sequencing approaches to construct high quality *de novo* genomes for both *L. bilineata* and *L. viridis*, with annotation support from transcriptomic data. We investigated the demographic history of divergence between the two lacertid taxa and performed a broad comparison of key genomic features providing important insights about their divergence that can be tested in future studies aiming to identify the mechanisms ultimately leading to speciation between this closely related species pair.

Results

The genomes of *L. viridis* and *L. bilineata*

We employed a hybrid strategy of combining Illumina and PacBio sequencing data to produce separate genome assemblies for the two lacertid species. Genome sequencing coverages of 34x Illumina and 14x PacBio for *L. viridis*; and 37x Illumina and 11x PacBio for *L. bilineata* aided in the construction of high quality genome assemblies (supplement SI-1; Figure S2, S3). The genome assembly sizes were 1.44 Gbp and 1.42 Gbp for *L. viridis* and *L. bilineata* respectively. The assembled lacertid genomes achieved better contiguity than the high coverage illumina-only contigs of *G. japonicus* but lower than the chromosome level assembly of *A. carolinensis* (368 kbp and 663 kbp for *L. bilineata* and *L. viridis* respectively versus 150 Mbp in *A. carolinensis*) (Table S1). While the BUSCO (Benchmarking Universal Single-Copy Orthologs) completeness in terms of single-copy ortholog (SCO) genes with vertebrate core gene set were 96% and 94% respectively, higher than in the available lizard genomes. Since the genome of *L. viridis* had better contiguity than *L. bilineata* (higher N50 and lesser number of contigs), *L. viridis* was used as the reference to predict genomic variants (structural variants (SVs) and single nucleotide polymorphisms (SNPs) between the two taxa.

Lacerta viridis genome consisted of higher number of large segmental duplications (>5 kbp) than *L. bilineata* (Figure S4). However, since no significant differences were observed in segmental duplications (>1 kbp) between the two lacertid genomes (F-test: p=0.35 and Wilcoxon test: p=0.55). Hence, the occurrence of lesser large segmental duplications in *L. bilineata* could be a result of higher fragmentation in its genome assembly. Synteny information was used to create unordered contig clusters (min. size of 1 Mbp covering one-third of the *L. viridis* genome) which roughly represent positioning on the same chromosome (Suppl. File S2). The median synonymous substitution rate (Ks) and non-synonymous substitution rate (Ka)

based on 7,030 SCOs between the two lacertid species were 0.021 and 0.016 respectively. A mutation rate of 1 x 10^{-9} substitutions per site per generation was estimated from the fourfold degenerate sites. This mutation rate observed in the ancestral lacertid lineage is similar to the ancestral bird lineage (1.15-1.23 x 10^{-9} per site per generation) [46, 47].

The identical structures of the HOX-cluster between the lacertid species and *A. carolinensis* confirmed the high genomic assembly quality since the HOX-clusters are highly conserved (supplement SI-1). The number of chromosomes and the sex-determination system are different between *A. carolinensis* (2n=36, 12 macro- and 24 microchromosomes; XY) and lacertid lizards (2n=38; 36 macro- and 2 microchromosomes; ZW) [43, 48]. However, genomic contigs of both lacertid species were syntenic without breaks or inter-chromosomal transpositions to the macro-chromosomes of *A. carolinensis* (Figure S2), even though the lacertids and anoles split more than 150 Mya [49]. The only exception to this was a *L. viridis* contig that splits into two macro-chromosomes of the *A. carolinensis* genome. This particular contig of *L. viridis* was syntenic to five separate contigs in *L. bilineata* assembly, confirming a higher fragmentation in genome assembly of the latter.

The assembled transcripts were crucial for gene annotations since the *ab initio* methods predicted more fragmented proteins and coding sequences (CDS) (38,000-55,000) when compared to the final gene models (22,100-22,500) (Table S2). A majority of the longest *de novo* assembled transcript isoforms were from the ovarian tissue followed by the brain. Since the sequencing throughput was highest for the liver tissue in both species, this finding was not likely the result of sequencing artifacts. We identified 22,156 genes in *L. viridis* and 22,491 genes in *L. bilineata* supported by *de novo* assembled transcripts (supplement SI-2; Table S2). The higher number of genes in *L. bilineata* was due to the fragmentation of a few genes onto multiple contigs, which can be resolved in the future with scaffolding information. Compared to *A. carolinensis*, we observed an over-representation of genes involved in transfer RNA

(tRNA) aminoacylation (Panther release 20170413, fold-enrichment=2.13-2.25, $p<0.03$) and tRNA metabolic process (Panther release 20170413, fold-enrichment=1.84-1.89, $p<0.003$) in both lacertids, indicating an expansion of tRNA-processing genes before their split. Putative Z-chromosome linked contigs consisted of few non-coding elements (7-11 microRNAs, 1 snoRNA, 2-3 snRNAs and 46-53 functional tRNAs) (supplement SI-3). The total length of the contigs assigned to the Z-chromosome in lacertids was larger (13.5-15.6 Mbp) than the Z-chromosomes of *P. vitticeps* (8 Mbp) but the number of identified genes were similar (205-221 and 219, respectively) [50].

The number of predicted members of the different non-coding RNA classes was similar in *L. viridis* and *L. bilineata* (Table S3). Compared to other selected tetrapod species, there was an increase in the number of tRNAs (both functional- and pseudo-tRNAs) in the two lacertid species (Figure S5, S6). However, the numbers of tRNAs and pseudo-tRNAs are known to vary significantly in eukaryotes[51]. We found an over-representation of tRNA-processing genes supported by the expansion of tRNA elements in both lacertid species maintained through deletion-duplication events. microRNAs and snoRNAs in the lacertids exhibited losses compared to *A. carolinensis* (Figure S7, S8). Even though the numbers of snoRNAs and miRNAs were almost identical, the members in each ncRNA class diversified between the two sister species. Repeat content also differed between *L. viridis* and *L. bilineata*; with the latter exhibiting a gain of long-terminal repeat (LTR) elements (Table S5).

Demographic history of divergence

Across all sites, mean heterozygosity was slightly lower in *L. bilineata* than in *L. viridis* (pi=0.0022 and 0.0029 respectively). Absolute divergence per site between the two species as measured by D_{xy} was around 0.0123. A maximum pairwise F_{ST} of 0.688 was estimated between *L. viridis* and *L. bilineata*.

We inferred past divergence and gene flow between the two lacertid species using a likelihood method based on the site frequency spectrum of short sequence blocks i.e. blockwise site frequency spectrum (bSFS) [20, 52]. Since the likelihood calculation assumes no recombination within blocks and an infinite sites mutation model, we partitioned the genome into short (i.e. 200 bases) blocks. Our dataset consisted of 5,654,020 blocks, of which 46,825 were filtered out (0.83%) since they contained both fixed differences and shared heterozygous sites thus violating the 4-gametes criterion under the assumption of no recombination within blocks.

In total, we have 2785 distinct mutational configurations i.e. the counts of the four entries of the folded joint site frequency spectrum (heterozygous sites unique to *L. viridis* and *L. bilineata*; heterozygous sites shared by both lacertids; fixed differences) in each block. 1965 of these blocks appeared more than once in the data (Supplementary Information 3).

We compared the AIC scores of thirteen different demographic scenarios (Table 1) given the pattern of bSFS between the two lacertid species (Supplementary file 3). However, this composite likelihood computation does not account for the correlation between adjacent blocks due to the physical linkage. To correct for this, we assumed that every 1000th block is effectively unlinked (Supplementary Information 3 Section LD), i.e. statistically independent, and corrected lnC:L scores by a factor of 1/200.

The best of the thirteen models (M3.3) supports isolation between the two lacertid species with unidirectional gene flow from *L. bilineata* to *L. viridis* and fits significantly better than simpler scenarios like divergence without gene flow (or just a single N_e parameter) or admixture (Table 1). This model (M3.3) also suggests a smaller effective population size of *L. bilineata* (Ne=37890) compared to its ancestor and *L. viridis* (Ne=95400) (supplement SI-4; Table S6) and a migration rate of M=0.28 migrants per generation from *L. bilineata* to *L. viridis* (Table S7).

Assuming a generation time of 3.5 years and a mutation rate of 1.77×10^{-8} (based on cyt-b gene) or 1×10^{-9} (based on the fourfold degenerate sites), our estimate of the split between *L. viridis* and *L. bilineata* corresponds to 1.15 Mya and 20.37 Mya respectively (Table S8).

Detection of genomic rearrangements

We detected 20,160 genomic rearrangements or structural variants (SVs) longer than 50bp between the two lacertids (Figure 2; Table S9) covering 39.4 Mbp of the *L. viridis* genome (2.7% of the genome). Compared to *L. bilineata*, 10.8 Mbp (0.07%) of the *L. viridis* genome was covered with large rearrangements affecting genes (covering the entire length of more than one gene). These regions were enriched for RNA-directed DNA polymerase activity (22.46 fold-enrichment, p=5.11e-03).

Indels are the most frequent genomic rearrangements mainly affecting introns, repeat elements and pseudo-tRNAs (supplement SI-5; Table S10). This is similar to the observations made with respect to SVs in humans and pigs [12, 53]. Most SVs overlapping exons cover entire exons and do not result in frame shift mutations, with the exception of EXD2 and HERC2, suggesting that their functions can be complemented by other genes (supplement SI-6).

Structural selection of ncRNAs

MicroRNAs (miRNA) were the most structurally conserved family of ncRNAs followed by small non-coding RNAs (snoRNA) (Figure S9). The four types of ncRNAs and the number of groups in each category are shown in Table S11 (supplement SI-7). High levels of diversity were observed in tRNAs, especially in pseudo-tRNA, which was further supported by high copy numbers of tRNAs with a low conservation among tRNA orthologs between the two lacertid species.

All ncRNAs with low structural diversity across orthologs were computationally tested for sites with positive selection in either species. The positively selected snoRNA families belong to the H/ACA box class, which can introduce changes in post-translational mechanisms and pseudouridylation between the two species [54]. SNORD61 (Small Nucleolar RNA, C/D Box 61) (Figure S10a) was inferred to have evolved under positive selection in *L. bilineata*. The human ortholog of SNORD61 occurs in the intron of a RBMX (RNA binding motif protein, X-linked gene), known to be involved in the dosage compensation and cohesion regulation of sister chromatids [55]. Two microRNAs showed signs of positive selection in *L. viridis*: MIR6516 (mir-6516-3p) (Figure S10b), associated with urea synthesis in pigs [56] and MIR27 (mir-27a and mir27-d) (Figure S10c), known to play a role in regeneration and osteoblast differentiation in mice [57, 58]. However, mir-27d was absent in *L. bilineata*, so the structural divergence in the mir-27 family between the two lacertid species can be due to the presence of an additional MIR27 sequence in *L. viridis.* Two lincRNAs orthologs (LiNC66 and LiNC29) overlapping with conserved regions across tetrapods were structurally divergent between the two species, as indicated by high selection scores and stable secondary structures (Figure S10d-e).

Varying selection pressures in protein-coding genes

The visual opsins are pivotal for adaptation to diurnal habitats in Squamata [24, 59]. For instance, the nocturnal *G. japonicus* lost two of the five functional opsin paralogs compared to diurnal anoles [26]. All five paralogs of visual opsins in *A. carolinensis* (22 transcripts from ENSEMBL) were also present in *L. viridis* and *L. bilineata* (20 transcript sequences), indicating conservation of genes for diurnal vision. We observed high conservation of SWS1 (opsins related to UV vision), described to be involved in sexual selection [60, 61] and of the pigmentation protein MC1R, previously associated with adaptive colouration in sand lizards [62] (supplement SI-8).

Genes involved in neuronal activity, behaviour, auditory perception and female reproductive system development were conserved in the lacertid ancestor i.e. before the split between the two species (compared to five other vertebrates in the background). Genes with different selective constraints between the two species (i.e. differently influenced by purifying selection after their split) were related to brain and neural development, embryo and cartilage development along with behavioural responses (Table S12).

The test for positive selection in either of the two species was performed with the branch-site model of codeml (model M2) using a subset of other lizards as background branches. The number of genes with positively selected sites (PSS) in different foreground branches (*L. viridis*; *L. bilineata*; or the ancestor of *L. viridis* and *L. bilineata*) are shown in the Table S13 (supplement SI-9). The predicted ontologies of genes with PSS in either of the two species indicate variation in growth and developmental processes, behavioural responses (temperature and pH) and transcriptional regulation (Table S14). One of the genes with PSS in *L. bilineata* (STAR7) is located on the Z-chromosome. We identified two transcription factor genes, UBIP1 and RPA2 involved in gene silencing and reproductive functions [63, 64], with adaptive differences between the two species. Three genes with PSS overlapped inverted regions; GPR155 gene with PSS in *L. bilineata*, both TDRD3 and UGPA with PSS in *L. viridis*. GPR155 is involved in cognitive functions and expressed in mice forebrain [65] while TDRD3 is directly associated with oocyte formation and X-linked developmental disorders [66, 67]. Three genes NASP, PDL11 and RTKN were positively selected in the ancestor of the lacertid branch compared to background branches that include more distant classes such as mammals and birds (supplement SI-9, Table S15).

The prostacyclin synthase (PTGIS) involved in regeneration through prostaglandin synthesis is positively selected in *A. carolinensis* and *G. japonicus* [26]. This gene evolved under positive

selection in the lacertid ancestor with *A. carolinensis* and *G. japonicus* as the background, hinting at evolutionary changes in regenerative mechanisms among lacertid lizards.

Diversification of UV-responsive genes

We identified three paralogs of the hyaluronidases (HYAL1, HYAL2 and HYAL4) in both the lacertid genomes. Two genes (STIK1 and HYAL2) coding for proteins in the extra-cellular matrix of the skin reacting to UV-B light (GO:0071493) [68] were positively selected in the ancestral branch of the two species, while the HYAL1 paralog was positively selected in the *L. viridis* branch (supplement SI-8). Arylsulfatase gene (ARSB) which is involved in the chondroitin sulfate biosynthesis pathway along with HYAL was also positively selected in *L. viridis*. Significant pathway enrichment of chondroitin sulfate biosynthesis was observed for PSGs in *L. viridis* (p=2.6e-06, q=1.3e-05).

Divergence of Kruppel-type zinc-finger (KZNF) proteins

To investigate the role of KRAB-ZNFs in reproductive isolation of the two lacertid species, we compared the DNA-binding domains of KZNF orthologs. From the 53 KZNF orthologs, six C2H2 zinc-finger proteins showed binding specific differences between the two lacertid species (supplement SI-10). While the longest transcripts of these six KZNFs were assembled from ovarian tissues (Table S16), they were also expressed in all the other tissues analyzed (brain, heart, liver and kidneys).

Impact of rearrangements on sequence evolution

Deletions are the most frequent type of SVs in the genome and occurred on both positively selected genes and those with no signs of positive selection. Duplications and insertions only occurred in genes evolving without signs of positive selection while deletions and inversions occurred in genes irrespective of their selective regime. The ratio between number of regions

with rearrangements or SVs to those with no detected rearrangements was not significantly different between genes under positive selection and those with no signs of positive selection (Boschloo's exact test, two-sided; difference in proportion=0.125, p=0.06, q=0.1). Since this can be due to abundant indels obscuring the association in other categories of SVs, we tested the association between each SV category with PSGs separately, applying independent Boschloo exact-tests (Table S17). An association of PSGs within inversions when compared to other SV categories was observed, but this did not remain significant after multiple testing (p=0.028, q=0.06). We also observed a significant association of PSGs over genes with no signs of positive selection within inversions compared to both non-rearranged regions (p=0.009, q=0.03) and collinear regions (p=0.006, q=0.03). The inversions overlapping PSGs seem to reflect independent events, since the inversions are located on different contigs in the genome with size ranges between 70 kbp and 700 kbp.

Discussion

We provide the assembled genomes of two closely-related lacertid species, *L. viridis* and *L. bilineata*, investigated their history of divergence and analyzed the patterns of genomic variation between these species.

The assembly contiguity was highest with partial error correction of PacBio reads (without splitting at chimeric junctions) followed by hybrid assembly through DBG2OLC implementing removal of chimeric joins. This hybrid assembly strategy aided in generating high quality contig-level genomes with moderate genome coverages (~35X Illumina and ~15X PacBio). Our lacertid genome assemblies showed higher completeness than the available lizard genomes (Table S1).

The time of population divergence between *L. viridis* and *L. bilineata* was estimated as at least 1.15 Mya which is lower than the mitochondrial divergence time of 2.6-3.4 Mya previously estimated [33, 69]. *L. viridis* and *L. bilineata* show a high level of genome-wide differentiation (F_{ST}=0.688). The best demographic model (M3.3) supported unidirectional gene flow from *L. bilineata* to *L. viridis* and higher effective population size for *L. viridis* than *L. bilineata* consistent with the difference in genetic diversity between the two lacertid species.

Species-specific diversity within various ncRNA classes and adaptive differences in ncRNA orthologs capable of altering their secondary structures are two important factors contributing to evolutionary divergence, since varying ncRNA structures imply functional changes [17]. Copy number variation and differences in the content of miRNA families hint at variability in gene regulatory networks between the lacertid sister species. Species-specific splicing mechanisms can be attributed to the losses of snoRNA families (SNORA17 and SNORA20) in *L. bilineata* and structural changes in SNORD61, which is involved in dosage compensation in humans [70].

Positive selection of sites in NASP and PDLIM1 in the lacertid ancestral branch compared to distant background branches, including mammals and birds, may indicate disparate evolutionary changes in the ancestor of *L. viridis* and *L. bilineata* with regard to reproductive processes i.e. spermatogenesis, fertilization and embryo implantation [71-74]. In contrast, positive selection acting on coding sites in just one lacertid species after their split suggests adaptive differences which could play a role in the speciation process [75-77].

UV-reflectance of plumages in birds is an important trait involved in the sexual selection of morphologically similar sibling species of Passeriformes [78]. Sexual selection in *L. viridis* has been linked to UV-response. Males with more UV-reflective patches on the skin are preferably selected by the females [79, 80]. We show that hyaluronidases, known to be differentially

expressed on exposure to UV-B in the skin of mice [68, 81, 82] evolved rapidly in *L. viridis*. We speculate that differential cutaneous response to UV through changes in the chondroitin sulfate (CS) biosynthesis pathway could be driven by mating preferences, which could ultimately contribute to speciation. Further studies are needed to test this hypothesis.

KRAB-ZNFs or KZNFs (zinc finger proteins with a *Krüppel*-associated box (KRAB)) are transcriptional regulators confined to tetrapod vertebrates [83] and are known to play a role in species specific changes in gene regulatory network through binding domain differences between humans and chimps [6, 84-87]. The divergence of transcription factors, especially differences in DNA-binding regions of KZNFs as observed here, could eventually have contributed to some degree of reproductive isolation between the two species, which should be further tested. This receives further support from adaptive differences in the transcription factors (UBIP1 and RPA2) crucial for spermatogenesis [63, 64]. Varying levels of purifying selection in genes influencing forebrain development and behaviour suggest different selective constraints between *L. viridis* and *L. bilineata*. The behavioural differences can be related to varying ecological habitats and environmental conditions [35] after the split of *L. viridis* and *L. bilineata*. Selective differences in genes related to behaviour and brain development have been reported to be involved in the diversification of anoles [32].

Genomic regions harbouring inversions are known to suppress recombination in heterokaryotypes facilitating speciation in the presence of gene flow [88] and in maintaining favourable combinations of locally adapted alleles at different loci [89]. Genomic inversions between the two lacertid species are significantly associated with positively selected genes (PSGs). Two of the three PSGs occurring within inversions play a role in cognitive and reproductive functions (GPR155 and TDRD3), suggesting that they could be involved in speciation. However, it is currently unknown if these inversions represent fixed differences between the two species and the lengths of these inversions is at a lower scale (less than 1 Mbp)

than those known to play a role in adaptation and speciation [10]. Future studies should try to address these issues, as well as the role of these genes in reproductive isolation between *L. viridis* and *L. bilineata*.

Conclusions

We assembled the first high quality genomes of two closely-related species of European green lizards with a cost-effective strategy. Genes related to transcriptional regulation, behaviour, neural and reproductive development have diversified the most between the two lacertid species. Species-specific diversity of ncRNAs, adaptive evolution in regulatory elements and transcription factors (including binding domain differences in KZNFs) indicate variation in gene regulatory networks between the two species. Adaptive evolution of genes responsible for differential cutaneous response to UV-exposure, in particular, could be driven by mate choice, and ultimately contribute to reproductive isolation. Altogether, we provide the first comprehensive study of the evolutionary history, genic, structural and regulatory differences between the genomes of two closely-related lacertid species. This comprises an important baseline for understanding the genomic regions and mechanisms involved in the speciation of European green lizards. In addition to a detailed analysis of the demographic history and evolutionary scenario of European green lizards, our study provides valuable resources that will help establish conservation guidelines for lacertids experiencing population declines due to habitat loss [90].

Materials and Methods

Sampling

Two adult females were sampled for this study, a *L. viridis* from Tokaj, north-eastern Hungary (21.39775ºE, 48.11363ºN) (September 2013) and a *L. bilineata* from Mâlain, France (4º48'2.01"E, 47º21'16.27"N) (July 2014). There is no known morphological variation between the individuals of the two species (Figure S1). These represent two of the four main clades within the *L. viridis* complex [33, 34, 38, 91]. Animals were captures with permits of the issuing authorities (please refer to acknowledgements) and handled according to the guidelines of the Herpetological Animal Care and Use Committee (HACC) of the American Society of Ichthyologists and Herpetologists. Tissues from the brain, heart, liver, kidney and ovaries were dissected for tissue-specific transcriptome sequencing and the remaining tissues were stored separately at -80°C.

Whole-genome and transcriptome sequencing

Tail tissue from each sample was digested with proteinase K and genomic DNA was extracted using a chloroform-based method [92]. The whole genome was sequenced using both short (Illumina) and long read (PacBio) sequencing techniques. Short-read libraries with insert sizes of 380bp and 450bp were prepared for each individual separately. The Illumina paired-end sequences were double-indexed using a multiplexing sequencing protocol [93, 94] on a HiSeq2500. SMRTbell™ template library was prepared according to the instructions from PacificBiosciences, Menlo Park, CA, USA, following the Procedure and Checklist – Greater Than 10 kb Template Preparation. Briefly, for preparation of 15kb libraries 10µg (*L. bilineata*) and 20µg (*L. viridis*) genomic DNA was damage-repaired twice, end-repaired and ligated overnight to hairpin adapters applying components from the DNA/Polymerase Binding Kit P6 from Pacific BioSciences, Menlo Park, CA, USA. Reactions were carried out according to the manufacturer's instructions. BluePippin™ Size-Selection to greater than 15kb was performed according to the manufacturer's instructions (Sage Science, Beverly, MA, USA). Conditions

for annealing of sequencing primers and binding of polymerase to purified SMRTbell™ template were assessed with the Calculator in RS Remote, PacificBiosciences, Menlo Park, CA, USA. Long-read sequencing was carried out for both genomes with 20 SMRT Cells applying P6-C4 chemistry on a PacBio RS-*II* sequencer. Average PacBio read lengths of 14kb and 12kb were retrieved for *L. viridis* and *L. bilineata*, respectively.

RNA from each tissue was extracted using Trizol Reagent (Life Technologies, Carlsbad, CA, USA) and purified with the RNeasy® Mini Kit (Qiagen, Hilden, Germany). The mRNA was purified using the Dynabeads® mRNA Purification Kit (Life Technologies, Carlsbad, CA, USA). The purity and concentration of RNA and cDNA were checked using Nanodrop and Bioanalyzer 2100 (Agilent Technologies, CA, USA) and fragments of length 200-250bp were obtained using Ambion® RNA fragmentation reagent. The first and second strands of cDNA were synthesized using random hexamer primers with SuperScript® II reverse transcriptase (Life Technologies, Carlsbad, CA, USA) and DNA Polymerase I with RNase H treatment (Life Technologies, Carlsbad, CA, USA) respectively.

L. viridis was sequenced on a single lane for a more accurate estimate of genome size and repeat content. In order to avoid lane- and run-biases, sequencing was distributed over three lanes with all genomes and transcriptomes.

Non-coding RNA (ncRNA) annotation and Repeat analysis

Small ncRNAs were annotated on the genomic contigs by performing an infernal cmscan (V1.1.1) using the RFAM covariance models as input and homologous ncRNA genes were filtered with a cut-off of 1e-06 [95, 96].

Additionally, ncRNA class-specific annotation methods were used for tRNAs, snoRNAs and miRNAs. tRNAs were annotated using tRNAscan-SE with default parameters [97]. The BLAST-based snoStrip pipeline [98] was used to annotate snoRNAs. A comprehensive set of

snoRNAs from vertebrates and aves were used as query set [99]. To detect miRNAs, the avian set of miRNAs were used as query sequences for a BLAST search in the lizard genomes. All resulting blast hits were filtered for the conservation of the seed region. The annotated snoRNAs and miRNAs in lacertids were validated by blast searches against this reference database and mature miRNA sequence homologies were used. In the case of overlapping miRNA and snoRNA annotations, both were retained as it is known that snoRNAs can be processed into small derived RNAs (sdRNAs) from miRNA-like RNAs [100]. Putative lincRNAs were predicted based on the transcripts with no coding potential as assessed by Transdecoder [101] and mapping on their respective genome without chimeric paths. Furthermore, only the conserved lincRNAs with one-to-one orthologs between lacertids were retained.

For comparison, ncRNA families (except lincRNA) were also annotated in other selected sauropsid genomes. A reference database was created using sequenced and annotated genomes from reptiles, aves and other vertebrates. The program ePoPe [102] was used to understand the evolution of snoRNAs and miRNAs in the lacertids through the construction of phylogenetic trees based on the gains and losses of ncRNA families.

The Repeatmodeler pipeline [103] was used to predict repeats in the genomes of lacertids. The predicted repeat-families were used as initial libraries for *de novo* annotation of repeats using Repeatmasker [104]. The evolution of these repeats was investigated using the repeat library available for tetrapod species (Database: 20140131).

Population histories, gene flow and coalescence

To assess the demographic history between *L. viridis* and *L. bilineata* we used the blockwise composite likelihood approach. We analytically computed the probabilities of mutational configurations in blocks of fixed length using the blockwise site frequency spectrum (bSFS) framework [20].

We mapped the illumina reads from *L. viridis* and *L. bilineata* to the *L. viridis* reference genome with BWA MEM [105]. The homozygosity/heterozygosity of each site in both lacertids was predicted based on the reference genome with freebayes [106] with a minimum read support of five and minimum allele frequency of 0.2. The intergenic regions of the genome were chopped into blocks of length 200 bp, this resulted in 5,654,020 blocks in total. The number of four mutation types defined by the joint SFS (Figure 3) were counted using Heffalump query (https://bitbucket.org/ustenzel/heffalump commit 7773784). In total, 2785 distinct mutational configurations were obtained, of which 1965 appeared more than once. We then summarized the frequency of each polymorphism pattern across all blocks [107, 108]. This data summary is referred to as distribution of bSFS.

Blocks containing both fixed differences and shared heterozygous sites, violate the 4-gametes criterion and 46,825 blocks (0.83%) were removed under the assumption of no recombination within blocks. To account for physical linkage between adjacent blocks, we assumed that every 1000th block is effectively unlinked, i.e. statistically independent, and corrected lnC:L scores by a factor of 1/200. We fitted thirteen different demographic scenarios (Figure 2) accounting for the presence or absence of gene flow, direction of gene flow, continuous or discrete migration and changes in effective population sizes. Models were compared using the Akaike information criterion (AIC) of their composite log-likelihoods.

We estimated the generation length based on the mean age of the mothers of all offspring [109] given the age structure data by Elbing [110] and Saint Girons *et al.* [111] for three German populations of *L. viridis* and two French populations of *L. bilineata*, respectively. In captivity, females that breed for the first time lay on average 8.5 eggs, whereas older females lay 11.1 eggs [112]. Given this data, we estimated a mean generation length of 3.6 and 2.9 years for *L. viridis* and *L. bilineata*, respectively. We therefore assumed a generation time of about 3-4 years for both species. To scale our result in real-time, we used a mutation rate of 1 x 10^{-9} per site per

generation based on the fourfold degenerate sites of the single-copy gene orthologs between *L. viridis* and *L. bilineata* (supplementary methods SM-4). The lower limit of the mutation rate was assumed as 1.77 x 10^{-8} per site per generation (3.5 years as the generation time) based on the pairwise distance of 6.19% in the cytochrome b gene between *L. viridis* and *L. bilineata* [34]. This assumption is similar to the mutation rate of NADH-2 in *A. carolinensis* (1.3% mutations per million years) [113].

Detection of genomic rearrangements from read-based pipelines and syntenic blocks

Genomic rearrangements between the lacertids were detected based on both read-based methods and syntenic blocks information. *L. viridis* was used as the reference genome since the assembly was more contiguous for this species. Genomic reads from *L. bilineata* were used as the query and the reads of *L. viridis* mapped against the reference were used as control.

Read-based pipelines: Genomic rearrangements were detected between lacertids using read mapping based methods for Illumina paired-end reads and for PacBio-reads separately, followed by SV callers specifically developed to deal with short and long read sequences, respectively. In both approaches, reads of *L. bilineata* (query) and of *L. viridis* (control) were separately mapped against the same reference (*L. viridis*).

The alignment of Illumina reads was carried out with BWA MEM [105] and rearrangements were detected with MetaSV [114] pipeline which uses Breakdancer [115] to infer structural variants (SVs) using paired-end read information, CNVnator [116] to predict copy-number variants (CNVs) from abnormal read-coverages and Pindel [117] to detect large SV-related breakpoint events. The insert-size was estimated as 400±50 from one million observations based on the alignment of paired-end Illumina reads. A minimum support of five reads and mapping quality of 30 was set as the threshold to support SVs from Breakdancer. A bin-size of 500 was used to run CNVnator and only precise SV-events were called. While for Pindel, only

variants with minimum read support of 5 paired-reads were used. MetaSV pipeline was used to merge the SVs from these three different SV-callers and local *de novo* assemblies were constructed using the ABYSS assembler for insertions. In order to maintain a high level of sensitivity and specificity (>90%) in the detection of SVs, only the rearrangements called with a minimum support of 8 uniquely mapped paired-end reads were used for further analyses [118].

The PacBio reads were aligned to the reference with NGMLR and the alignment was fed to Sniffles SV-caller [11] to call variants with a minimum support of seven reads (at least half of the PacBio genome coverage of 14X).

Syntenic blocks approach: In addition to read-based methods, rearrangements were also detected from the blocks of synteny obtained through the UCSC pipeline [119]. The alignments were converted to single-coverage genomes using single_cov2 of the MultiZ pipeline [120] to avoid spurious assignments. Strand changes within syntenic blocks were clustered as inversions (I) based on the orientation of the successive (I+1) and preceding (I-1) blocks. Regions with missing bases in the query alone were predicted to be deletions while gaps in the reference genome alone were considered as insertions. Additionally, Hierarchical Alignment (HAL) format [121] of the single-coverage genomes was used to predict rearrangements with the halBranchMutations tool. This tool generates annotations for the location of rearrangements based on the branch of interest in the HAL file (between *L. viridis* and *L. bilineata* in our case). The events detected in both directions i.e. *L. viridis* reference with *L. bilineata* as query and *L. bilineata* reference and *L. viridis* as query were retained. The length threshold was set to 50bp and the predicted rearrangements were filtered based on quality to reduce false-positives (supplement SM-7).

Segmental duplications in the two lacertid species were detected by self-aligning the two genomes separately with chained LASTZ [122] (step=9, H=3000, K=5000). High identity

matches (90% identity) within each genome of 1kb or more were defined as segmental duplications.

Structural selection in non-coding RNAs (ncRNA)

The predicted ncRNAs (miRNA, snoRNA, tRNA and lincRNA) in lacertids were tested for structural selection (selection of sites acting on secondary structure in either of the lacertids) with *Gekko japonicus* as outgroup. We used the Selection on the Secondary Structure test (SSS-test) [123], a statistical test that assigns selection scores for each given sequence based on the comparison between the structure of the given sequence and the structure of group consensus. It also provides a diversity value for the family that indicates its structural conservation. The diversity value (d-score) is the family's median vase-pair distance to its consensus. The miRNAs, snoRNAs and tRNAs were divided into sub-groups based on their families or their anti-codon sequences, and only those sub-groups with at least three sequences were tested. The groups that exhibited high structural diversity (median base pair distance to the consensus, $d \geq 10.0$) were excluded from further analyses.

A ncRNA structural test to detect positively selected structures is only appropriate for structurally conserved groups. Low d-score values ($d<10.0$) were used to distinguish conservation chosen based on structural uniformity of the groups. This cut-off was based on the visual inspection of the secondary structures of families with d-scores of 1 to 20. Secondary structures of ncRNA sequences were predicted using RNAfold [124]. In a similar fashion, structures with selection scores of 0 to 30 were visually compared to the structure of their group consensus. High selection scores ($s \geq 10.0$) were used to predict the positively selected sequences of small ncRNAs. Secondary structures with high selection scores were manually inspected to remove false positives. Specifically, the candidates with structures of low stability or those fundamentally dissimilar to the family consensus indicating loss of function were excluded.

The selection test was adapted for lincRNAs and performed only on the two lacertid species without any outgroup since lincRNA annotations of other closely-related species were unavailable. Since the positive selection of secondary structure cannot be determined without outgroups, we instead detected divergence of lincRNA structure within the lacertids. Local conserved structure blocks were predicted for the orthologous lincRNA families and these blocks were subjected to an adaptation of SSS-test based on local structures. The structural selection for lincRNAs was assessed locally, since most base-pairings occur between nucleotides within a short distance [123, 125]. Local blocks of high structural diversity were excluded from further analysis. Since outgroups were not used for lincRNAs, a lower selection score threshold ($s \geq 4.0$) was applied to detect divergent candidates which were visually inspected later to exclude false-positives.

Ortholog prediction and selection tests

In order to investigate the selection pressure in the lacertid branch (ancestor of *L. viridis* and *L. bilineata*) compared to other vertebrates, the coding sequences (CDS) of five species, namely anole lizard (*Anolis carolinensis*), chicken (*Gallus gallus*), frog (*Xenopus tropicalis*), spotted garfish (*Lepisosteus oculatus*) and human (*Homo sapiens*) were downloaded from the Ensembl database version 83 [126]. To keep the data consistent and avoid re-annotations, the CDS annotations were also extracted from the Ensembl database. The orthologs between the protein-coding sequences of the species were identified with ProteinOrtho V5 using the synteny option to reduce false orthologs assignments. The output was converted to run the POTION pipeline [127] which tests for selection acting on protein coding genes. Only the single-copy orthologs in each species were retained for each orthologous group.

The protein identity filtering in POTION was set to 30% in each orthologous group and sequence size limits to more than 10 times or less than 0.2 of the median size in the group. Only groups with at least 4 species were retained. The sequences in each orthologous group (after

filtering paralogs) were aligned, gap trimmed, phylogenetic trees were constructed and groups with recombinants were excluded from the selection tests. The intermediates files from the POTION pipeline were used to generate unrooted trees with lacertids (*L. viridis* and *L. bilineata*) in the foreground branches. The remaining species were used as the background to test for positive-selection using the branch-site model of codeml within the PAML package[128]. A likelihood ratio test (LRT) based on χ^2 distribution was used to detect genes with significant positive selection followed by multiple testing through Benjamini–Hochberg (BH) procedure. Genes with $p<0.05$ and $q<0.05$ were retained and referred to as being positively selected in the lacertid branch.

To detect adaptive evolution through positive selection within either lacertids, additional tests (PAML branch-site models) were performed with less distant outgroups using a set of five lizard species, namely *L. viridis*, *L. bilineata*, *Anolis carolinensis*, *Gekko japonicus* and *Pogona vitticeps*. The single-copy orthologs were identified with ProteinOrtho with a minimum protein identity of 70%, e-value of 1e-06 and minimum similarity of 0.99 for additional hits. The orthologous coding sequences from the five lizard species were aligned with MACSE while accounting for frame-shifts and the stop codon at the end of the sequence was removed. Unrooted trees were generated with three different foreground branches: i) lacertids (*L. viridis* and *L. bilineata*) ii) *L. viridis* alone and iii) *L. bilineata* alone. The rest of the workflow for detection of recombinants, removal of gaps and codeml tests was similar to the POTION pipeline followed by filtering for significant candidate genes ($p<0.05$, $q<0.05$). In order to avoid false predictions of positively selected sites (PSS) at the beginning or towards the end of alignments, where mismatches were allowed, the candidate genes predicted to contain PSS in either species were visually inspected.

Data Access

The genome assembly, transcript data, DNA and RNA sequencing reads have been deposited in the European Nucleotide Archive under the Bioproject PRJEB24178.

GCA_900245905 - *L. viridis* genome assembly

GCA_900245895 - *L. bilineata* genome assembly

The transcript assemblies, genome browser and online BLAST databases for the lacertid data are hosted at http://lacerta.bioinf.uni-leipzig.de

Genome annotations, variant calls (VCFs) and other supporting datasets are available at http://doi.org/10.5281/zenodo.1219810

The supporting files for the demography analysis including the Mathematica notebooks input files are provided at https://bitbucket.org/amin-saffari/reversegame/src/master/

Declarations

Acknowledgements

The authors thank Annegrett Grimm for the sample collection and providing the photos of the two species; Conrad Helm, Detlef Bernhard, Michael Gerth, Ronny Wolf and Stefan Schaffer for their help in the laboratory; Steve Hoffmann and Thomas Hackl for valuable discussions and Stephane Peyrégne for helpful suggestions and proofreading. The specimen from Hungary was kindly collected and provided by the team of Szabolcs Lengyel, Centre for Ecological Research, Hungarian Academy of Science. Capture permit (No. 13778-7/ 2013) was issued by the North Hungarian Environmental Protection, Nature Conservation and Water Management Inspectorate. Capture permit (No. 36) for the French specimen was issued by the Préfet de la

Côte d’Or. RF was financed by FCT [grant number SFRH/BPD/89313/2012] and is currently funded by the European Union’s Horizon 2020 research and innovation programme, under the Marie Sklodowska-Curie grant agreement number 706376.

Funding

This project was funded by the Deutsche Forschungsgemeinschaft (FZT 118).

Declaration of interest

The authors report no conflicts of interest. The authors alone are responsible for the content and writing of the paper.

Author contributions

K. H. collected the samples; A. W. Illumina sequenced the genome and transcriptome; C. S., B. B. and J. O. were involved in the PacBio genome sequencing; S. R. R. K. performed the assembly and annotation; S. K. and S. R. R. K. annotated the non-coding RNA; M. B. W. C. analyzed selection in non-coding RNA; S. R. R. K. and H. I. performed the positive selection analysis; S. R. R. K. and M. C. analyzed structural variants; A. S. and K. L. analysed the population histories; S. R. R. K., R. F., K. N., P. F. S. and M. S. wrote the initial draft of the manuscript; H. I., M. C., A.S., K. L., S. K., M. B. W. C., J. O., B. B., C. B. and K. H. edited the manuscript; R.F., K. N., K. H., P. F. S. and M. S. conceived the study.

Additional files

Additional file 1: This supplement contains methods SM1-SM11, information SI1-SI11, Figures S1–S10, Tables S1–S17 and References.

Additional file 2: The figure for the contig clusters in lacertids generated from synteny information between *L. viridis* and *L. bilineata.*

Additional file 3: *Mathematica* notebook containing the code used and other supporting information from the demography analysis of *L. viridis* and *L. bilineata.*

References

1. Coyne JA, Orr HA: **Speciation**: Sinauer Associates; 2004.
2. Presgraves DC: **The molecular evolutionary basis of species formation**. *Nat Rev Genet* 2010, **11**(3):175-180.
3. Charlesworth B, Coyne JA, H. Barton N: **The Relative Rates of Evolution of Sex Chromosomes and Autosomes**. *American naturalist* 1987, **130**(1):113-146.
4. Masly JP, Presgraves DC: **High-resolution genome-wide dissection of the two rules of speciation in Drosophila**. *PLoS Biol* 2007, **5**(9):e243.
5. Mack KL, Campbell P, Nachman MW: **Gene regulation and speciation in house mice**. *Genome Res* 2016, **26**(4):451-461.
6. Nowick K, Carneiro M, Faria R: **A prominent role of KRAB-ZNF transcription factors in mammalian speciation?** *Trends Genet* 2013, **29**(3):130-139.
7. Ortiz-Barrientos D, Kane NC: **Meeting review: American Genetics Association Symposium on the genetics of speciation**. *Mol Ecol* 2007, **16**(14):2852-2854.
8. Ravinet M, Faria R, Butlin RK, Galindo J, Bierne N, Rafajlović M, Noor MAF, Mehlig B, Westram AM: **Interpreting the genomic landscape of speciation: a road map for finding barriers to gene flow**. *Journal of Evolutionary Biology* 2017, **30**(8):1450-1477.
9. Seehausen O, Butlin RK, Keller I, Wagner CE, Boughman JW, Hohenlohe PA, Peichel CL, Saetre GP, Bank C, Brannstrom A *et al*: **Genomics and the origin of species**. *Nat Rev Genet* 2014, **15**(3):176-192.
10. Wellenreuther M, Bernatchez L: **Eco-Evolutionary Genomics of Chromosomal Inversions**. *Trends in Ecology & Evolution* 2018, **33**(6):427-440.
11. Sedlazeck FJ, Rescheneder P, Smolka M, Fang H, Nattestad M, von Haeseler A, Schatz MC: **Accurate detection of complex structural variations using single-molecule sequencing**. *Nature Methods* 2018.
12. Sudmant PH, Rausch T, Gardner EJ, Handsaker RE, Abyzov A, Huddleston J, Zhang Y, Ye K, Jun G, Hsi-Yang Fritz M *et al*: **An integrated map of structural variation in 2,504 human genomes**. *Nature* 2015, **526**(7571):75-81.
13. Alkan C, Coe BP, Eichler EE: **Genome structural variation discovery and genotyping**. *Nat Rev Genet* 2011, **12**(5):363-376.
14. Davey JW, Barker SL, Rastas PM, Pinharanda A, Martin SH, Durbin R, McMillan WO, Merrill RM, Jiggins CD: **No evidence for maintenance of a sympatric Heliconius species barrier by chromosomal inversions**. *Evolution Letters* 2017, **1**(3):138-154.
15. Walter Costa MB, Honer Zu Siederdissen C, Tulpan D, Stadler PF, Nowick K: **Temporal ordering of substitutions in RNA evolution: Uncovering the structural evolution of the Human Accelerated Region 1**. *J Theor Biol* 2017, **438**:143-150.
16. Frías-Lasserre D, Villagra CA: **The Importance of ncRNAs as Epigenetic Mechanisms in Phenotypic Variation and Organic Evolution**. *Frontiers in Microbiology* 2017, **8**:2483.
17. Bhartiya D, Scaria V: **Genomic variations in non-coding RNAs: Structure, function and regulation**. *Genomics* 2016, **107**(2-3):59-68.
18. Gardner PP, Fasold M, Burge SW, Ninova M, Hertel J, Kehr S, Steeves TE, Griffiths-Jones S, Stadler PF: **Conservation and losses of non-coding RNAs in avian genomes**. *PLoS One* 2015, **10**(3):e0121797.
19. Sousa V, Hey J: **Understanding the Origin of Species with Genome-Scale Data: the Role of Gene Flow**. *Nature reviews Genetics* 2013, **14**(6):404-414.
20. Lohse K, Chmelik M, Martin SH, Barton NH: **Efficient Strategies for Calculating Blockwise Likelihoods Under the Coalescent**. *Genetics* 2016, **202**(2):775-786.

21. Camargo A, Sinervo B, Sites JW, Jr.: **Lizards as model organisms for linking phylogeographic and speciation studies**. *Mol Ecol* 2010, **19**(16):3250-3270.

22. Thorpe RS, Surget-Groba Y, Johansson H: **The relative importance of ecology and geographic isolation for speciation in anoles**. *Philosophical Transactions of the Royal Society B: Biological Sciences* 2008, **363**(1506):3071.

23. Alfoldi J, Di Palma F, Grabherr M, Williams C, Kong L, Mauceli E, Russell P, Lowe CB, Glor RE, Jaffe JD *et al*: **The genome of the green anole lizard and a comparative analysis with birds and mammals**. *Nature* 2011, **477**(7366):587-591.

24. Gao J, Li Q, Wang Z, Zhou Y, Martelli P, Li F, Xiong Z, Wang J, Yang H, Zhang G: **Sequencing, de novo assembling, and annotating the genome of the endangered Chinese crocodile lizard Shinisaurus crocodilurus**. *GigaScience* 2017, **6**(7):1-6.

25. Rice ES, Kohno S, John JS, Pham S, Howard J, Lareau LF, O'Connell BL, Hickey G, Armstrong J, Deran A *et al*: **Improved genome assembly of American alligator genome reveals conserved architecture of estrogen signaling**. *Genome Res* 2017, **27**(5):686-696.

26. Liu Y, Zhou Q, Wang Y, Luo L, Yang J, Yang L, Liu M, Li Y, Qian T, Zheng Y *et al*: **Gekko japonicus genome reveals evolution of adhesive toe pads and tail regeneration**. 2015, **6**:10033.

27. Georges A, Li Q, Lian J, O'Meally D, Deakin J, Wang Z, Zhang P, Fujita M, Patel HR, Holleley CE *et al*: **High-coverage sequencing and annotated assembly of the genome of the Australian dragon lizard Pogona vitticeps**. *GigaScience* 2015, **4**(1):45.

28. Castoe T, Koning A, Hall K, Card D, Schield D, Fujita M: **The Burmese python genome reveals the molecular basis for extreme adaptation in snakes**. *Proc Natl Acad Sci U S A* 2013, **110**.

29. Vonk F, Casewell N, Henkel C, Heimberg A, Jansen H, McCleary R: **The king cobra genome reveals dynamic gene evolution and adaptation in the snake venom system**. *Proc Natl Acad Sci U S A* 2013, **110**.

30. Shaffer H, Minx P, Warren D, Shedlock A, Thomson R, Valenzuela N: **The western painted turtle genome, a model for the evolution of extreme physiological adaptations in a slowly evolving lineage**. *Genome Biol* 2013, **14**.

31. Wang Z, Pascual-Anaya J, Zadissa A, Li W, Niimura Y, Huang Z: **The draft genomes of soft-shell turtle and green sea turtle yield insights into the development and evolution of the turtle-specific body plan**. *Nat Genet* 2013, **45**.

32. Tollis M, Hutchins ED, Stapley J, Rupp SM, Eckalbar WL, Maayan I, Lasku E, Infante CR, Dennis SR, Robertson JA *et al*: **Comparative Genomics Reveals Accelerated Evolution in Conserved Pathways during the Diversification of Anole Lizards**. *Genome Biology and Evolution* 2018, **10**(2):489-506.

33. Böhme MU, Fritz U, Kotenko T, Georg D, Ljubisavljević K, Tzankov N, Berendonk TU: **Phylogeography and cryptic variation within the Lacerta viridis complex (Lacertidae, Reptilia)**. *Zoologica Scripta* 2007, **36**(2):119-131.

34. Marzahn E, Mayer W, Joger U, Ilgaz Ç, Jablonski D, Kindler C, Kumlutaş Y, Nistri A, Schneeweiss N, Vamberger M *et al*: **Phylogeography of the Lacerta viridis complex: mitochondrial and nuclear markers provide taxonomic insights**. *Journal of Zoological Systematics and Evolutionary Research* 2016, **54**(2):85-105.

35. Tvrtkovi N, Lazar B, Tome S, Grbac I: **The western green lizard *Lacerta* (*viridis*) *bilineata* Daudin, 1804 (Sauria: Lacertidae) in Slovenia and Croatia**. *Natura Croatica* 1998, **7**(4):363-369.

36. Amann T, Rykena S, Joger U, Nettmann HK, Veith M: **Zur artlichen Trennung von Lacerta bilineata DAUDIN, 1802 und L. viridis (LAURENTI, 1768)** *Salamandra* 1997, **33**(4):255-268.

37. Joger U, Amann T, Veith M: **Phylogeographie und genetische differenzierung im Lacerta viridis/bilineata komplex**. *Mertensiella* 2001, **13**:60-68.

38. Rykena S: **Kreuzungsexperimente zur Prüfung der Artgrenzen im Genus Lacerta sensu stricto**. *Zoosystematics And Evolution* 1991, **67**(1):55-68.

39. Noor MA, Grams KL, Bertucci LA, Reiland J: **Chromosomal inversions and the reproductive isolation of species**. *Proc Natl Acad Sci U S A* 2001, **98**(21):12084-12088.

40. Navarro A, Barton NH: **Chromosomal speciation and molecular divergence--accelerated evolution in rearranged chromosomes**. *Science* 2003, **300**(5617):321-324.
41. Faria R, Navarro A: **Chromosomal speciation revisited: rearranging theory with pieces of evidence**. *Trends Ecol Evol* 2010, **25**(11):660-669.
42. Olmo E: **Rate of chromosome changes and speciation in reptiles**. *Genetica* 2005, **125**(2-3):185-203.
43. Olmo E, Odierna G, Cobror O: **C-band variability and phylogeny of Lacertidae**. *Genetica* 1986, **71**:63-74.
44. Kupriyanova L, Kuksin A, Odierna G: **Karyotype, chromosome structure, reproductive modalities of three Southern Eurasian populations of the common lacertid lizard, Zootoca vivipara (Jacquin, 1787)**. *Acta Herpetologica; Vol 3, No 2 (2008)* 2008.
45. Liu H, Chang LH, Sun Y, Lu X, Stubbs L: **Deep Vertebrate Roots for Mammalian Zinc Finger Transcription Factor Subfamilies**. *Genome Biol Evol* 2014, **6**(3):510-525.
46. Smeds L, Qvarnström A, Ellegren H: **Direct estimate of the rate of germline mutation in a bird**. *Genome Research* 2016, **26**(9):1211-1218.
47. Nam K, Mugal C, Nabholz B, Schielzeth H, Wolf JBW, Backström N, Künstner A, Balakrishnan CN, Heger A, Ponting CP *et al*: **Molecular evolution of genes in avian genomes**. *Genome Biology* 2010, **11**(6):R68-R68.
48. Chiarelli AB, Capanna E: **Cytotaxonomy and Vertebrate Evolution Edited by A.B. Chiarelli and E. Capanna**; 1973.
49. Townsend TM, Mulcahy DG, Noonan BP, Sites JW, Kuczynski CA, Wiens JJ, Reeder TW: **Phylogeny of iguanian lizards inferred from 29 nuclear loci, and a comparison of concatenated and species-tree approaches for an ancient, rapid radiation**. *Molecular Phylogenetics and Evolution* 2011, **61**(2):363-380.
50. Deakin JE, Edwards MJ, Patel H, O'Meally D, Lian J, Stenhouse R, Ryan S, Livernois AM, Azad B, Holleley CE *et al*: **Anchoring genome sequence to chromosomes of the central bearded dragon (Pogona vitticeps) enables reconstruction of ancestral squamate macrochromosomes and identifies sequence content of the Z chromosome**. *BMC Genomics* 2016, **17**:447.
51. Bermudez-Santana C, Attolini CS-O, Kirsten T, Engelhardt J, Prohaska SJ, Steigele S, Stadler PF: **Genomic organization of eukaryotic tRNAs**. *BMC Genomics* 2010, **11**(1):270.
52. Bunnefeld L, Frantz LA, Lohse K: **Inferring Bottlenecks from Genome-Wide Samples of Short Sequence Blocks**. *Genetics* 2015, **201**(3):1157-1169.
53. Zhao P, Li J, Kang H, Wang H, Fan Z, Yin Z, Wang J, Zhang Q, Wang Z, Liu J-F: **Structural Variant Detection by Large-scale Sequencing Reveals New Evolutionary Evidence on Breed Divergence between Chinese and European Pigs**. 2016, **6**:18501.
54. Kiss T, Fayet-Lebaron E, Jády BE: **Box H/ACA Small Ribonucleoproteins**. *Molecular Cell* 2010, **37**(5):597-606.
55. Matsunaga S, Takata H, Morimoto A, Hayashihara K, Higashi T, Akatsuchi K, Mizusawa E, Yamakawa M, Ashida M, Matsunaga TM *et al*: **RBMX: a regulator for maintenance and centromeric protection of sister chromatid cohesion**. *Cell Rep* 2012, **1**(4):299-308.
56. Sun R-P, Xi Q-Y, Sun J-J, Cheng X, Zhu Y-L, Ye D-Z, Chen T, Wei L-M, Ye R-S, Jiang Q-Y *et al*: **In low protein diets, microRNA-19b regulates urea synthesis by targeting SIRT5**. *Scientific Reports* 2016, **6**:33291.
57. Wang T, Xu Z: **miR-27 promotes osteoblast differentiation by modulating Wnt signaling**. *Biochem Biophys Res Commun* 2010, **402**(2):186-189.
58. Clevers H, Loh KM, Nusse R: **Stem cell signaling. An integral program for tissue renewal and regeneration: Wnt signaling and stem cell control**. *Science* 2014, **346**(6205):1248012.
59. Ricklefs RE, Losos JB, Townsend TM: **Evolutionary diversification of clades of squamate reptiles**. *J Evol Biol* 2007, **20**(5):1751-1762.
60. van Hazel I, Sabouhanian A, Day L, Endler JA, Chang BSW: **Functional characterization of spectral tuning mechanisms in the great bowerbird short-wavelength sensitive visual**

pigment (SWS1), and the origins of UV/violet vision in passerines and parrots. *BMC Evolutionary Biology* 2013, **13**:250-250.

61. de Lanuza GPi, Font E: **Ultraviolet vision in lacertid lizards: evidence from retinal structure, eye transmittance, SWS1 visual pigment genes and behaviour**. *The Journal of Experimental Biology* 2014, **217**(16):2899.
62. Laurent S, Pfeifer SP, Settles ML, Hunter SS, Hardwick KM, Ormond L, Sousa VC, Jensen JD, Rosenblum EB: **The population genomics of rapid adaptation: disentangling signatures of selection and demography in white sands lizards**. *Molecular Ecology* 2016, **25**(1):306-323.
63. Kim JS, Chae JH, Cheon YP, Kim CG: **Reciprocal localization of transcription factors YY1 and CP2c in spermatogonial stem cells and their putative roles during spermatogenesis**. *Acta Histochem* 2016, **118**(7):685-692.
64. Elmayan T, Proux F, Vaucheret H: **Arabidopsis RPA2: a genetic link among transcriptional gene silencing, DNA repair, and DNA replication**. *Curr Biol* 2005, **15**(21):1919-1925.
65. Trifonov S, Houtani T, Shimizu J-i, Hamada S, Kase M, Maruyama M, Sugimoto T: **GPR155: Gene organization, multiple mRNA splice variants and expression in mouse central nervous system**. *Biochemical and Biophysical Research Communications* 2010, **398**(1):19-25.
66. Laisk-Podar T, Kaart T, Peters M, Salumets A: **Genetic variants associated with female reproductive ageing – potential markers for assessing ovarian function and ovarian stimulation outcome**. *Reproductive BioMedicine Online* 2015, **31**(2):199-209.
67. Linder B, Plöttner O, Kroiss M, Hartmann E, Laggerbauer B, Meister G, Keidel E, Fischer U: **Tdrd3 is a novel stress granule-associated protein interacting with the Fragile-X syndrome protein FMRP**. *Human Molecular Genetics* 2008, **17**(20):3236-3246.
68. Tobiishi M, Sayo T, Yoshida H, Kusaka A, Kawabata K, Sugiyama Y, Ishikawa O, Inoue S: **Changes in epidermal hyaluronan metabolism following UVB irradiation**. *Journal of Dermatological Science* 2011, **64**(1):31-38.
69. Kolora SR, Faria R, Weigert A, Schaffer S, Grimm A, Henle K, Sahyoun AH, Stadler PF, Nowick K, Bleidorn C *et al*: **The complete mitochondrial genome of *Lacerta bilineata* and comparison with its closely related congener L. viridis**. *Mitochondrial DNA Part A* 2017, **28**(1):116-118.
70. Sperveslage J, Hoffmeister M, Henopp T, Klöppel G, Sipos B: **Establishment of robust controls for the normalization of miRNA expression in neuroendocrine tumors of the ileum and pancreas**. *Endocrine* 2014, **46**(2):226-230.
71. Finn RM, Ellard K, Eirin-Lopez JM, Ausio J: **Vertebrate nucleoplasmin and NASP: egg histone storage proteins with multiple chaperone activities**. *FASEB J* 2012, **26**(12):4788-4804.
72. Nagatomo H, Kohri N, Akizawa H, Hoshino Y, Yamauchi N, Kono T, Takahashi M, Kawahara M: **Requirement for nuclear autoantigenic sperm protein mRNA expression in bovine preimplantation development**. *Anim Sci J* 2016, **87**(3):457-461.
73. Shang Y, Wang H, Jia P, Zhao H, Liu C, Liu W, Song Z, Xu Z, Yang L, Wang Y *et al*: **Autophagy regulates spermatid differentiation via degradation of PDLIM1**. *Autophagy* 2016, **12**(9):1575-1592.
74. Yin J, Ni B, Tian ZQ, Yang F, Liao WG, Gao YQ: **Regulatory effects of autophagy on spermatogenesis**. *Biol Reprod* 2017, **96**(3):525-530.
75. Chen J, Shishkin AA, Zhu X, Kadri S, Maza I, Guttman M, Hanna JH, Regev A, Garber M: **Evolutionary analysis across mammals reveals distinct classes of long non-coding RNAs**. *Genome Biol* 2016, **17**:19.
76. Wiberg RA, Halligan DL, Ness RW, Necsulea A, Kaessmann H, Keightley PD: **Assessing Recent Selection and Functionality at Long Noncoding RNA Loci in the Mouse Genome**. *Genome Biol Evol* 2015, **7**(8):2432-2444.
77. Zhen Y, Andolfatto P: **Methods to detect selection on noncoding DNA**. *Methods Mol Biol* 2012, **856**:141-159.
78. Bleiweiss R: **Ultraviolet plumage reflectance distinguishes sibling bird species**. *Proceedings of the National Academy of Sciences of the United States of America* 2004, **101**(47):16561.

79. Bajer K, Molnár O, Török J, Herczeg G: **Female European green lizards (Lacerta viridis) prefer males with high ultraviolet throat reflectance**. *Behavioral Ecology and Sociobiology* 2010, **64**(12):2007-2014.
80. Bajer K, Molnár O, Török J, Herczeg G: **Ultraviolet nuptial colour determines fight success in male European green lizards (Lacerta viridis)**. *Biology Letters* 2011, **7**(6):866-868.
81. Averbeck M, Gebhardt CA, Voigt S, Beilharz S, Anderegg U, Termeer CC, Sleeman JP, Simon JC: **Differential Regulation of Hyaluronan Metabolism in the Epidermal and Dermal Compartments of Human Skin by UVB Irradiation**. *Journal of Investigative Dermatology* 2007, **127**(3):687-697.
82. Kurdykowski S, Mine S, Bardey V, Danoux L, Jeanmaire C, Pauly G, Brabencova E, Wegrowski Y, Maquart FX: **Ultraviolet-B irradiation induces differential regulations of hyaluronidase expression and activity in normal human keratinocytes**. *Photochem Photobiol* 2011, **87**(5):1105-1112.
83. Urrutia R: **KRAB-containing zinc-finger repressor proteins**. *Genome Biol* 2003, **4**.
84. Wolfe SA, Nekludova L, Pabo CO: **DNA recognition by Cys2His2 zinc finger proteins**. *Annu Rev Biophys Biomol Struct* 2000, **29**.
85. Englbrecht CC, Schoof H, Böhm S: **Conservation, diversification and expansion of C2H2 zinc finger proteins in the Arabidopsis thaliana genome**. *BMC Genomics* 2004, **5**(1):39.
86. Emerson RO, Thomas JH: **Adaptive evolution in zinc finger transcription factors**. *PLoS Genet* 2009, **5**(1):e1000325.
87. Nowick K, Fields C, Gernat T, Caetano-Anolles D, Kholina N, Stubbs L: **Gain, loss and divergence in primate zinc-finger genes: a rich resource for evolution of gene regulatory differences between species**. *PLoS One* 2011, **6**(6):e21553.
88. Rieseberg LH: **Chromosomal rearrangements and speciation**. *Trends in Ecology & Evolution* 2001, **16**(7):351-358.
89. Kirkpatrick M, Barton N: **Chromosome inversions, local adaptation and speciation**. *Genetics* 2006, **173**(1):419-434.
90. Böhme MU, Schneeweiß N, Fritz U, Schlegel M, Berendonk TU: **Small edge populations at risk: genetic diversity of the green lizard (Lacerta viridis viridis) in Germany and implications for conservation management**. *Conservation Genetics* 2007, **8**(3):555-563.
91. Joger U, Fritz U, Guicking D, Kalyabina-Hauf S, Nagy ZT, Wink M: **Phylogeography of western Palaearctic reptiles – Spatial and temporal speciation patterns**. *Zoologischer Anzeiger - A Journal of Comparative Zoology* 2007, **246**(4):293-313.
92. Weigert A, Helm C, Meyer M, Nickel B, Arendt D, Hausdorf B, Santos SR, Halanych KM, Purschke G, Bleidorn C *et al*: **Illuminating the base of the annelid tree using transcriptomics**. *Mol Biol Evol* 2014, **31**(6):1391-1401.
93. Kircher M, Sawyer S, Meyer M: **Double indexing overcomes inaccuracies in multiplex sequencing on the Illumina platform**. *Nucleic Acids Res* 2012, **40**(1):e3.
94. Meyer M, Kircher M: **Illumina sequencing library preparation for highly multiplexed target capture and sequencing**. *Cold Spring Harb Protoc* 2010, **2010**(6):pdb prot5448.
95. Nawrocki EP, Eddy SR: **Infernal 1.1: 100-fold faster RNA homology searches**. *Bioinformatics* 2013, **29**(22):2933-2935.
96. Nawrocki EP, Burge SW, Bateman A, Daub J, Eberhardt RY, Eddy SR, Floden EW, Gardner PP, Jones TA, Tate J *et al*: **Rfam 12.0: updates to the RNA families database**. *Nucleic Acids Res* 2015, **43**(Database issue):D130-137.
97. Lowe TM, Eddy SR: **tRNAscan-SE: a program for improved detection of transfer RNA genes in genomic sequence**. *Nucleic Acids Res* 1997, **25**.
98. Bartschat S, Kehr S, Tafer H, Stadler PF, Hertel J: **snoStrip: a snoRNA annotation pipeline**. *Bioinformatics* 2014, **30**(1):115-116.
99. Jorjani H, Kehr S, Jedlinski DJ, Gumienny R, Hertel J, Stadler PF, Zavolan M, Gruber AR: **An updated human snoRNAome**. *Nucleic Acids Res* 2016, **44**(11):5068-5082.

100. Taft RJ, Glazov EA, Lassmann T, Hayashizaki Y, Carninci P, Mattick JS: **Small RNAs derived from snoRNAs**. *RNA* 2009, **15**(7):1233-1240.
101. Haas BJ, Papanicolaou A, Yassour M, Grabherr M, Blood PD, Bowden J, Couger MB, Eccles D, Li B, Lieber M *et al*: **De novo transcript sequence reconstruction from RNA-Seq: reference generation and analysis with Trinity**. *Nature protocols* 2013, **8**(8):10.1038/nprot.2013.1084.
102. Hertel J, Stadler PF: **The Expansion of Animal MicroRNA Families Revisited**. *Life (Basel)* 2015, **5**(1):905-920.
103. Smit A, Hubley R: **RepeatModeler Open-1.0**. 2008-2015.
104. Smit A, Hubley R, Green P: **RepeatMasker Open-4.0**. 2013-2015.
105. Li H: **Aligning sequence reads, clone sequences and assembly contigs with BWA-MEM**. *arXiv:13033997* 2013.
106. Garrison E, Marth G: **Haplotype-based variant detection from short-read sequencing**. *arXiv preprint* 2012, **arXiv:1207.3907**([q-bio.GN]*).
107. Lohse K, Harrison RJ, Barton NH: **A general method for calculating likelihoods under the coalescent process**. *Genetics* 2011, **189**(3):977-987.
108. Nurnberger B, Lohse K, Fijarczyk A, Szymura JM, Blaxter ML: **Para-allopatry in hybridizing fire-bellied toads (Bombina bombina and B. variegata): Inference from transcriptome-wide coalescence analyses**. *Evolution* 2016, **70**(8):1803-1818.
109. Caughley G: **Parameters for Seasonally Breeding Populations**. *Ecology* 1967, **48**(5):834-839.
110. Elbing K: **Zur Wiederbesiedlung brandgeschädigter Teilhabitate durch Smaragdeidechsen (Lacerta viridis)**. *Salamandra* 2000, **36**(3):175-184.
111. Saint Girons H, Castanet J, Bradshaw D, Baron J-P: **Démographie comparée de deux populations françaises de Lacerta viridis (Laurenti, 1768)**. *Journal of Ecology* 1989, **44**(4):361-386.
112. Rykena S, Nettmann HK: **Lacerta viridis (Laurenti, 1768) – Smaragdeidechse**, vol. 2; 1984.
113. Tollis M, Ausubel G, Ghimire D, Boissinot S: **Multi-Locus Phylogeographic and Population Genetic Analysis of Anolis carolinensis: Historical Demography of a Genomic Model Species**. *PLoS One* 2012, **7**(6):e38474.
114. Mohiyuddin M, Mu JC, Li J, Bani Asadi N, Gerstein MB, Abyzov A, Wong WH, Lam HY: **MetaSV: an accurate and integrative structural-variant caller for next generation sequencing**. *Bioinformatics* 2015, **31**(16):2741-2744.
115. Chen K, Wallis JW, McLellan MD, Larson DE, Kalicki JM, Pohl CS, McGrath SD, Wendl MC, Zhang Q, Locke DP *et al*: **BreakDancer: an algorithm for high-resolution mapping of genomic structural variation**. *Nat Methods* 2009, **6**(9):677-681.
116. Abyzov A, Urban AE, Snyder M, Gerstein M: **CNVnator: an approach to discover, genotype, and characterize typical and atypical CNVs from family and population genome sequencing**. *Genome Res* 2011, **21**(6):974-984.
117. Ye K, Schulz MH, Long Q, Apweiler R, Ning Z: **Pindel: a pattern growth approach to detect break points of large deletions and medium sized insertions from paired-end short reads**. *Bioinformatics* 2009, **25**(21):2865-2871.
118. Xie C, Tammi MT: **CNV-seq, a new method to detect copy number variation using high-throughput sequencing**. *BMC Bioinformatics* 2009, **10**:80-80.
119. Kent WJ, Baertsch R, Hinrichs A, Miller W, Haussler D: **Evolution's cauldron: duplication, deletion, and rearrangement in the mouse and human genomes**. *Proc Natl Acad Sci U S A* 2003, **100**(20):11484-11489.
120. Blanchette M, Kent WJ, Riemer C, Elnitski L, Smit AF, Roskin KM, Baertsch R, Rosenbloom K, Clawson H, Green ED *et al*: **Aligning multiple genomic sequences with the threaded blockset aligner**. *Genome Res* 2004, **14**(4):708-715.
121. Hickey G, Paten B, Earl D, Zerbino D, Haussler D: **HAL: a hierarchical format for storing and analyzing multiple genome alignments**. *Bioinformatics* 2013, **29**(10):1341-1342.
122. Harris R: **Improved pairwise alignment of genomic DNA.** . *Ph.D. Thesis.* The Pennsylvania State University.; 2007.

123. Walter Costa MB, Höner zu Siederdissen C, Stadler PF, Nowick K: **SSS-test: a novel test for detecting selection on the secondary structures of non-coding RNAs. (submitted)**. *Nucleic Acids Res* 2017.

124. Lorenz R, Bernhart SH, Höner zu Siederdissen C, Tafer H, Flamm C, Stadler PF, Hofacker IL: **ViennaRNA Package 2.0**. *Algorithms for Molecular Biology* 2011, **6**(1):26.

125. Lange SJ, Maticzka D, Mohl M, Gagnon JN, Brown CM, Backofen R: **Global or local? Predicting secondary structure and accessibility in mRNAs**. *Nucleic Acids Res* 2012, **40**(12):5215-5226.

126. Yates A, Akanni W, Amode MR, Barrell D, Billis K, Carvalho-Silva D, Cummins C, Clapham P, Fitzgerald S, Gil L *et al*: **Ensembl 2016**. *Nucleic Acids Res* 2016, **44**(D1):D710-716.

127. Hongo JA, de Castro GM, Cintra LC, Zerlotini A, Lobo FP: **POTION: an end-to-end pipeline for positive Darwinian selection detection in genome-scale data through phylogenetic comparison of protein-coding genes**. *BMC Genomics* 2015, **16**:567.

128. Yang Z: **PAML 4: phylogenetic analysis by maximum likelihood**. *Mol Biol Evol* 2007, **24**(8):1586-1591.

Figure Legends

Figure 1. Thirteen different demographic scenarios were fitted. The models M1.1, M2.1 and M3.1 are strict divergence without gene flow; M1.2, M2.2 and M3.2 allow for post-divergence gene flow from *L.viridis* to *L. bilineata*; M1.3, M2.3 and M3.3 assume gene flow in the reverse direction i.e. (from *L. bilineata* to *L. viridis*). The models M4.2 and M5.2 allow for discrete admixture from *L. viridis* to *L. bilineata* and models M4.3 and M5.3 assume the admixture in the reverse direction (from *L. bilineata* to *L. viridis*). The effective population size is either assumed to be identical between both species and their ancestor (class M1.*) or one of the species has a different effective population size compared to the other species and ancestor (classes M2.*-5.*).

Figure 2. Total counts and length ranges (in bp) of genomic rearrangements of SVs between L. viridis and L. bilineata. The counts are represented by bars and length ranges by whiskers (y-axis is log10-scaled). The rearrangements plotted are categorized into deletions (DEL), duplications (DUP), insertions (INS) and inversions (INV).

Figure 3. The folded blockwise site frequency spectrum (bSFS). The variation in alleles represented by different colours (the ancestral state showed in red). Given a single genealogy (a diploid genome from two populations can form six possible genealogies), each block contains four mutation types: i) unique heterozygous sites in L. bilineata, ii) unique heterozygous sites in L. viridis, iii) shared heterozygous sites between L. viridis and L. bilineata or iv) homozygous sites which are different between L. viridis and L. bilineata i.e. homozygous fixed differences. The bSFS (spectrum of SFS) has been calculated by counting the number of occurrences of each SFS.

Tables

Table 1. The ΔAIC of the best model (i.e IM 2 B(x)–>V) compared to the other scenarios. To lower the effect of linkage between blocks we adjusted the AIC values of each model by only sampling every 1000 blocks. The best model is highlighted in bold. Note: ADM - Isolation with discreet admixture; IM - Isolation with migration and DIV - Strict divergence without gene flow.

Model ID	Model type	ΔAIC
M5.3	ADM 2B(x)–>V	-47.2
M3.2	ADM 2V (x)–>B	-141
M4.3	ADM 2B–>V (x)	-47.2
M3.2	ADM 2V –>B(x)	-141

**M3.3**	**IM 2B(x)–>V**	**0**
M3.2	IM 2V(x)–>B	−77.9
M3.1	DIV 2 b	−1380
M2.3	IM 2B–>V (x)	−32.9
M2.2	IM 2V –>B(x)	−86.5
M2.1	DIV 2	−1140
M1.3	IM 1B–>V	−487
M1.2	IM 1V –>B	-128
M1.1	DIV 1	−1380

1029

Figure 1

Click here to access/download
Supplementary Material
Figure_1.svg

Figure 2

Click here to access/download
Supplementary Material
Figure_2.svg

Figure 3

Click here to access/download
Supplementary Material
Figure_3.svg

Click here to access/download
Supplementary Material
Lacertids_Manuscript-Gigascience_Supplement_FINAL.pdf

Supplementary File 2

Click here to access/download
Supplementary Material
Additional_File_2.pdf

Supplementary File 3

Click here to access/download
Supplementary Material
Additional_File_3.nb

Personal Cover
Click here to access/download;Personal Cover;Lacertids_Manuscript-Gigascience_Letter.pdf

UNIVERSITÄT LEIPZIG

Fakultät für Lebenswissenschaften
Institut für Biologie
Molekulare Evolution und Systematik der Tiere
Prof. Dr. Martin Schlegel

17. May 2018

Universität Leipzig, Institut für Biologie, Talstraße 33, 04103 Leipzig

Dear *Dr. Scott Edmunds*,

We enclose our manuscript "*Divergent evolution in the genomes of closely-related lacertids, Lacerta viridis and L. bilineata and implications for speciation*" for your consideration.

Lizards are valuable resources to study evolution in higher vertebrates due to their well-known phylogeographies and adaptive radiation. The phylogeography of the European green lizards (*Lacerta viridis* complex) in particular has been studied since three decades through ecological experiments and molecular markers. The *L. viridis* complex was recently delineated into two separate species, *L. viridis* and *L. bilineata*, owing to the reproductive barriers between their progeny. However, factors contributing to their divergence are unknown and their species status is still debatable due to the absence of morphological variation.

We present the genomes of *L. viridis* and *L. bilineata*, the first in *Lacertidae* family through *de novo* assemblies using Illumina and PacBio sequencing technologies. Transcriptomic data of multiple tissues from the same individuals were used for annotation and detecting signs of selection. We conducted genome-wide analyses mainly involving genomic variation, rearrangements, changes in non-coding elements and species-specific adaptions. We implemented the blockwise site frequency spectrum to study lacertid demographic histories and estimated their split around 2.7-3.05 Mya with gene flow in the direction from *L. viridis* to *L. bilineata*.

We found adaptation of cutaneous UV-B responsive genes as major drivers of preferential mating in lacertids. Species specific diversification of the non-coding repertoire; transcription factors divergence; adaptation of genes related to behavior, neuronal and reproductive development; association of positively selected genes with inversions as contributors to reproductive isolation. We provide first insights into the genomes and population histories of European green lizards pivotal for their conservation as well as important resources for future work on speciation.

We hope that you agree that our manuscript is of interest to the broad readership of *Gigascience* since it overlaps with multiple themes of your esteemed journal including comparative genomics, evolution and ecology.

All the authors declare no competing interests and approve the manuscript for submission. We confirm that the content has not been published or submitted elsewhere. We look forward to hearing from you.

With best regards,
Rohit Kolora, *Peter F Stadler* and *Martin Schlegel*

Universität Leipzig
Fakultät für Lebenswissenschaften
Institut für Biologie
Molekulare Evolution und Systematik der Tiere
Talstraße 33
04103 Leipzig

Telefon
+49 341 97-36725

Fax
+49 341 97-36789

E-Mail
schlegel@rz.uni-leipzig.de

Web
http://home.uni-leipzig.de/agspzoo/

Postfach intern
220001

Kein Zugang für elektronisch signierte sowie für verschlüsselte elektronische Dokumente
