# Supplementary material for: Divergent evolution in the genomes of closely related lacertids, Lacerta viridis and L. bilineata, and implications for speciation
Source: Gigascience. 2018 Dec 10;8(2):giy160. doi: 10.1093/gigascience/giy160 (PMC6381762; doi:10.1093/gigascience/giy160)
Supplement: giy160_Supplemental_Files [file giy160_supplemental_files.zip › Additional_File_2.pdf]

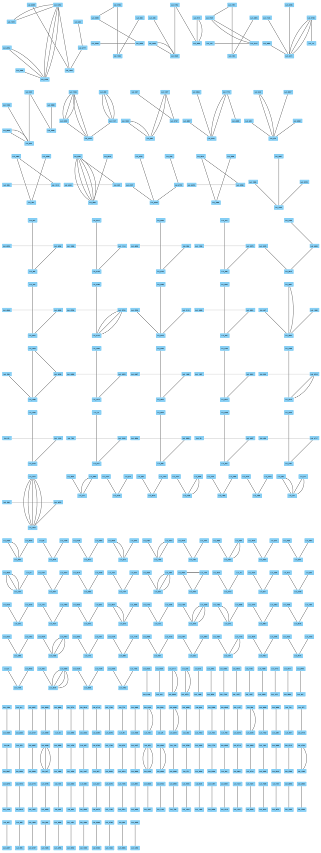

Figure: Representation of unordered contig clusters created from the synteny information between L. viridis and L. bilineata (minimum contig length of 1 Mbp). Each cluster represents contigs positioned on the same chromosome. The nodes represent contigs of L. viridis while the edges are from L. bilineata.
