# Supplementary material for: Divergent evolution in the genomes of closely related lacertids, Lacerta viridis and L. bilineata, and implications for speciation
Source: Gigascience. 2018 Dec 10;8(2):giy160. doi: 10.1093/gigascience/giy160 (PMC6381762; doi:10.1093/gigascience/giy160)
Supplement: giy160_Supplemental_Files [file giy160_supplemental_files.zip › Lacertids_Manuscript-Gigascience_Supplement_Proofs.pdf]

Table of Contents

Supplementary Methods **2**

Data filtering and error correction 2

de novo genome assembly and quality metrics 2

Gene prediction, transcript assembly and annotation 3

Estimation of mutation rate from coding sequences 4

Z-chromosome baiting 4

Whole genome alignments and synteny 5

Filtering predicted SVs 5

Prediction of conserved sites across tetrapods 5

Lineage-specific selection and transcription factor evolution 6

HOX clusters and UV-vision 6

Effect of rearrangements and selection 7

Supplementary Information **8**

Lacertids and their genome qualities 8

Genome annotation and evolution of noncoding elements 11

Characteristics of the Z-chromosome contigs 16

Gene flow between lacertids 17

Accelerated sequence evolution 18

Genome variation through SVs or rearrangements 18

Structural selection of ncRNA 20

Adaptation to UV-exposure in the skin 22

Possible biological implications of selection 22

Evolution of transcription factors and alternative splicing variation 28

Evolutionary divergence due to rearrangements 29

References **30**

Supplementary Methods

SM-1. Data filtering and error correction

Filtering: The raw Illumina reads were processed to remove adaptors and indices using leeHom v1.1.5 (leeHom, RRID:SCR_002710) [1] resulting in reads with peak-lengths of 96bp. The genome coverage obtained through Illumina sequencing was estimated to be 34-fold for *L. viridis* (51Gb output) and 37-fold for *L. bilineata* (56Gb output). Overlapping paired-end reads were merged using FLASH v1.2.11 (FLASH, RRID:SCR_005531) [2] with a minimum overlap of 33 and an error rate fraction of 0.05. Read-filtering was employed with a minimum base quality of 30 towards ends of the reads, removal of reads with ambiguous bases (Ns) and discarding reads smaller than 60bp and 35bp using NGSQC v2.3.3 (NGSQC, RRID:SCR_005459) [3] for genome and transcriptome data respectively. For the PacBio reads, adaptors were removed using BLASR v1.3.1 (BLASR, RRID:SCR_000764) [4] as included in SMRT Portal 2.3.0 (SMRT-Analysis, RRID:SCR_002942). The sub-reads were filtered to retain those with a minimum length of 50bp and minimum read quality of 0.75. After these filters, 18.9 Gb and 15.6 Gb of PacBio sub-read bases were retained for *L. viridis* and *L. bilineata* respectively.

Error-correction: The genomic Illumina reads were normalized with BBTools v38.08 [5] to obtain a coverage of 2X-40X. The PacBio sub-reads were corrected with Proovread v2.13.4 [6] using the normalized Illumina reads (PacBio error-profile of 10% insertions, 5% deletions and 1% substitutions).

SM-2. *de novo* genome assemblies and their quality metrics

The genome sizes of the two lacertid genomes were calculated with a k-mer (sub-strings of length k in sequencing data) of 17 and compared to the animal genome size database [7] (1.53-1.62 Gbp, c-value of 1.56-1.65). Unitig assemblies (high confidence contigs split at repeat boundaries) were generated with the ABySS assembler V1.9 (ABySS, RRID:SCR_010709) [8] with the filtered Illumina reads with a k-mer size of 51 and minimum k-mer coverage of 3. A hybrid genome assembly was constructed using Illumina unitigs and intermediate error corrected PacBio sub-reads (without splitting chimeric PacBio reads) using DBG2OLC [9]. A k-mer size of 17, adaptive k-mer matching threshold of 0.01, minimum overlap score of 20 (based on alignments between compressed reads and matching k-mers) and chimera removal produced the most contiguous backbone assemblies. The consensus sequence of the backbone

assembly was created using Sparc consensus caller [10] with two iterations to remove indel errors mainly from PacBio data. The final assemblies were polished (removal of remaining indel errors) with three iterations of Pilon v1.16 (Pilon , RRID:SCR_014731) [11] utilizing the paired-end Illumina and error corrected PacBio (split at chimeric intervals). Genome assembly quality and completeness were predicted with gVolante [12] based on core vertebrate genes (CVGs) [13]. Genome metrics, such as number of sequences, genome size, average and median size of the sequences, GC content, N50 size (length of the shortest contig such that the sum of contigs of qua length or longer is at least 50% of the total length of all contigs), L50 (smallest number of contigs whose length sum produces N50) and genome completeness of *L. viridis* and *L. bilineata,* were compared to other lizards (*A. carolinensis* and *G. japonicus*).

The CpG islands in the genome were detected using the newcpgreport tool of the EMBOSS package v6.6.0 (EMBOSS CpGPlot/CpGReport/Isochore, RRID:SCR_007254) [14] with a sliding-window of 100, minimum GC fraction of 0.5, minimum observed-to-expected CpG ratio of 0.6 and minimum island length of 200 bases.

SM-3. Gene prediction, transcript assembly and annotation

The filtered RNAseq reads were mapped to the respective genome assemblies using bowtie2 v2.2.5 (Bowtie 2, RRID:SCR_016368) [15] and the genome annotation was performed with the BRAKER pipeline [16]. InterProScan v5.18-57.0 (InterProScan, RRID:SCR_005829) [17] was used to predict the pFAM domains (Pfam, RRID:SCR_004726) [18], InterPro domains (InterPro, RRID:SCR_006695) [19] and the PANTHER families (PANTHER, RRID:SCR_004869) [20] of the proteins encoded by the coding sequences. Tests of enrichment for specific functional categories were performed using the over-representation tests of PantherDB [21] (release 20170413) along with multiple testing through Bonferroni correction.

As the *ab initio* annotation gene models were fragmented, mRNA transcripts were also used to annotate the genome of each species, separately. The transcripts were *de novo* assembled using the Trinity assembly pipeline for each tissue separately [22]. The annotation and down-stream analysis of the transcript assemblies from the different tissues was carried out with the Trinotate pipeline v2.0 [23]. Similarity search was conducted with BLAST+ v2.2.29 [24, 25] against the uniprot (UniProt, RRID:SCR_002380) [26] and swissprot databases. Protein domains were annotated by searching the pFAM database [18] with HMMER3 (Hmmer, RRID:SCR_005305) [27]. Gene ontology (Gene Ontology, RRID:SCR_002811) prediction was performed using the GO database [28]. Signal peptides and trans-membrane regions were predicted with SignalP

4.0 (SignalP, RRID:SCR_015644) [29] and TMHMM 2.0 (TMHMM Server, RRID:SCR_014935) [30] respectively. The *de novo* transcripts were aligned against their respective genomes with GMAP/GNSAP v2016-08-24 aligner (GMAP, RRID:SCR_008992) [31]. In order to remove redundancy from the tissue-specific transcript assemblies, only the longest coding transcripts of all tissues which aligned on the genome without chimeras were retained for annotation. This process allowed to predict the contiguous boundaries of the fragmented genes annotated using *ab initio* models. Final annotations were generated by merging overlapping fragmented genes from different models and retaining those with transcript support.

The putative high identity gene paralogs were separately identified for each species by using Proteinortho V5 [32] allowing for at most 95% identity, clustering of additional hits with a similarity of 0.99 with the best hit in each group and e-value filtering of 1e-10. For each lacertid, only the coding regions that were part of the same homologous group but originated from different genomic coordinates were assumed to be paralogs. The introns were annotated based on the spliced coding sequences of the transcripts that mapped onto the genome. UTRs were annotated based on the transcript boundaries upstream of the first and downstream of the last exon supported by mapped transcripts.

SM-4. Estimating mutation rate from coding sequences

Divergence between the two lacertid species was estimated by calculating the Ka (non-synonymous substitution rate) and Ks (synonymous substitution rate) for the orthologous coding sequences detected by ProteinOrtho [32]. The orthologs were pair-wise aligned with MACSE [33] and the Ka-Ks values were computed using kaks_calculator 2.0 [34] with model averaging (MA) by engaging different substitution models to estimate average parameters across models. The average synonymous and non-synonymous substitution rates were calculated from the orthologous pairs with valid Ka and Ks (Fisher's exact test, $p \leq 0.05$). The lineage-specific mutation rate in the ancestral lacertid lineage was estimated based on a generation time (g) of 3.5 years (see methods) and the synonymous substitution rate (dS) of 0.021 similar to the estimation in birds [35]. The most recent common ancestor (TMRCA) between *L. viridis* and *L. bilineata* was 3 million years [36].

$$\text{Mutation rate } (\mu) = \frac{(\text{synonymous substitution rate (dS)}/2)}{\frac{\text{TMRCA}}{\text{generation time (g)}}}$$

On substituting these values, the ancestral lacertid lineage mutation rate was estimated as 1 x 10^{-9}.

SM-5. Z-chromosome baiting

The sex linked contigs were baited based on existing knowledge of Z- chromosome specific genes in lacertid lizards [37, 38]. The synteny information between *L. viridis* and *L. bilineata* was used to extract Z-specific contigs in both species. GMAP [31] was used to map the transcripts of the query genes with tolerance for frame-shifts.

SM-6. Whole genome alignments and synteny in lacertids

Whole genome alignments between the two lacertids were generated with the blastz-synteny pipeline from UCSC [39]. High scoring LASTZ alignments with a score-threshold of 5000, step-size of 9, inner threshold of 3000 followed by chaining and netting was implemented. Syntenic blocks were used in the detection of genomic rearrangements. The collinear blocks with conserved gene orders between the lacertid genomes were identified by extracting syntenic blocks (at least 1 kb) observed in both directions (*L. viridis* reference to *L. bilineata* query and vice-versa), filtering out regions overlapping with larger rearrangements and retaining those with at least three genes in each block. Our defined collinear blocks totaled to a length of 55.3 Mbp of the *L. viridis* genome.

SM-7. Filtering predicted SVs by quality

We considered the genomic rearrangements that satisfies one of the following conditions: (1) high quality (PASS tag) in MetaSV, (2) high quality precise events in Sniffles, (3) verified events detected through halBranchMutations or (4) simple indels from syntenic blocks (clustered gaps in one genome but no gap in another) or inversions indicated by strand changes in successive syntenic blocks (for example +/-/+ or -/+/-). The predicted rearrangements that overlapped were merged according to their type (insertions, inversions, deletions and duplications). These list of high quality rearrangements were then filtered restricting to those non-overlapping with the control set. The control set were rearrangements detected through MetaSV by aligning Illumina reads (*L. viridis* only due to higher contiguity) and detected through PacBio reads using Sniffles of each lacertid genome against itself (both *L. viridis* and *L. bilineata* reads against their respective genome assemblies).

SM-8. Prediction of conserved sites across Tetrapods and accelerated evolution

Multiple genome alignments were built with *L. viridis* as the reference and the queries from the chromosomes or contigs of *L. bilineata*, *Homo sapiens* (hg19), *Gallus gallus* (galGal3), *Xenopus tropicalis* (xenTro3), *Alligator mississippiensis* (allMis1) and *Anolis carolinensis* (anoCar2) using tba-roast and MultiZ [40]. Single-coverage alignments (using single_cov2) were generated where the query and target regions are represented once. The conserved and the nonconserved models were separately estimated from the training data. Conservation scores for the *L. viridis* genome were computed based on these single-coverage multi-genome alignments for every base with phylop [41] from the PHAST v1.3 package (PHAST, RRID:SCR_003204) [42]. The phyloP scores were generated with base-by-base option using the SCORE method with CONACC mode under a neutral background model. To test for accelerated evolution between the two lacertids, the features from the annotation of the genes (gene boundaries, introns and exons) were used to generate phyloP scores separately (features option, SCORE method, CONACC mode). The conserved sites were also estimated for sauropsids branches alone by excluding *H. sapiens* and *X. tropicalis* from the tetrapod tree of the initial analysis.

SM-9. Lineage-specific selection and transcription factor evolution in lacertids

The conserved genomic elements and those under varying levels of purifying selection were predicted with dless tool [43]. phyloFit was used to generate the tree models from the multiple sequence alignments (MSA) of lacertids with five outgroups (*H. sapiens*, *G. gallus*, *X. tropicalis*, *A. mississippiensis* and *A. carolinensis*) and indelHistory of PHAST package [44] was used to predict indels across species. dless enabled the identification of elements that were conserved in the lacertid ancestor compared to outgroups, conserved or ceased to be under selection in either of the lacertid species. The gain events in this model indicate conservation of genomic elements in a particular lineage or sub-tree since their split from ancestor while loss events indicate loss of selection on a particular branch.

The orthologous KRAB zinc-finger proteins (ZNFs) between lacertids with the DNA-binding “C[2]C[12]H[3]H” domains were aligned using MUSCLE v3.7 (MUSCLE, RRID:SCR_011812) [45]. These alignments were queried for changes in the amino acids adjacent to or in the C2H3 domains (amino acid position 5-12).

SM-10. HOX clusters and adaptation to UV-vision

The HOX-genes were identified based on orthologs information (using blast) between lacertids and *A. carolinensis* [46]. The distance (in bp) between the HOX13 gene and the HOXB cluster of lacertids was compared to *A. carolinensis* and the conserved miRNA orthologs in the HOX-cluster were identified.

The five opsin genes of *A. carolinensis* (22 transcript sequences from ensembl) were compared to the opsins in lacertids. The SWS1 opsin was missing in the transcript data, so the transcript was baited using the transcript and coding sequence of *A. carolinensis* employing GMAP [31]. The absence of SWS1 opsin in the lacertid transcripts can be attributed to its tissue specific expression in the eye, for which no transcriptome libraries were generated. The amino acid sequence of the SWS1 opsin, which is responsible for UV-vision in birds, snakes and lizards [47, 48], was compared to the following sauropsids: *Anolis carolinensis, Gekko gecko, Psittacus erithacus, Python regius, Xenopeltis unicolor, Thamnophis proximus, Uta stansburiana, Taeniopygia guttata, Ficedula albicollis* and *Xenopus tropicalis* to study its evolution.

SM-11. Effect of rearrangements and selection

The genes with $\omega>1$ ($p<0.05$) detected through branch-site model of codeml were considered as positively selected (PSGs) while the remaining orthologous genes without signs of positive-selection were considered neutral or negatively selected genes (NPSGs). Since Fisher's test is conditioned on both margins, we tested for association of the different rearrangement categories with positively selected genes with Boschloo's exact test (unconditional exact-test). Exact package [49] was used to perform the Boschloo's test assuming a binomial distribution and unconditioned row margins. To address the most prominent category of rearrangement that occurs with positive selection, we calculated the effect of each category separately against a) other categories of rearrangements b) against regions without rearrangements and c) collinear regions of the genomes.

Supplementary Information

SI-1. Lacertid genome qualities and synteny conservation to *A. carolinensis*

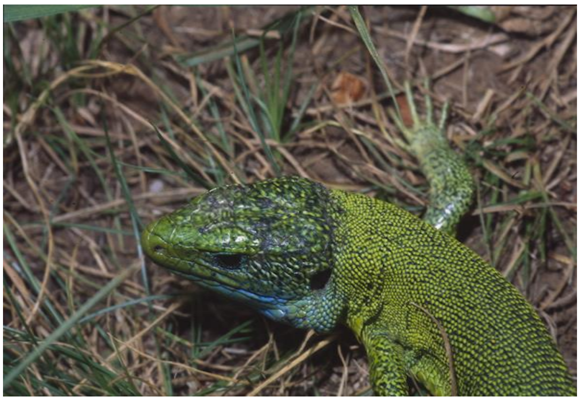

(A) *Lacerta bilineata*

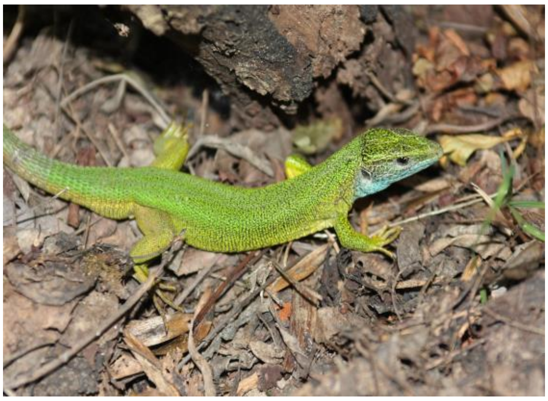

(B) *Lacerta viridis*

Figure S1. Two adult males (A) *L. bilineata* (close up of the head) and (b) *L. viridis* (whole body) observed in their natural habitat. The body sizes of the males are smaller than the females. There are no noticeable morphological differences between the adults, however juveniles between the two species show variation in throat color (not pictured).

Table S1. High quality of lacertids genomes in comparison to *A. carolinensis* and *G. japonicus*. Summary statistics are based on their, genome metrics contiguity and completeness. The genome completeness is predicted using CEGMA and BUSCA v2/v3 on CVGs [13]. bp – base pairs, CVGs – core vertebrate genes.

Genome metric	*Lacerta virdis* (contig-level)	*Lacerta bilineata* (contig-level)	*Anolis carolinensis* (scaffold-level)	*Gekko japonicas* (scaffold-level)
**Number of sequences**	4,737	7,510	6,457	191,500
**Total assembly size (Gbp)**	1.44	1.42	1.79	2.49
**Minimum sequence length (bp)**	1653	676	5000	200
**Maximum sequence length (Mbp)**	3.63	2.88	26.4	4.76
**Average sequence length (kbp)**	304	189	279	13
**Contig N50 size (kbp)**	663	368	80	21
**Scaffold N50 size (kbp)**	-	-	150642	684
**Contig L50**	661	1,150	6,217	33,312
**Scaffold L50**	-	-	5	963
**GC content (%)**	43.75	43.64	40.3	45
**CEGMA CVGs completeness %**	99.57	97.85	98.7	97.8

**BUSCO CVGs completeness % (complete)**	95.71	93.99	89.70	87.55
**BUSCO CVGs completeness % (complete+partial)**	97	97.85	93.99	96.14
**Number of CpG islands (% of genome)**	72,844 (1.7%)	65,950 (1.5%)	41,719 (0.8%)	102,284 (1.3%)

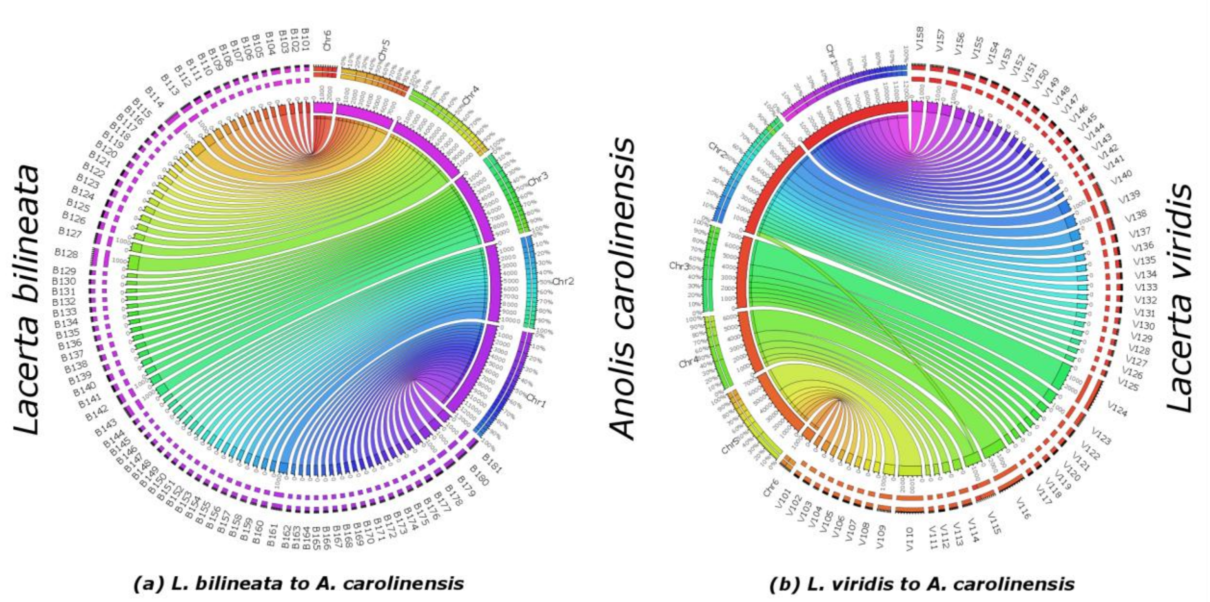

Figure S2. Syntenies of *L. bilineata* (a) and *L. viridis* (b) contigs (purple and red outer-most blocks respectively) with the *A. carolinensis* genome (other colors) plotted with the Circos visualizer. The contigs of *L. bilineata* are represented on the left (B) and contigs of *L. viridis* are on the right side (V) whereas the chromosomes of *A. carolinensis* are preceded by “Chr”. Note: The identifiers of the contigs are arbitrary and do not represent the actual contig names.

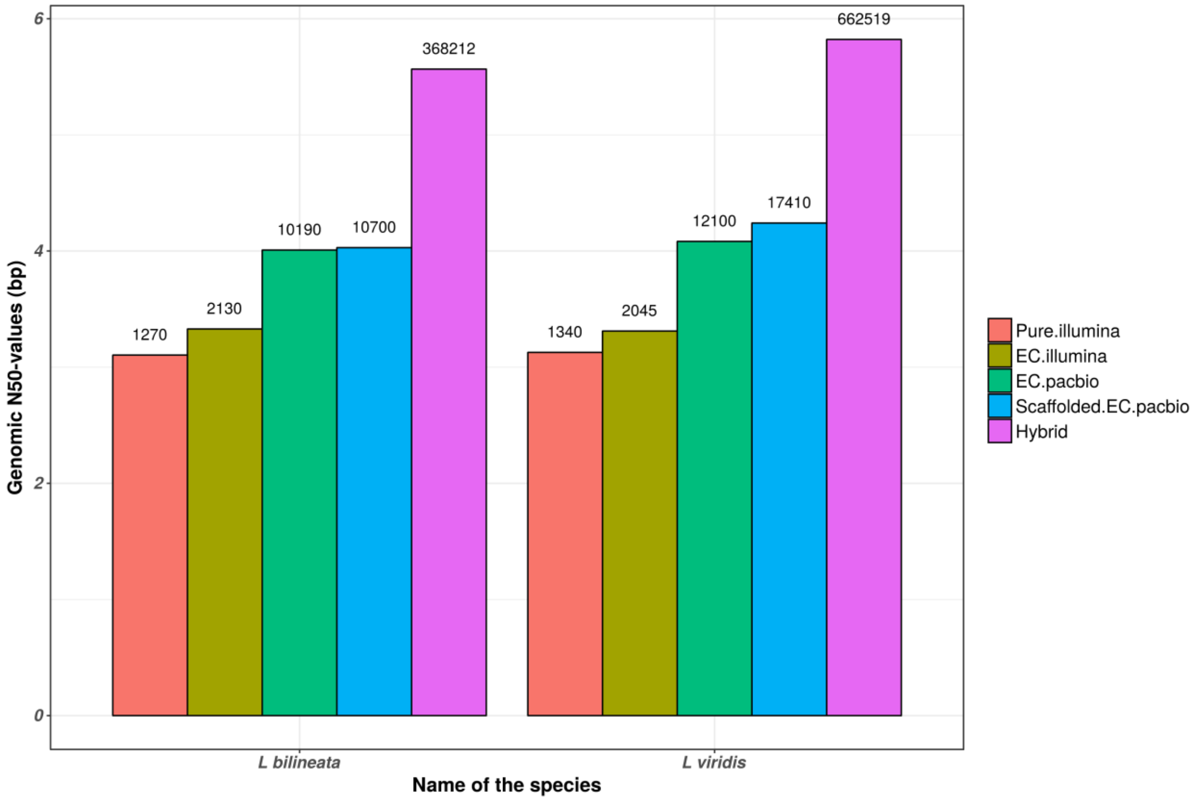

Figure S3. Contiguity of lacertid genomes (N50) in accordance with the employed sequencing methods and assembly strategies (y-axis is $\log_{10}$-scaled). E.C. – error corrected.

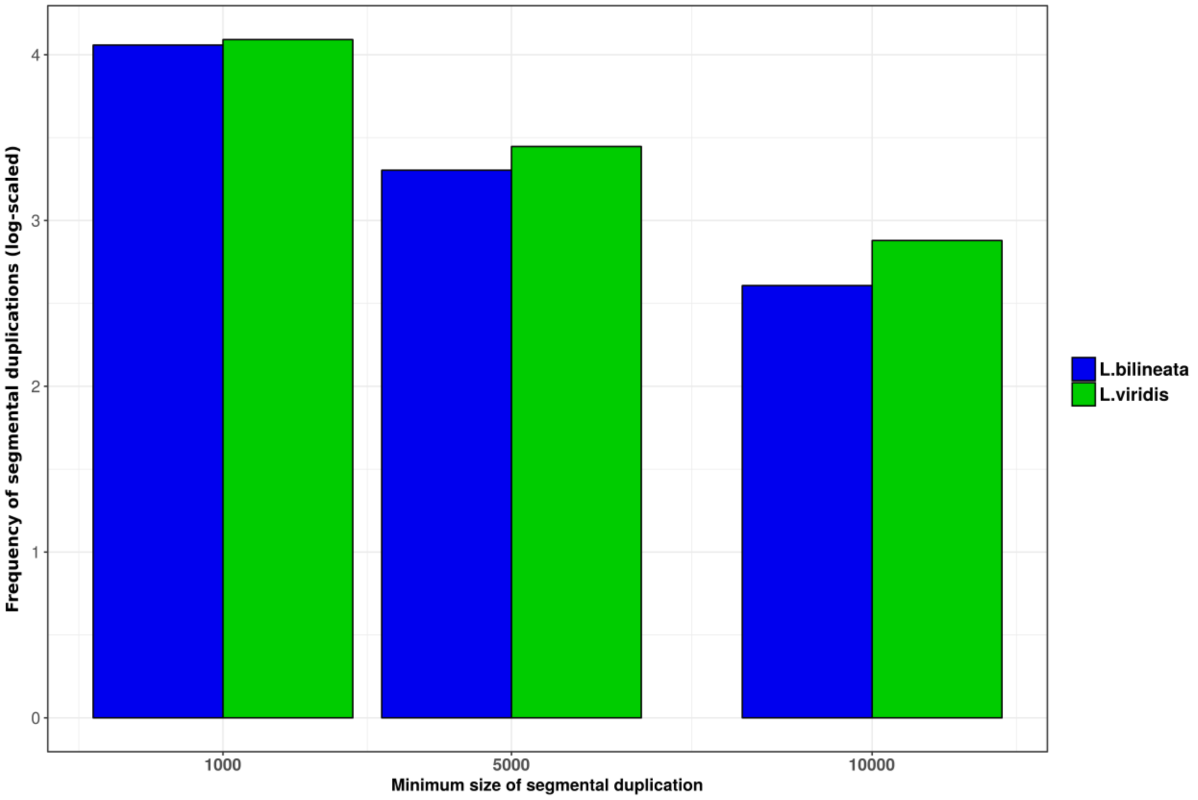

Figure S4. Segmental duplications in lacertids with at least 90 % identity. The minimum length of the duplication is denoted on the x-axis, y-axis is $\log_{10}$-scaled and denotes the frequency of duplications.

L. viridis contains more segmental duplications than *L. bilineata* (Figure S4), however this can be due to the higher fragmentation of the *L. bilineata* genome assembly. The two lacertid genomes had four HOX clusters with the complement of HOX genes expected from *A. carolinensis* [46]. Similar to *A. carolinensis*, the HOX13 gene was separated by almost 100kb from the rest of the HOXB cluster. The lacertid HOX clusters also contained the expected two mir-10 and three mir-196 paralogs. The distances between the HOX genes and the occurrence of miRNA paralogs were inspected manually.

SI-2. Genome annotation and evolution of noncoding and repeat elements

Table S2. Number of genes predicted based on *ab initio* models and transcript-based data. Only those supported by transcripts were included into the final annotations. EM – expectation maximization.

Species	AUGUSTUS models	GeneMark-EM models	Transcript models	Final #genes in the annotation
Lacerta viridis	38506	30251	22560	22156
Lacerta bilineata	54773	42970	25215	22491

We identified 10652 single-copy orthologs between the lacertids and 4334 in lizards (shared with *A. carolinensis, P. vitticeps, G. japonicus*). Lacertid specific paralogs were observed for RNA-binding proteins (CFDP2) and ribosomal proteins (RPL7A).

Interestingly, lacertids have twice as many tRNA genes as the other available reptilian genomes except *G. japonicus* (Figure S4). Both the number of functional and pseudo-tRNAs increased in lacertids and *G. japonicus*, compared to the other species in the paraphyletic group of reptiles. Outstanding are the high number of functional tRNA genes containing anticodons for Alanine, Lysine and Isoleucine. Highly abundant tRNAs are also found in the genomes of *G. japonicus* (including those of Alanine anticodon) and *A. mississippiensis* (several anticodons) (Figure S5). Such variations in functional and pseudo-tRNA gene numbers have also been observed in other eukaryotes [50]. Even though we did not observe structural selection differences for tRNA within the lacertids, there were three positively selected tRNAs in the lacertid ancestor (tRNA-Glu(CTC) and tRNA-Arg(CCT)).

We also identified 244 miRNAs in *L. bilineata* and 242 miRNAs in *L. viridis*, respectively. This is similar to miRNA counts in mammals but fewer than in *G. gallus* and *A. carolinensis* (Figure S6). While the total number of predicted snoRNA sequences in the two lacertids and A. carolinensis are the same (235), they belong to different numbers of snoRNA families. While representatives of families SNORA 17-43 and SNORA20 were detected in *L. viridis* (159 families), they could not be identified in *L. bilineata* (157 families) (Figure S7). There is an observed loss of 20 snoRNA families in the lacertids compared to *A. carolinensis* (179 families). Whether these losses are due to limitations in homology search or true evolutionary gene losses could not be resolved with the current data. A gain in snoRNA families in Therian mammals compared to other ncRNA might have led to specialized mechanisms of regulation and rRNA processing [51].

38.4% of *L. viridis* and 38.2% of *L. bilineata* were predicted to be covered by repeats. We observed double the amount of LTR-elements in *L. bilineata* compared to *L. viridis* (Table S4). LINE and SINE elements accessed in the tetrapod lineage cover almost identical amounts of the lacertid genomes.

Table S3. Number of predicted non-coding RNAs in lacertids. Non-coding RNA that were not classified as any other ncRNA type are putatively long non-coding RNAs (lincRNAs). The conserved lincRNAs were predicted based on the overlap of orthologous lacertid lincRNAs with conserved sites predicted from PhastCons.

**Species**	**miRNAs**	**snoRNAs**	**tRNAs**	**rRNAs**	**snRNAs (Spliceosomes)**	**Putative lincRNAs**	**Conserved lincRNAs**
Lacerta viridis	260	234	23060	136	403	9747	135
Lacerta bilineata	255	237	22662	155	411	8931	135

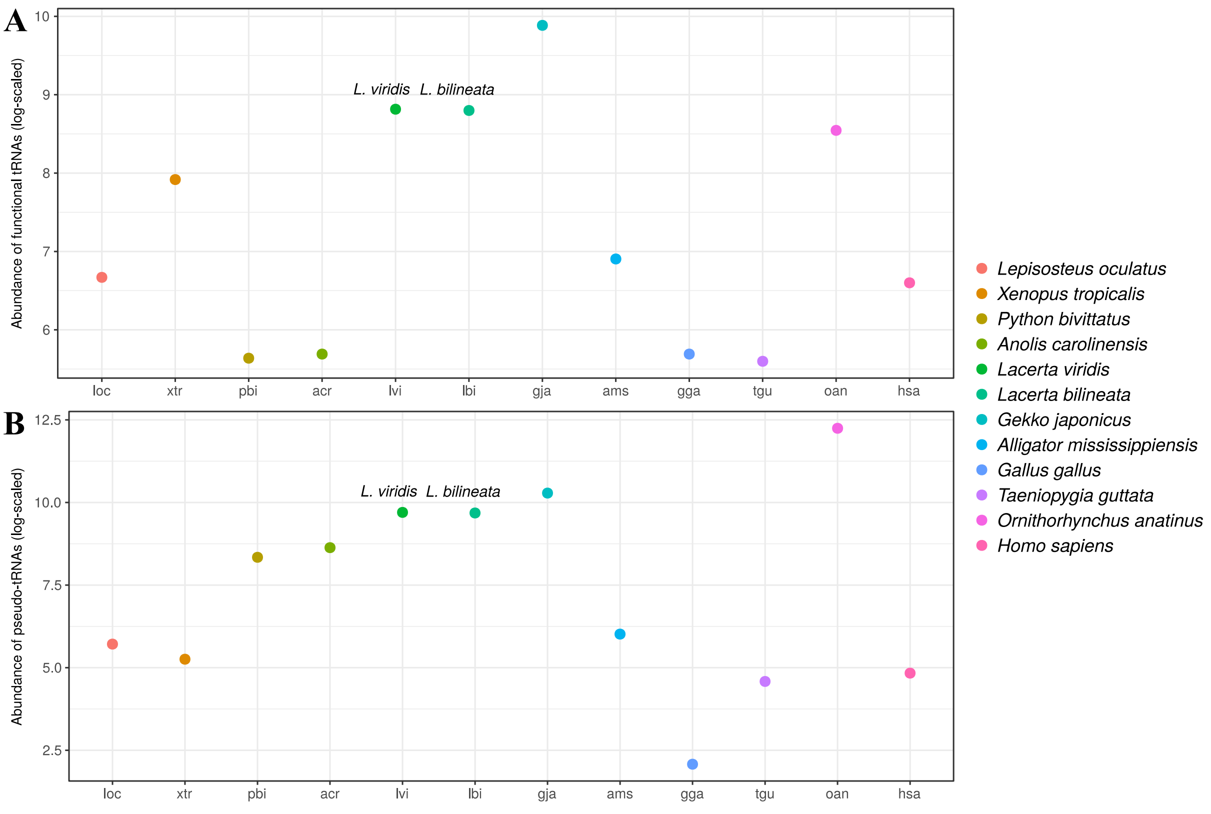

Figure S5. Abundance of tRNAs across selected sauropsids (ordered as Testudinates, Eusuchiates and Squamates). **A** Number of functional tRNAs across sauropsids. **B** Number of pseudo-tRNAs across sauropsids. The x-axis represents arbitrary labels for each species with their full names given in the figure legend in the same order and the y-axis represents $\log_{10}$-scaled counts of tRNAs. The lacertid and *G. japonicus* genomes encode the highest number of functional and pseudo tRNAs.

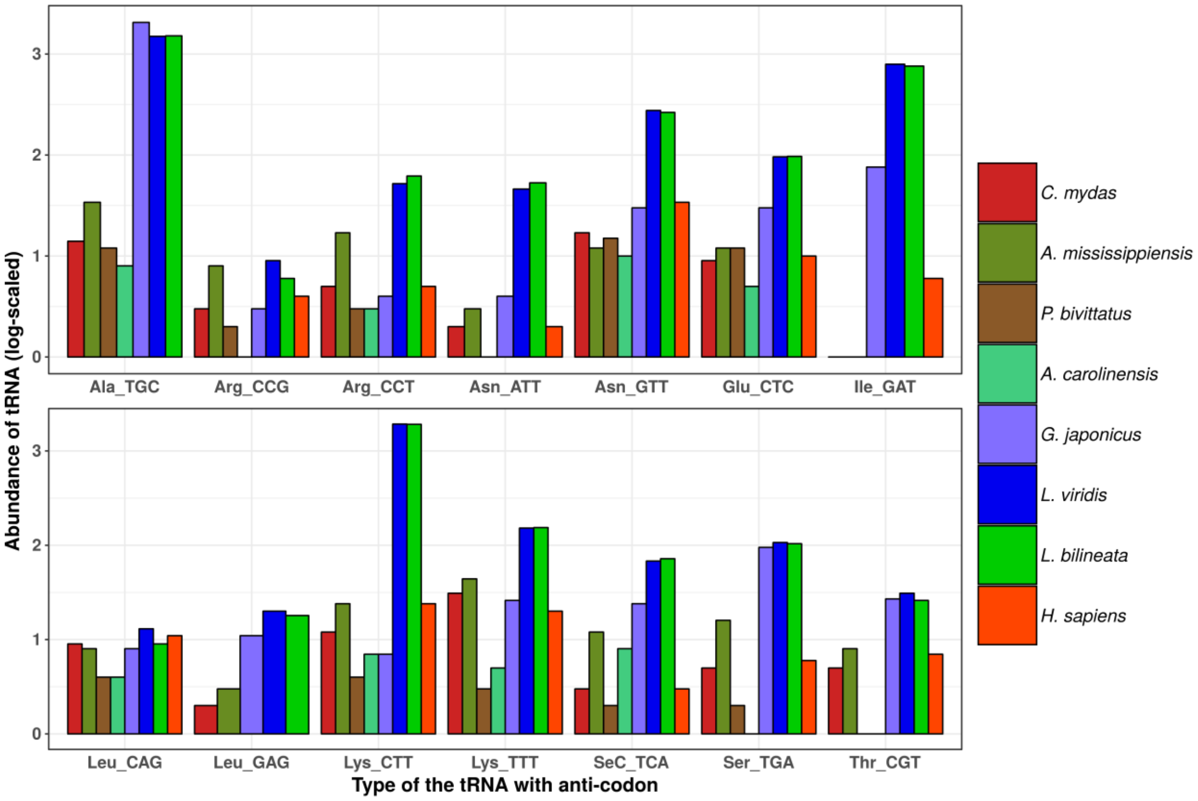

Figure S6. Counts of most abundant functional tRNAs in lacertids in comparison to other reptiles (y-axis is log_{10}-scaled). The anti-codon is given adjacent to the amino acid which will be loaded if the tRNA is functional (Arg – Arginine, Asn – Asparagine, Glu – Glutamate, Ile – Isoleucine, Lys – Lysine, SeC – Selenocysteine, Ser – Serine, Thr – Threonine, Sup - Suppressor). The most abundant functional tRNAs in lacertids (lvi and lbi) were compared to tRNA counts in *Chelonia mydas* (cmy), *Alligator mississippiensis* (ams), *Python bivittatus* (pbi), *Anolis carolinensis* (acr), *Gekko japonicus* (gja) and *Homo sapiens* (hsa).

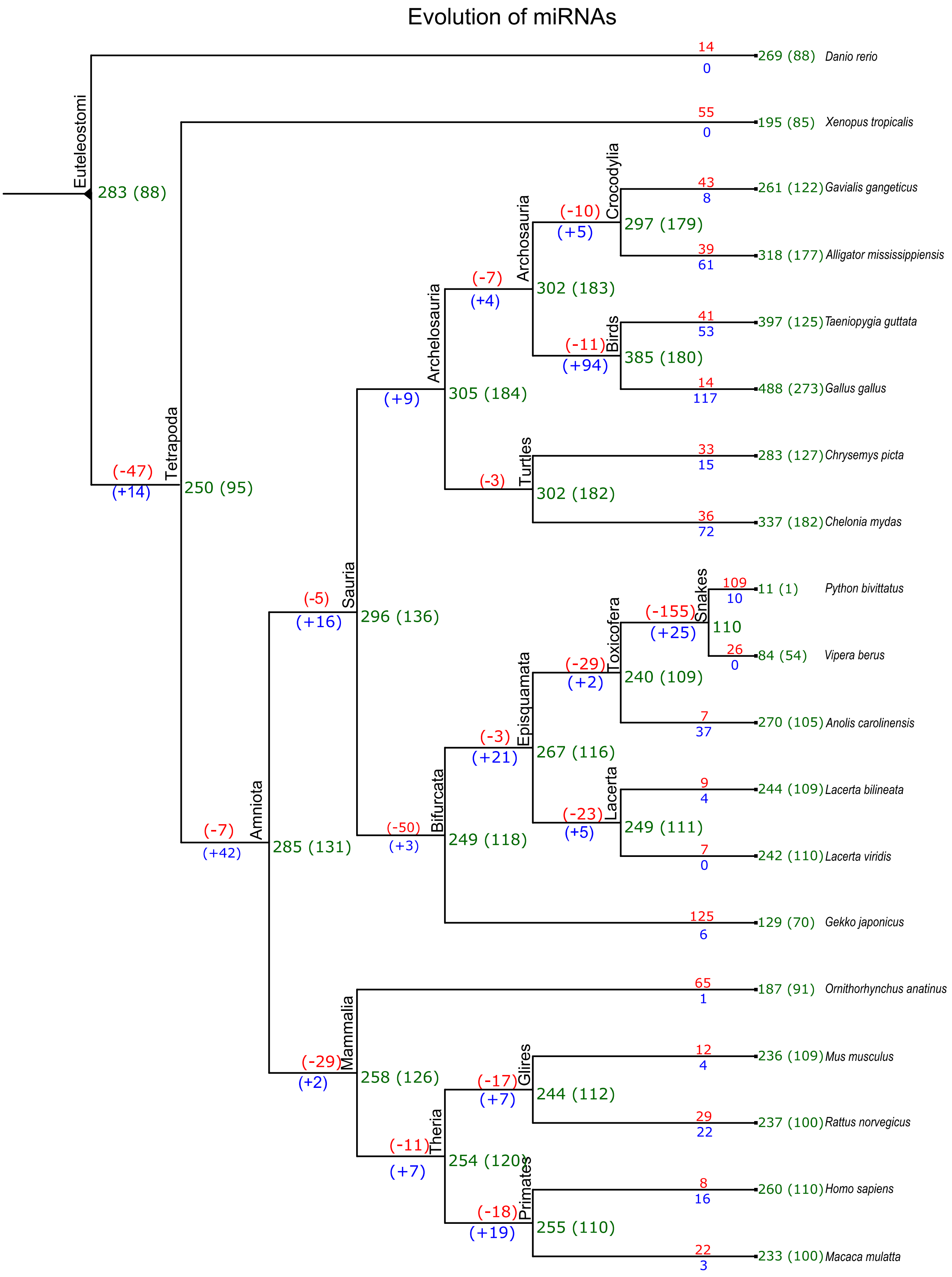

Figure S7. Duplications, gains and/or losses of miRNA families in different species ranging from fishes to mammals. The losses of individual miRNAs are indicated in red and the gains in blue over each branch. The numbers in green represent the number of individual miRNAs present, the number of total miRNA families are given in parenthesis. Some miRNAs were excluded as they did not align to any family.

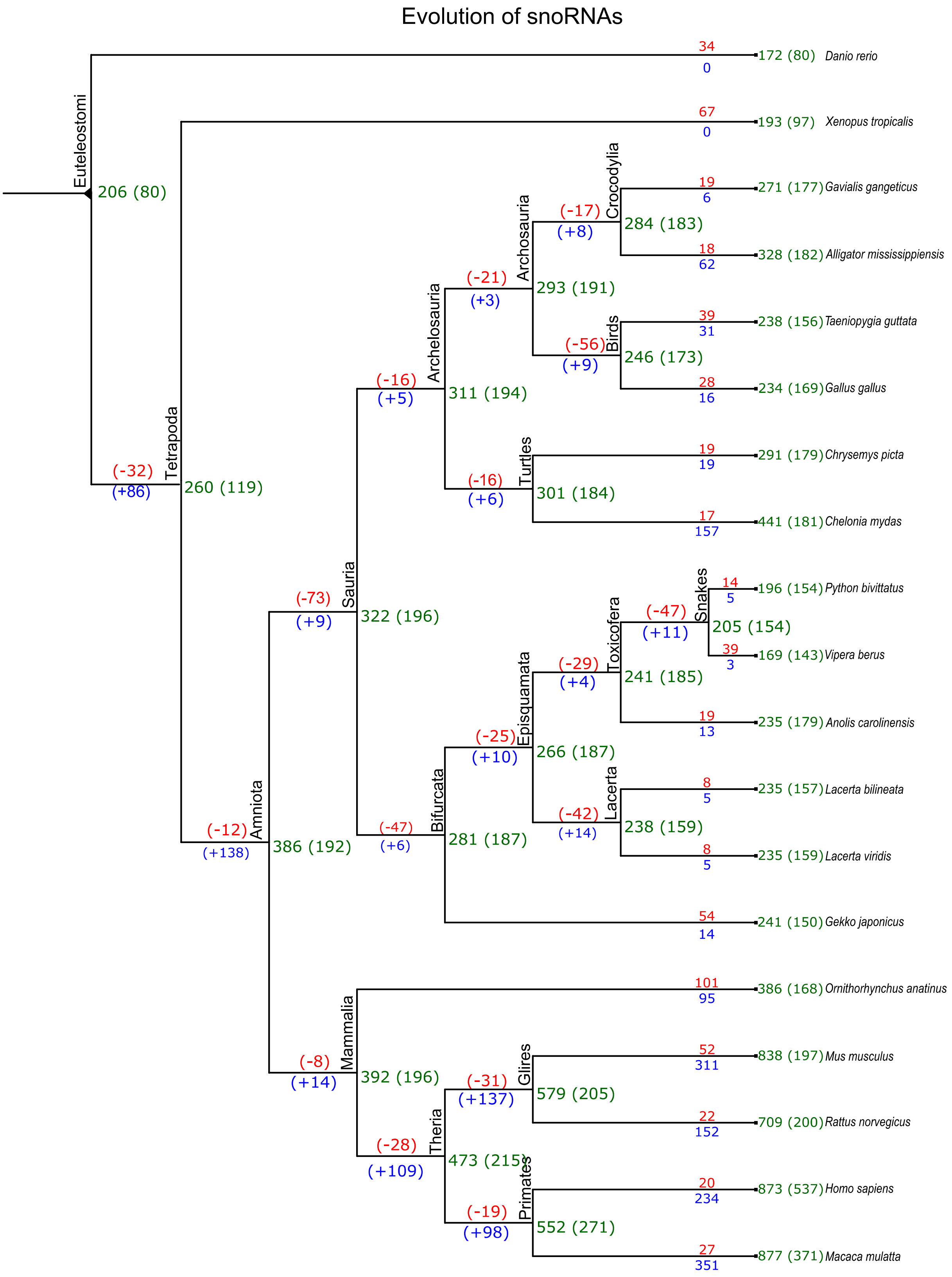

Figure S8. Duplications, gains and/or losses of snoRNAs. The losses of individual snoRNAs are indicated in red and the gains in blue over each branch. The numbers in green represent the number of individual snoRNAs, while the number of total snoRNA families is given in parenthesis.

Table S4. Percentage of LINEs, SINEs and DNA transposons in selected tetrapod species predicted with Repeatmasker using the repeats library of tetrapods. The predicted repeat elements are provided as percentage in the genome for each category and the genome versions are provided in braces. Note: For the genomes without the genome versions in UCSC, arbitrary genome versions have been used; the repeat element statistics for the lacertid species have been highlighted in bold.

Species (Genome version)	SINEs (% in genome)	LINEs (% in genome)	DNA transposons (% in genome)
Alligator mississippiensis (allMis1)	0.6	12.41	16.24
Anolis carolinensis (anoCar2)	4.19	13.14	9.5
Chelonia mydas (cheMyd1)	1.89	11.97	8.09
Gallus gallus (galGal4)	0.08	7.02	1.05
Gekko japonicus (gekJap1)	3.91	10.45	0.72
Homo sapiens (hg38)	8.78	14.76	1.29
***Lacerta bilineata* (lacBil1)**	**2.66**	**8.88**	**1.62**
***Lacerta viridis* (lacVir1)**	**2.65**	**8.54**	**1.64**
Lepisosteus oculatus (lepOcu1)	1.58	0.88	1.59
Mus musculus (mm9)	3.72	16.52	0.32
Ornithorhynchus anatinus (ornAna5)	20.05	19.95	0.88
Python bivittatus (pytBit5)	1.2	6.98	1.12
Taeniopygia guttata (taeGut1)	0.08	3.62	0.22
Xenopus tropicalis (xenTro3)	0.29	4.83	19.62

Table S5. Annotation of repeat-elements (percentage per genome) using Repeatmasker with the Tetrapod lineage and RepeatModeler with *de novo* libraries. The *de novo* repeat annotations from RepeatModeler did not include SINE elements.

**Type of Repeat-element**	***Lacerta virdis***		***Lacerta bilineata***	
	**Tetrapod lineage**	**RepeatModeler *de novo***	**Tetrapod lineage**	**RepeatModeler *de novo***
**SINEs**	2.66	-	2.65	-
**LINEs**	8.88	12.85	8.54	13.21
**LTR elements**	0.44	1.20	0.74	1.81
**DNA transposons**	1.62	0.23	1.64	0.14
**Unclassified**	0.05	24.09	0.05	23.08
**Total interspersed repeats**	13.64	38.37	13.61	38.23

SI-3. Characteristics of the Z-chromosome contigs

In *L. viridis*, Z-linked contigs totaled 13.8Mbp in length (24 contigs) and 205 protein-coding genes were assigned to the Z-chromosome. 14% of the Z-chromosome (1.95 Mbp) was covered with repeat elements, over half of which were LINE-elements (1.05 Mbp). As for non-coding RNA, 7 microRNAs, 1 snoRNA, 2 snRNAs (two U2 spliceosomal RNA), 46 functional tRNAs and 115 pseudo tRNAs were predicted on the Z-chromosome.

While in *L. bilineata,* 15.84 Mb of the genome (36 contigs) and 226 protein-coding genes were assigned to the Z-chromosome. 11 microRNAs, 1 snoRNA, 3 snRNAs (one each of U2, U4 and U6 spliceosomal RNAs), 53 functional tRNAs and 169 pseudo tRNAs were identified on the Z-chromosome which was 13.1% repetitive (2.08 Mbp). Similar to *L. viridis,* LINE-elements comprised of over half of the repeat elements (1.12 Mbp) on the Z-chromosome of *L. bilineata*.

SI-4. Gene flow between the two species

Table S6. Estimates of effective population size (N_e) at a different number of mutations of any type in either branch (K_{max}). Assuming mutation rates of 1.77 x 10^{-8} & 1 x 10^{-9} per site per generation under the parameters inferred from the best model, the population size of *L. bilineata* is smaller than *L. viridis*. Higher K_{max} leads to higher accuracy [52].

K_{max}	N_e for *L. Bilineata*		N_e for *L. Viridis*	
	μ = 1.77 x 10^{-8}	μ = 1 x 10^{-9}	μ = 1.77 x 10^{-8}	μ = 1 x 10^{-9}
1	3.231 x 10^4	5.719 x 10^5	6.436 x 10^4	11.39 x 10^5
2	3.573 x 10^4	6.324 x 10^5	8.001 x 10^4	14.16 x 10^5
3	3.789 x 10^4	6.706 x 10^5	9.540 x 10^4	16.89 x 10^5

Table S7. Parameter estimates for the best model (IM 2 B(x)–>V) which supports gene flow from *L. bilineata* to *L. viridis*. We calculated the effective population size (N_e), discrete divergence (T), within-lineage genetic diversity (θ), the ratio of effective population size between the common ancestor and the source population of the migrant (b) and the continuous migration rate in each generation (M). A generation time of 3.5 years and mutation rates of 1.77 x 10^{-8} & 1 x 10^{-9} per site per generation were assumed with a maximum of three mutations of any type in either branch (K_{max}=3).

Mutation rate (μ)	N_e for *L. bilineata*	N_e for *L. viridis*	Split time (Mya)	Ratio of effective population size (b)	Within-lineage genetic diversity (θ)	Discrete divergence (T)	Migration rate (M)
1.77 x 10^{-8}	3.789 x 10^4	9.540 x 10^4	1.151	2.518	0.537	4.34	0.288
1 x 10^{-9}	6.706 x 10^5	16.89 x 10^5	20.37	2.518	0.537	4.34	0.288

Table S8. Time of split (years before present) between *L. viridis* and *L. bilineata* under the best model (IM with migration from *L. bilineata* to *L. viridis*) assuming mutation rates of 1.77 x 10^{-8} & 1 x 10^{-9} with K_{max}=3. Since the mutation rates in lacertids are unknown, two different mutation rates were used to estimate the split time between *L. bilineata* to *L. viridis*.

Generation time	Split time (in Mya)	
	μ = 1.77 x 10^{-8}	μ = 1 x 10^{-9}
3	0.9863	17.46
3.5	1.151	20.37
4	1.315	23.28

SI-5. Accelerated sequence evolution

Through the analysis of conserved sites across tetrapods we predicted that 8% of the *L. viridis* genome (71 Mbp) was undergoing accelerated evolution. The coding regions affected by acceleration were enriched for the biological processes of RNA-dependent DNA biosynthetic process (GO:0006278; Fold-enrichment=32.79, *p*=1.59e-21, *FDR*=4.14e-18) and primarily overlapped with LINE-1 retro-transposable elements. The disparity in the number of LINEs was higher compared to other repeat elements between lacertid species (Table S5).

SI-6. Genome variation through SVs or rearrangements

Table S9. Characteristics of the SVs detected between *L. viridis* and *L. bilineata*. Since the genomes were converted to single-coverage for syntenic comparison, none of the duplications were detected from syntenies.

**Type**	**Deletion**	**Duplication**	**Insertion**	**Inversion**
**Number of Events**	18724	30	973	430
**Total bases (kbp)**	20937	705	757	16811
**Maximum size (bp)**	132486	79520	22792	842729
**Median size (bp)**	333	13385	267	10812
**Average size (bp)**	1118	23496	778	39095

Table S10. Number of genes affected by genomic rearrangements (or SVs) in coding and non-coding regions between *L. viridis* and *L. bilineata*. If no rearrangement overlapped with the respective feature, the value of the cell is marked by “-”. Note that the respective rearrangement category was counted at most once for each genomic feature even if it overlapped the same genomic feature multiple times; for the small ncRNAs only the features completely overlapping with rearrangements were considered. The overlaps of rearrangements with protein coding regions are divided into “entire gene” where rearrangement overlaps over complete gene and “partially in gene” where the overlap is localized covering a small region in the gene. UTRs – Untranslated regions.

**Type of genomic feature**			**Deletions**	**Insertions**	**Duplications**	**Inversions**
Protein coding regions	Partially in gene	Exons	42	13	8	78
		Introns	2843	167	10	98
		UTRs	-	-	-	-
	Entire gene		165	1	13	222
Non-coding regions	miRNA		-	-	-	5
	snoRNA		-	-	-	2
	snRNA		4	-	-	5
	tRNA (functional)		3	3	2	81
	tRNA (pseudo)		23	3	8	222
	lincRNA		1	-	-	1

The rearrangements overlapping coding regions cover the entire exonic feature except EXD2 (62 bp deletion) and HERC2 (70 bp deletion) which were interrupted by deletions inside the reading frame and a gene encoding an uncharacterized protein (homologous to R4G973 protein in *A. carolinensis*) with an in-frame insertion. The gene with an in-frame insertion encodes an uncharacterized protein (homologous to R4G973 protein in *A. carolinensis*) consisting of a reverse transcriptase domain (PF0078), but the insertion occurs outside this domain. EXD2 promotes efficient homologous recombination (HR) and provides resistance to double-stranded breaks (DSBs) through its exonuclease activity. Furthermore, cells depleted for EXD2 have shown spontaneous chromosomal instability [53]. However, the deletion in EXD2 occurs outside the 3’-5’ exonuclease domain. On the contrary, the deletion in HERC2 gene occurs in one of the five RCC1 repeat functional domains (PF00415 - Regular of chromosome condensation (RCC1) repeat). HERC2 is known to promote RNF8–Ubc13 interaction required for DNA repair and it has been speculated that mutations in HERC2 compromise genomic stability [54].

The genes with in-frame aberrations are known to be non-essential. EXD2 in humans are known to be complemented by the MRE11 gene which is active in the same pathways as EXD2 [53]. HERC2 is not mandatory for RNF8–Ubc13 interaction and DNA damage repair occurs even in the absence of HERC2, primarily through ubiquitin ligase activity of RNF8 [54].

SI-7. Structural selection in non-coding RNA

Table S11. Structural characterization of ncRNAs. From the initial set, only conserved groups (d≤10.0) were screened for selection. Sequences with high selection scores (s≥10.0 for small ncRNAs) were visually inspected and only the stable structures and those substantially similar to group consensus were suggested as candidates for positive selection. A lower selection score (s≥4.0) was used for the lincRNAs and those candidates that diverged between *L. viridis* and *L. bilineata* have been reported.

**Type of ncRNA**	**Initial number of groups**	**Number of Conserved groups**	**Sequences with high selection scores**	**Number of candidates**
miRNAs	80	39	8	2
snoRNAs	143	71	3	1
tRNAs	127	26	3	0
lincRNAs	135	82	2	2

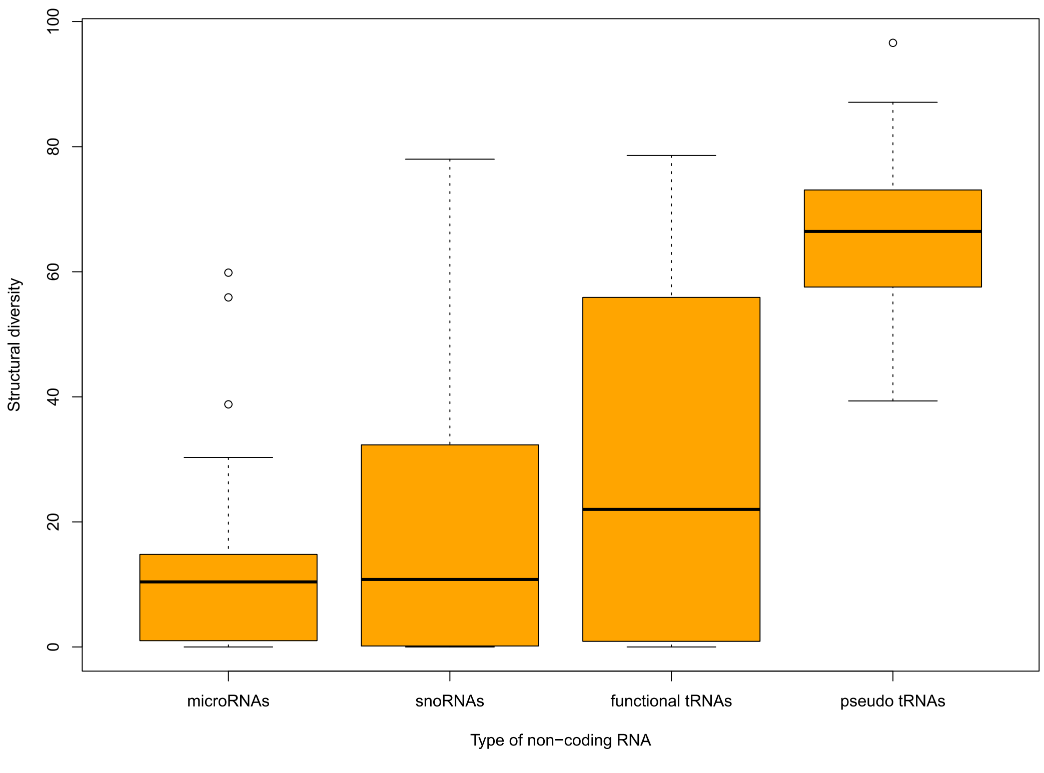

Figure S9. Structural diversity of non-coding RNA orthologous. The structural diversity in each ncRNA class is based on the median base pair distance (d-score) of each family in the ncRNA class and its consensus. The most conserved class are microRNAs, followed by snoRNAs and functional tRNAs, pseudo tRNAs are the most diverse.

We detected five ncRNA candidates whose structures evolved differently within lacertids (two microRNAs, one snoRNA and local structures of two lincRNAs). For small ncRNAs, the structures differ in one of the lacertid species compared to the consensus indicating changes in function (Figure S8a-c). While for the lincRNAs, the structures are similar, with one lincRNA containing only one inner stem in the upper part of the *L. viridis* structure and two inner stems

in the *L. bilineata* structure (Figure S8e) and the other lincRNA containing an observable distinction in the length of the very stable upper stem (Figure 8d) stem indicating functional divergence between the two lacertids.

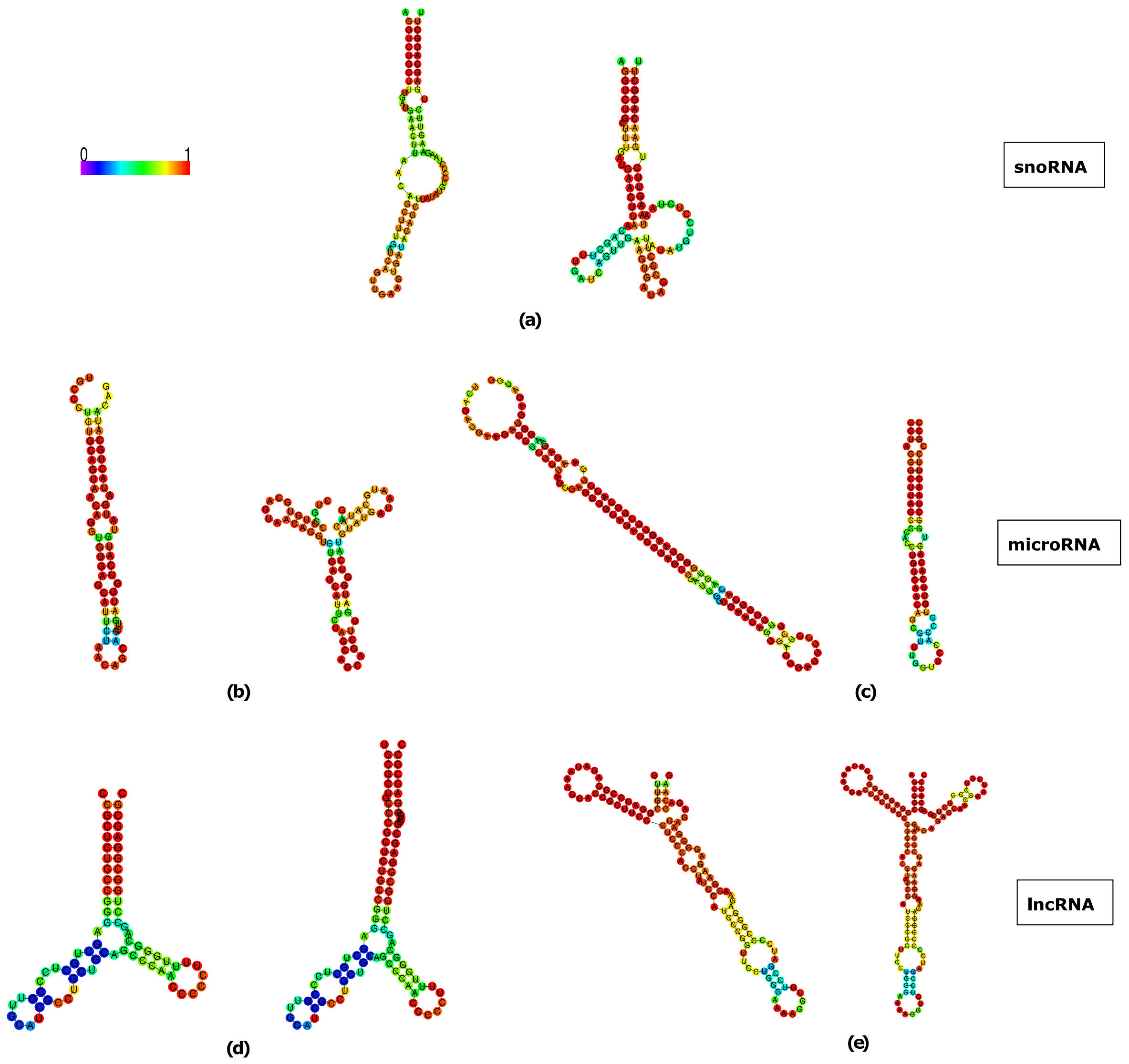

Figure S10. Secondary structures of snoRNA (a) and miRNAs (b, c) undergoing positive selection in lacertids (positively selected structure on the right, consensus on the left) and lincRNAs (d,e) that diverged between *L. viridis* (left) and *L. bilineata* (right). (a) SNORD61 was positively selected in *L. bilineata*; (b and c) secondary structures of mir-6516 and mir-27a respectively, which are both positively selected in *L. viridis*; (d and e) local structures of LiNC66 and LiNC29, respectively. The structures were predicted with RNAfold and the colours of the nucleotides represent their frequency in the structural ensemble (colour bar). Red bases occur in ≥ 90%; green to yellow occur in ≥ 50% and light blue to purple in less than 50% of the structures.

SI-8. Conservation of UV-vision and skin pigmentation and adaptive evolution to UV-B response

All the key residues that tune the absorption spectrum to UV-light in the SWS1 opsin are highly conserved [47, 48]. This was observed with snakes and lizards as the foreground and Aves as the background branch (92% of conserved sites at dN/dS ratio of 0.028). The SWS1 proteins in the lacertids evolved neutrally.

The genes encoding pigmentation proteins (MC1R) and UV-reflecting pigments (ECE2) were neutrally evolving across lizards including *Pogona vitticeps, Thamnophis sirtalis, Anolis carolinensis* and *Gekko japonicus*. Serine/threonine kinase 11 (STK11) and a paralog of hyaluronidases (HYAL2), both known to be triggered in the skin cells on exposure to UV-B (GO:0071493), were positively selected in the lacertid branch compared to other lizards (2.5% sites and 2.2% sites respectively). Furthermore, a positively selected site was observed in HYAL1 (without *G. japonicus* since this region was fragmented in their genome) in the domain containing a signal peptide in *L. viridis*. Another positively selected site which lies in the glycoside hydrolase family 56 domain (PF01630.13) of HYAL1 encodes different amino-acids within *L. bilineata* and *L. viridis*.

SI-9. Possible biological implications of protein-coding genes under selection

78.55% of the bases of the *L. viridis* genome aligned to the other genomes in the multiple-species genome alignment (single-coverage). 5.63% of the bases of the *L. viridis* genome were most-conserved (from multiple genome alignments).

Table S12. Lineage-specific selection events in genes observed in the lacertid ancestor or one of the two lacertid species. The birth events (selection occuring in a selected branch after divergence from ancestor) in genes are represented by “Gain” and death events (loss of selection) by “Loss”.

Type of event	Lineage of the observed event	Gene name	Ontologies
Gain	Lacertid ancestor	WFS1; Wolframin ER Transmembrane Glycoprotein	Signal transduction, cyclin-CDK, visual and sound perception
		NRIP1; Nuclear Receptor Interacting Protein 1	Ovarian follicle rupture
Loss	Lacertid ancestor	SNCA; Synuclein Alpha protein	Adult locomotory behavior and synaptic transmission
		NR2F2; Nuclear Receptor Subfamily 2 Group F Member 2	Placental development

		IGF1R; Insulin Like Growth Factor 1 Receptor	Immune response
Gain	*L. viridis*	ARHGAP35; Rho GTPase Activating Protein 35	Neural development
		FOXJ1; Forkhead Box J1 protein	Brain development
		BBS12; Bardet-Biedl Syndrome 12 protein	Eating behavior
Gain	*L. bilineata*	EIF4G1; Eukaryotic Translation Initiation Factor 4 Gamma 1	Behavioral fear response
		EPN1; Essential nuclear protein 1	Neuron differentiation and embryonic organ development
		ITGB8; Integrin beta-8	Cartilage development

A few non-coding elements were conserved only in either *L. viridis* (SNORA75 and snoU109) or *L. bilineata* (U6, U4 and U2 snRNAs; mir-22) indicating different selective pressures in the regulatory machinery of the genome. Repeat elements also evolved differently in the lacertids, which was furthermore confirmed by their presence in genomic rearrangements. The protein coding regions affected by lineage-specific negative selection (detected through dless) and overlapping with mobile elements were detected in *L. bilineata* (LINE-elements: L1, L2, RTE and satellite repeat: MMSAT4).

Table S13. Positively selected genes identified with the branch-site models of PAML. Selection was tested with either of *L. viridis, L. bilineata* or lacertids in the foreground branch

**Foreground branch**	**Species' in the Background**	**#positively selected genes**
Lacertids (*L. viridis, L. bilineata)*	*Anolis carolinensis, Gallus gallus, Homo sapiens, Xenopus tropicalis, Lepisosteus oculatus*	3
Lacertids (*L. viridis, L. bilineata)*	*Anolis carolinensis, Pogona vitticeps, Gekko japonicus*	33
L. viridis	*L. bilineata, Anolis carolinensis, Pogona vitticeps, Gekko japonicus*	17
L. bilineata	*L. virdis, Anolis carolinensis, Pogona vitticeps, Gekko japonicus*	14

A few genes under positive selection between lacertids were involved with transcription factor activity and mRNA splicing (Table S14). The positively selected genes in the lacertid foreground branch are provided in Table S15.

Table S14. Genes with positively selected sites identified through the branch-site models of PAML with either *L. viridis* or *L. bilineata* in the foreground branch and the uniprot ID along with the gene-ontologies (incomplete list) of the most similar protein. The positively selected sites (PSS) are provided along with the amino-acid (AA) change (position and transition), the pFAM domain they occur in or PANTHER family of the protein. Note – If the PSS are not present in any predicted domain, the last column is marked with '-'.

Foreground branch	Gene name	Uniprot ID	Gene ontology	AA change	Location of PSS
L. viridis	ASB3	A0A093H9J2	intracellular signal transduction	56 S(A)	PTHR24188 - Unnamed
	S22A2	G1KC28	dopamine transport	126 A(V)	PF00083 - Sugar (and other) transporter
	ZN777	H9G9J0	nucleic-acid binding	675 A(I)	-
	ENOF1	G1KHZ4	L-fuconate dehydratase activity; magnesium ion binding	116 Y(N)	-
	THG1	H9G7D3	tRNA processing ATP binding; tRNA guanylyltransferase activity	361 G(C)	PTHR12729:SF6 – tRNA-His guanylyl transferase-related
	MERTK1	H9GAY1	positive regulation of phagocytosis; retina development in eye; spermatogenesis; vagina development	87 G(S)	PTHR24416:SF257 - Tyrosine-protein kinase mer
	RPA2	F7B818	embryo implantation; transcription from RNA polymerase I promoter; DNA binding	578 T(M)	PF04565 - RNA polymerase Rpb2, domain 3
	TDRD3	G1KDC8	chromatin binding; methylated histone binding; transcription co-activator activity	405 Y(H)	-
	K1107	M7C7Q5	catalytic activity	1261 T(M)	PTHR22427:SF2 - SI:CH73-389B16.2
	ARL4D	G1KNI4	small GTPase mediated signal transduction	132 G(S)	-
	ATG3	G1KJY0	protein ubiquitination; Atg12 transferase activity	178 E(M)	PF03986 - Autophagocytosis associated protein (ATG3), N-terminal domain
	LR16A	G1K9V1	protein complex binding; urate metabolic process	281 A(V)	-
	LRCC1	G1KPG3	Uncharacterized	281 A(V)	-

	PGLT1	G1KP76	cardiovascular system development; positive regulation of Notch signaling pathway; regulation of gastrulation	135 P(V)	-
	UGPA	L9KQ61	UDP-glucose metabolic process	20 I(S)	-
	HYAL1	Q12794	response to UV-B	22 F(S)	PTHR11769:SF23 - Hyaluronidase-1
	ARSB	P15848	chondroitin sulfate catabolic process	100 D(Y)	PTHR10342:SF255 - Arylsulfatase B
L. bilineata	CT2NL	G1KUI5	negative regulation of transmembrane transport; protein dephosphorylation	307 A(S)	PTHR23166:SF9 - CTTNBP2 N-terminal-like protein
	CLP1L	U3JHI8	apoptotic process	405 T(H)	PF05602 - Cleft lip and palate transmembrane protein 1 (CLPTM1)
	STAR7	H9GMT5	lipid binding	382 E(Q)	PTHR19308 – Unnamed
	CASC3	G1KB37	mRNA processing; RNA binding; ubiquitin protein ligase binding	368 T(K)	-
	F169A	G1KIB1	uncharacterized	579 T(Q)	-
	UBIP1	G1KU12	regulation of transcription from RNA polymerase II promoter; transcription factor activity	389 A(T)	PTHR11037 - Unnamed
	FGL1	G1KFL2	uncharacterized	25 A(L)	PF00147 - Fibrinogen beta and gamma chains, C-terminal globular domain
	NAA30	H9G450	peptide alpha-N-acetyltransferase activity	7 E(S)	PTHR23091 – Unnamed
	MGST3	J3S931	transferase activity	Multiple	PTHR10250:SF17 - Microsomal glutathione S-transferase
	ILEU	R4GC66	serine-type endopeptidase inhibitor activity	343 M(V)	PF00079 - Serpin (serine protease inhibitor)
	SRPR	H9G398	GTPase activity; RNA binding; signal recognition particle binding	323 K(V)	-
	RAE1	H9GKI1	protein geranyl-geranylation; small GTPase mediated signal transduction	57 S(H), 210 S(P)	PF00996 - GDP dissociation inhibitor

	PSPC1	G1KAB1	activation of innate immune response; mRNA splicing; regulation of circadian rhythm	268 R(V)	PTHR23189:SF14 - Paraspeckle component 1
	GP155	H9GA78	cognition; intracellular signal transduction; transmembrane transport	634 N(D)	PTHR22829:SF5 - Integral membrane protein GPR155

Table S15. Positively selected genes detected through branch-site model of PAML in the lacertid branch (ancestor of *L. viridis* and *L. bilineata*). The Uniprot ID of the protein orthologs, gene ontologies (incomplete list) and PANTHER family information are provided. Note - The names of the positively selected genes in the lacertid branch with distant species (non lizards) in the background branches are highlighted in bold.

Gene names	Uniprot ID of orthologs	Gene ontology (biological processes or molecular functions)	PANTHER family
**NASP**	K7FW38	histone binding; HSP90 protein binding	PTHR15081:SF1 - Nuclear antigenic sperm protein
**PDLI1**	U3I6K3	regulation of transcription, DNA-templated	PTHR24214:SF5 - PDZ and LIM Domain 1
**RTKN**	D2HH63	septin cytoskeleton organization; Rho protein signal transduction	PTHR21538 - Rhotekin
ABHD3	G1KH59	phosphatidylcholine 1-acylhydrolase activity; phospholipase A2 activity	PTHR10794:SF50 - Phospholipase ABHD3
CB047	G1KS02	inner mitochondrial membrane organization; ribosome binding	PTHR13333 – Unnamed
CBLB	G1KFF7	epidermal growth factor receptor signaling pathway; receptor tyrosine kinase binding; SH3 domain binding	PTHR23007:SF3 - E3 ubiquitin-protein ligase CBL-B
CEP57	V8NUN1	microtubule binding	PTHR19336:SF11 - Centrosomal protein of 57 kDa
CO1A2	K7G8R1	blood vessel development; skeletal system development; skin morphogenesis; SMAD binding	PTHR24023:SF568 - Collagen alpha-2 I chain
CRBL2	H9G8V7	transcription factor activity, sequence-specific DNA binding	PTHR21051:SF4 - CAMP-responsive element-binding protein-like 2
CSCL1	G1KEV2	nucleic acid binding	PTHR13018:SF24 - CSC1-like protein 1
DKK1	G5CAC0	embryonic limb morphogenesis; face morphogenesis; forebrain development; motor learning; Wnt signaling pathway involved in somitogenesis	PTHR12113:SF11 - Dickkopf-related protein 1

DMBT1	V8NZ52	scavenger receptor activity	PTHR19331 – Unnamed
FANK1	G1K8U3	positive regulation of sequence-specific DNA binding transcription factor activity	PTHR24183 - Fibronectin type 3 and ankyrin repeat domains protein 1
GRPE1	G1KA62	adenyl-nucleotide exchange factor activity; chaperone binding; binding	PTHR21237:SF25 - GRPE protein homolog 1, mitochondrial
HEM0	G1KKY7	erythrocyte differentiation; hemoglobin biosynthetic process; response to hypoxia	PTHR13693:SF58 - 5-Aminolevulinate synthase, erythroid-specific, mitochondrial
HYAL2	Q12891	cellular response to UV-B	PTHR11769:SF6 - Hyaluronidase-2
KCMA1	A0A093QP71	large conductance calcium-activated potassium channel activity	PTHR10027 - Unnamed
KSYK	G1K8U5	blood vessel morphogenesis; innate immune response; SH2 domain binding; Toll-like receptor binding	PTHR24418:SF236 - Tyrosine-protein kinase
KY	G1KJ17	Uncharacterized	PTHR11039 - Nebulin
LGMN	J3S4K6	peptidase activity	PTHR12000:SF3 - Legumain
LOXL3	H9GEG1	lung development; palate development; spinal cord development; scavenger receptor activity	PTHR19331 - Unnamed
MRP2	A0A091LMA3	ATPase activity, coupled to transmembrane movement of substances; ATP binding	PTHR24223 - Unnamed
MTNA	G1KBX7	S-methyl-5-thioribose-1-phosphate isomerase activity	PTHR10233 – Unnamed
NBAS	G1KKP9	nuclear-transcribed mRNA catabolic process; Golgi to ER; SNARE binding	PTHR15922:SF2 - Neuroblastoma-amplified sequence
NRAM2	G1KMM6	dendrite morphogenesis; erythrocyte development; learning or memory	PTHR11706:SF40 - Natural resistance-associated macrophage protein 2
OSBL9	G1KGY9	lipid transport	PTHR10972:SF27 - Oxysterol-binding protein-related protein 9
PDIA4	G1KPJ9	protein disulfide isomerase activity; RNA binding	PTHR18929:SF110 - Protein disulfide-isomerase
PDLI7	J3S4U2	metal ion binding	PTHR24214:SF0 - PDZ and LIM domain protein 7
RNF11	F6S2Z9	ubiquitin protein ligase activity	PTHR14155 – Unnamed

SCPDL	V8NWT5	oxidoreductase activity	PTHR12286:SF5 - Saccharopine dehydrogenase-like oxidoreductase
SODC	B5G2U2	locomotory behavior; ovarian follicle development; sensory perception of sound; spermatogenesis	PTHR10003 – Unnamed
THRB	G1KCA5	heparin binding; lserine-type endopeptidase activity	PTHR24254:SF10 - Prothrombin
TM183	J3SFG7	Uncharacterized	PTHR20988:SF2 - Transmembrane protein 183A-related
TOM34	V8P625	uncharacterized	PTHR22904 – Unnamed
TRM1L	H9G676	tRNA (guanine-N2-)-methyltransferase activity; tRNA binding	PTHR10631:SF1 - TRMT1-like protein
VIP2	H9GCE8	inositol metabolic process; acid phosphatase activity	PTHR12750:SF10 - Inositol hexakisphosphate and diphosphoinositol-pentakis-phosphate kinase 2

SI-10. Evolution of transcription factors

Six KZNF genes have amino-acid differences in the binding specific-sites of their zinc-finger domains (Table S16). The isoforms of these six KZNF genes were detected in the ovarian tissue alone in *L. viridis*; but in *L. bilineata* we found alternative isoforms for two genes (Z658B - brain and ovary; ZN281 - brain and kidney).

Table S16. Zinc-finger proteins with amino acid differences between both lacertids at DNA-contacting positions. The names of the ZNFs are given with their identity, number of ZNF domains and the number of domains with amino-acid differences within the finger-nail region (AA position 6-12) which determines binding specificity. The tissue name represents the source of the longest transcript.

L. viridis			***L. bilineata***			**Protein identity (in %)**	**# domains with differences in DNA-binding amino acids**
**Gene name**	**Tissue**	**# ZNF domains**	**Gene Name**	**Tissue**	**# ZNF domains**		
Z658B	Ovary	11	Z658B	Ovary	9	98	1
KLF16	Ovary	6	KLF16	Ovary	5	96	1
ZN574	Ovary	32	ZN526	Ovary	24	99	1
ZNF572	Ovary	2	ZNF208	Ovary	2	97	1
ZNF420	Ovary	1	ZNF41	Heart	2	95	2
ZN281	Ovary	3	ZN281	Brain	10	85	1

SI-11. Evolutionary differences through rearrangements

The ZNF420 gene (coding for a transcription factor that negatively-regulates p53-mediated apoptosis [55]) is entirely covered by an inversion and harbors DNA-binding domain differences between the lacertids. Deletions affected only the introns of genes with PSS while inversions were found to occur in the exonic regions of genes with PSS. Nine genes with PSS (LRCC1, S22A2, MERTK, RPA2, K1107, MGST3, TDRD3, ATG3, NAA30) were affected by intronic deletions and three genes (UGPA, TDRD3, GPR155) were affected by inversions covering multiple exons (and their adjacent introns). The GPR155 gene (containing 17 transmembrane helices) and contains PSS in *L. bilineata* while entirely covered by a large inversion (843 kbp inversion) compared to *L. viridis*. The inversions in TDRD3 (41.5 kbp) covered two exons with the three adjacent introns, and the inversion in UGPA (42 kbp) covered three exons along with their four adjacent introns. Sites within both genes were positively selected in *L. viridis*. The integral membrane protein GPR155 with positively selected of sites in *L. bilineata* is highly expressed in the fore-brain of humans[56] and is involved in cognitive functions[57]. TDRD3 is directly associated with oocyte formation[58] and interacts with FMRP linked to developmental problems[59] while UGPA is an active contributor for the storage of glycogen in muscle tissues[60]. The inversion in TDRD3 was confirmed through the alignment of the transcript orthologs from the other species.

Table S17. Boschloo's exact test (two-sided) for each category of rearrangement for association with positive selection. Since insertions and duplications did not overlap with PSGs, they were excluded. Note: Total minus R represents the number of rearrangements of other categories i.e. by subtracting the number of rearrangements of the specific category (R) being tested from the total number of rearrangements. One gene is covered by both an exonic inversion and intronic deletion. PSGs – Positively selected genes; NPSGs – Non positively selected genes.

**Type**	**Category of Rearrangement (R)**		**Number of genes affected by rearrangements (Total)**	**Number of genes without overlapping rearrangements (NR)**	**Number of genes in collinear blocks (CB)**
	**Deletion**	**Inversion**			
**PSGs**	9	3	11	20	5
**NPSGs**	869	69	955	3206	1111
**Difference in proportion (R, Total minus R)**	-0.09	0.20			
***p*-value (R, Total minus R)**	0.89	0.028			
**Difference in proportion (R, NR)**	0.09	0.11			
***p*-value (R, NR)**	0.12	0.009			
**Difference in proportion (R,CB)**	0.204	0.32			
***p*-value (R, CB)**	0.08	0.006			

References

1. Renaud G, Stenzel U, Kelso J: **leeHom: adaptor trimming and merging for Illumina sequencing reads**. *Nucleic Acids Res* 2014, **42**(18):e141.
2. Magoc T, Salzberg SL: **FLASH: fast length adjustment of short reads to improve genome assemblies**. *Bioinformatics* 2011, **27**(21):2957-2963.
3. Dai M, Thompson RC, Maher C, Contreras-Galindo R, Kaplan MH, Markovitz DM, Omenn G, Meng F: **NGSQC: cross-platform quality analysis pipeline for deep sequencing data**. *BMC Genomics* 2010, **11**.
4. Chaisson MJ, Tesler G: **Mapping single molecule sequencing reads using basic local alignment with successive refinement (BLASR): application and theory**. *BMC Bioinformatics* 2012, **13**:238.
5. Bushnell B: **BBMap**. In.
6. Hackl T, Hedrich R, Schultz J, Forster F: **proovread: large-scale high-accuracy PacBio correction through iterative short read consensus**. *Bioinformatics* 2014, **30**(21):3004-3011.
7. Gregory TR: **Animal Genome Size Database**. 2017.
8. Simpson JT, Wong K, Jackman SD, Schein JE, Jones SJ, Birol I: **ABySS: a parallel assembler for short read sequence data**. *Genome Res* 2009, **19**(6):1117-1123.
9. Ye C, Hill CM, Wu S, Ruan J, Ma ZS: **DBG2OLC: Efficient Assembly of Large Genomes Using Long Erroneous Reads of the Third Generation Sequencing Technologies**. *Sci Rep* 2016, **6**:31900.
10. Ye C, Ma Z: **Sparc: a sparsity-based consensus algorithm for long erroneous sequencing reads**. *PeerJ* 2016, **4**:e2016.
11. Walker BJ, Abeel T, Shea T, Priest M, Abouelliel A, Sakthikumar S, Cuomo CA, Zeng Q, Wortman J, Young SK *et al*: **Pilon: an integrated tool for comprehensive microbial variant detection and genome assembly improvement**. *PLoS One* 2014, **9**(11):e112963.
12. Nishimura O, Hara Y, Kuraku S: **gVolante for standardizing completeness assessment of genome and transcriptome assemblies**. *Bioinformatics* 2017:btx445.
13. Hara Y, Tatsumi K, Yoshida M, Kajikawa E, Kiyonari H, Kuraku S: **Optimizing and benchmarking de novo transcriptome sequencing: from library preparation to assembly evaluation**. *BMC Genomics* 2015, **16**(1):977.
14. Rice P, Longden I, Bleasby A: **EMBOSS: The European Molecular Biology Open Software Suite**. *Trends Genet* 2000, **16**.
15. Langmead B, Salzberg SL: **Fast gapped-read alignment with Bowtie 2**. *Nat Methods* 2012, **9**(4):357-359.
16. Hoff KJ, Lange S, Lomsadze A, Borodovsky M, Stanke M: **BRAKER1: Unsupervised RNA-Seq-Based Genome Annotation with GeneMark-ET and AUGUSTUS**. *Bioinformatics* 2016, **32**(5):767-769.
17. Jones P, Binns D, Chang HY, Fraser M, Li W, McAnulla C, McWilliam H, Maslen J, Mitchell A, Nuka G *et al*: **InterProScan 5: genome-scale protein function classification**. *Bioinformatics* 2014, **30**(9):1236-1240.
18. Finn RD, Coggill P, Eberhardt RY, Eddy SR, Mistry J, Mitchell AL, Potter SC, Punta M, Qureshi M, Sangrador-Vegas A *et al*: **The Pfam protein families database: towards a more sustainable future**. *Nucleic Acids Res* 2016, **44**(D1):D279-285.
19. Finn RD, Attwood TK, Babbitt PC, Bateman A, Bork P, Bridge AJ, Chang H-Y, Dosztányi Z, El-Gebali S, Fraser M *et al*: **InterPro in 2017—beyond protein family and domain annotations**. *Nucleic Acids Research* 2017, **45**(Database issue):D190-D199.
20. Thomas PD, Campbell MJ, Kejariwal A, Mi H, Karlak B, Daverman R, Diemer K, Muruganujan A, Narechania A: **PANTHER: a library of protein families and subfamilies indexed by function**. *Genome Res* 2003, **13**(9):2129-2141.
21. Mi H, Dong Q, Muruganujan A, Gaudet P, Lewis S, Thomas PD: **PANTHER version 7: improved phylogenetic trees, orthologs and collaboration with the Gene Ontology Consortium**. *Nucleic Acids Res* 2010, **38**(Database issue):D204-210.

22. Grabherr MG, Haas BJ, Yassour M, Levin JZ, Thompson DA, Amit I, Adiconis X, Fan L, Raychowdhury R, Zeng Q *et al*: **Full-length transcriptome assembly from RNA-Seq data without a reference genome**. *Nat Biotechnol* 2011, **29**(7):644-652.
23. Haas BJ, Papanicolaou A, Yassour M, Grabherr M, Blood PD, Bowden J, Couger MB, Eccles D, Li B, Lieber M *et al*: **De novo transcript sequence reconstruction from RNA-seq using the Trinity platform for reference generation and analysis**. *Nat Protoc* 2013, **8**(8):1494-1512.
24. Altschul SF, Gish W, Miller W, Myers EW, Lipman DJ: **Basic local alignment search tool**. *J Mol Biol* 1990, **215**(3):403-410.
25. Camacho C, Coulouris G, Avagyan V, Ma N, Papadopoulos J, Bealer K, Madden TL: **BLAST+: architecture and applications**. *BMC Bioinformatics* 2009, **10**:421.
26. Uniprot C: **UniProt: the universal protein knowledgebase**. *Nucleic Acids Research* 2017, **45**(D1):D158-D169.
27. Eddy SR: **Accelerated Profile HMM Searches**. *PLoS Comput Biol* 2011, **7**(10):e1002195.
28. Consortium GO: **Gene Ontology Consortium: going forward**. *Nucleic Acids Res* 2015, **43**(Database issue):D1049-1056.
29. Petersen TN, Brunak S, von Heijne G, Nielsen H: **SignalP 4.0: discriminating signal peptides from transmembrane regions**. *Nat Methods* 2011, **8**(10):785-786.
30. Krogh A, Larsson B, von Heijne G, Sonnhammer EL: **Predicting transmembrane protein topology with a hidden Markov model: application to complete genomes**. *J Mol Biol* 2001, **305**(3):567-580.
31. Wu TD, Watanabe CK: **GMAP: a genomic mapping and alignment program for mRNA and EST sequences**. *Bioinformatics* 2005, **21**(9):1859-1875.
32. Lechner M, Findeiss S, Steiner L, Marz M, Stadler PF, Prohaska SJ: **Proteinortho: detection of (co-)orthologs in large-scale analysis**. *BMC Bioinformatics* 2011, **12**:124.
33. Ranwez V, Harispe S, Delsuc F, Douzery EJ: **MACSE: Multiple Alignment of Coding SEquences accounting for frameshifts and stop codons**. *PLoS One* 2011, **6**(9):e22594.
34. Wang D, Zhang Y, Zhang Z, Zhu J, Yu J: **KaKs_Calculator 2.0: a toolkit incorporating gamma-series methods and sliding window strategies**. *Genomics Proteomics Bioinformatics* 2010, **8**(1):77-80.
35. Nam K, Mugal C, Nabholz B, Schielzeth H, Wolf JBW, Backström N, Künstner A, Balakrishnan CN, Heger A, Ponting CP *et al*: **Molecular evolution of genes in avian genomes**. *Genome Biology* 2010, **11**(6):R68-R68.
36. Paulo OS, Dias C, Bruford MW, Jordan WC, Nichols RA: **The persistence of Pliocene populations through the Pleistocene climatic cycles: evidence from the phylogeography of an Iberian lizard**. *Proceedings of the Royal Society B: Biological Sciences* 2001, **268**(1476):1625-1630.
37. Rovatsos M, Vukic J, Altmanova M, Johnson Pokorna M, Moravec J, Kratochvil L: **Conservation of sex chromosomes in lacertid lizards**. *Mol Ecol* 2016, **25**(13):3120-3126.
38. Rovatsos M, Vukic J, Kratochvil L: **Mammalian X homolog acts as sex chromosome in lacertid lizards**. *Heredity (Edinb)* 2016, **117**(1):8-13.
39. Kent WJ, Baertsch R, Hinrichs A, Miller W, Haussler D: **Evolution's cauldron: duplication, deletion, and rearrangement in the mouse and human genomes**. *Proc Natl Acad Sci U S A* 2003, **100**(20):11484-11489.
40. Blanchette M, Kent WJ, Riemer C, Elnitski L, Smit AF, Roskin KM, Baertsch R, Rosenbloom K, Clawson H, Green ED *et al*: **Aligning multiple genomic sequences with the threaded blockset aligner**. *Genome Res* 2004, **14**(4):708-715.
41. Pollard KS, Hubisz MJ, Rosenbloom KR, Siepel A: **Detection of nonneutral substitution rates on mammalian phylogenies**. *Genome Res* 2010, **20**(1):110-121.
42. Hubisz MJ, Pollard KS, Siepel A: **PHAST and RPHAST: phylogenetic analysis with space/time models**. *Brief Bioinform* 2011, **12**(1):41-51.
43. Siepel A, Pollard KS, Haussler D: **New Methods for Detecting Lineage-Specific Selection**. In: *Research in Computational Molecular Biology: 10th Annual International Conference,*

RECOMB 2006, Venice, Italy, April 2-5, 2006 Proceedings. Edited by Apostolico A, Guerra C, Istrail S, Pevzner PA, Waterman M. Berlin, Heidelberg: Springer Berlin Heidelberg; 2006: 190-205.
44. Siepel A, Bejerano G, Pedersen JS, Hinrichs AS, Hou M, Rosenbloom K, Clawson H, Spieth J, Hillier LW, Richards S *et al*: **Evolutionarily conserved elements in vertebrate, insect, worm, and yeast genomes**. *Genome Res* 2005, **15**(8):1034-1050.
45. Edgar RC: **MUSCLE: multiple sequence alignment with high accuracy and high throughput**. *Nucleic Acids Research* 2004, **32**(5):1792-1797.
46. Di-Poi N, Montoya-Burgos JI, Duboule D: **Atypical relaxation of structural constraints in Hox gene clusters of the green anole lizard**. *Genome Res* 2009, **19**(4):602-610.
47. de Lanuza GPi, Font E: **Ultraviolet vision in lacertid lizards: evidence from retinal structure, eye transmittance, SWS1 visual pigment genes and behaviour**. *The Journal of Experimental Biology* 2014, **217**(16):2899.
48. van Hazel I, Sabouhanian A, Day L, Endler JA, Chang BSW: **Functional characterization of spectral tuning mechanisms in the great bowerbird short-wavelength sensitive visual pigment (SWS1), and the origins of UV/violet vision in passerines and parrots**. *BMC Evolutionary Biology* 2013, **13**:250-250.
49. Calhoun P: **Exact: Unconditional Exact Test**. In., 1.7 edn; 2016.
50. Bermudez-Santana C, Attolini CS-O, Kirsten T, Engelhardt J, Prohaska SJ, Steigele S, Stadler PF: **Genomic organization of eukaryotic tRNAs**. *BMC Genomics* 2010, **11**(1):270.
51. Scott MS, Ono M: **From snoRNA to miRNA: Dual function regulatory non-coding RNAs**. *Biochimie* 2011, **93**(11):1987-1992.
52. Lohse K, Chmelik M, Martin SH, Barton NH: **Efficient Strategies for Calculating Blockwise Likelihoods Under the Coalescent**. *Genetics* 2016, **202**(2):775-786.
53. Broderick R, Nieminuszczy J, Baddock HT, Deshpande RA, Gileadi O, Paull TT, McHugh PJ, Niedzwiedz W: **EXD2 promotes homologous recombination by facilitating DNA end resection**. *Nature Cell Biology* 2016, **18**:271.
54. Bekker-Jensen S, Danielsen JR, Fugger K, Gromova I, Nerstedt A, Lukas C, Bartek J, Lukas J, Mailand N: **HERC2 coordinates ubiquitin-dependent assembly of DNA repair factors on damaged chromosomes**. *Nature Cell Biology* 2009, **12**:80.
55. Tian C, Xing G, Xie P, Lu K, Nie J, Wang J, Li L, Gao M, Zhang L, He F: **KRAB-type zinc-finger protein Apak specifically regulates p53-dependent apoptosis**. *Nat Cell Biol* 2009, **11**(5):580-591.
56. Trifonov S, Houtani T, Shimizu J-i, Hamada S, Kase M, Maruyama M, Sugimoto T: **GPR155: Gene organization, multiple mRNA splice variants and expression in mouse central nervous system**. *Biochemical and Biophysical Research Communications* 2010, **398**(1):19-25.
57. Nishimura Y, Martin CL, Vazquez-Lopez A, Spence SJ, Alvarez-Retuerto AI, Sigman M, Steindler C, Pellegrini S, Schanen NC, Warren ST *et al*: **Genome-wide expression profiling of lymphoblastoid cell lines distinguishes different forms of autism and reveals shared pathways**. *Human Molecular Genetics* 2007, **16**(14):1682-1698.
58. Laisk-Podar T, Kaart T, Peters M, Salumets A: **Genetic variants associated with female reproductive ageing – potential markers for assessing ovarian function and ovarian stimulation outcome**. *Reproductive BioMedicine Online* 2015, **31**(2):199-209.
59. Linder B, Plöttner O, Kroiss M, Hartmann E, Laggerbauer B, Meister G, Keidel E, Fischer U: **Tdrd3 is a novel stress granule-associated protein interacting with the Fragile-X syndrome protein FMRP**. *Human Molecular Genetics* 2008, **17**(20):3236-3246.
60. Scheffler TL, Park S, Roach PJ, Gerrard DE: **Gain of function AMP-activated protein kinase γ3 mutation (AMPK γ3(R200Q)) in pig muscle increases glycogen storage regardless of AMPK activation**. *Physiological Reports* 2016, **4**(11):e12802.
