# Supplementary material for: Divergent evolution in the genomes of closely related lacertids, Lacerta viridis and L. bilineata, and implications for speciation
Source: Gigascience. 2018 Dec 10;8(2):giy160. doi: 10.1093/gigascience/giy160 (PMC6381762; doi:10.1093/gigascience/giy160)
Supplement: Response_to_Reviewer_Comments_Original_Submission.pdf [file giy160_response_to_reviewer_comments_original_submission.pdf]

Author's Response To Reviewer Comments

Close

Dear Dr. Scott Edmunds, Executive Editor of GigaScience:

We would like to resubmit the revised manuscript entitled “Divergent evolution in the genomes of closely-related lacertids, Lacerta viridis and L. bilineata and implications for speciation”, previously submitted to GigaScience (GIGA-D-18-00173). We believe we have addressed all the reviewers’ helpful and constructive points, performed the necessary modifications and corrections with additional analysis. We would like to thank the reviewers for their time invested in reviewing our manuscript. Below, we present detailed responses to all comments, hoping that the current version meets the quality standards for being published in GigaScience.

Sincerely,
Sree Rohit Raj Kolora, on behalf of all authors

Reviewer suggestions:

Reviewer #1: Kolora and colleagues assemble the genomes of two sister species of green lizards to determine the basis of the genomic incompatibility between them. The study is quite explorative and descriptive, testing a whole bunch of stuff that turned out to be of interest in other taxa. The MS is generally well-written but there are some textual issues listed below.
In general I find this study lacking a clear focus. In lines 116-120 a clear aim of this study is missing. You do a whole bunch of analyses and the MS is quite long-winded, but the common thread is missing or at least obscured. The elements are all there though, so I would like to ask you to make this a bit tighter and to the point. The green lizard hybrid zone is a great system so leverage that in your advantage.
To address this we have rewritten parts of our introduction and discussion to clarify the main aim of the study which is: “identifying candidates and traits involved in divergent evolution of sister species” – namely UV-reflective differences influencing sexual selection, structural divergence in ncRNAs, association of inversions with PSGs, adaptive differences in transcription factors and genes related to brain development and behavior.
The green lizard hybrid zone though interesting is hard to define due to the presence of multiple contact zones (Marzahn et. al. 2016). The demography of the hybrid zones is still not completely clear and this can only be resolved by sequencing the individuals from this region, for which our study provides the reference genomes to compare.

Lines 55-61: I find this section confusing. Your point ii simply seems a restatement of what you want to argue. From point iii it does not follow why this should prevent species merger, because could this not actually drive interspecific introgression?
This part has been corrected to remove portions that have not been described in the manuscript. The basic notion was to explain how inversions can contribute to divergence between species despite ongoing gene flow or admixture. We tried to clarify the third point to avoid confusion.

Lines 71-76: For example, this is all very general and what is lacking is a clear reasoning why you are doing this analysis for the green lizards in particular.
Our apologies for the confusion, this and the previous paragraph are intended to describe the current methods that we can employ to get information from the genomes of two closely related species about their divergence process, including their demographic history (in this particular paragraph). The system of green lizards is introduced in the following paragraph and their features of interest are described.

Line 338-344: Your argument here is not clear (to me). The Ne of Lbil is larger than Lvir because of more structure in Lbil but then you appear to be talking about a subpopulation of Lvir (eastern clade) so that suggests Lvir has plenty of substructure?
This objection is correct and we performed an extensive analysis with more blocks data. This supports Lvir to have a higher Ne compared to Lbil with gene flow from Lbil to Lvir. This part has been rewritten with this additional analysis which includes the model for admixture.

Textual:
Line 46: The "non-essential" is a bit misleading of course, it depends on what perspective (something similar in abstract too).
This has been removed.

Line 105: The "Furthermore" does not follow, it is a disadvantage of the mtDNA/nuDNA sequence study over allozymes that you highlight here. Your aim is to show that there are still uncertainties.
This has been modified accordingly.

Line 109 and 111: Two times "This" is confusing. I don't see how it follows here that genomic re-arrangements are likely.
“This” has been removed and the sentences rewritten, genomic rearrangements were hypothesized earlier between lacertids due to changes in chromosome morphology (citation added).

Line 333-334: "which are 95% identical", these words are unnecessary and make for a confusing sentence (identical to what exactly?).
The sentence has been corrected.

Line 362: word "is" missing
This has been corrected.

Line 398: word "with" redundant
This has been corrected.

Reviewer #2: In the manuscript "Divergent evolution in the genomes of closely-related lacertids, Lacerta viridis and L. bilineata and implications for speciation" Kolora and colleagues present de novo genome assemblies of two species of green lizards based on a combination of Illumina short reads and PacBio sequencing. This resulted in a pair of impressively contiguous and complete genomes which they annotate using a set of tissue-specific transcriptome sequencing. The authors go on to perform an exhaustive array of

analyses to characterize structural, copy number, and sequence divergence between these two species, the ancestral lineage of these two species, and a selection of additional existing genomes. While I feel this work has the potential to be a great contribution, there are number of issues (detailed below) that I feel must first be addressed.

Line 18: "…identify intrinsic and extrinsic factors involved in reproductive isolation" While the divergence between these species is well characterized in this manuscript, the specific factors involved in reproductive isolation are not identified. The authors do suggest a number of viable hypotheses regarding the basis of isolation. Indeed, the authors discuss in their introduction how speciation genes have largely been identified in model organisms due to the tractability of genetic crosses. These speciation genes are specifically those show to play a direct role in reproductive isolation and not all genes that have experienced divergence or selection between lineages. I would consider revising this sentence and elsewhere to reflect this distinction.
The factors for reproductive isolation have been mentioned in lines 303, 370, 383, 397-398. Additional corrections were made in parts of discussion and in the conclusion to include these factors.

Line 25: "…have been critical for reduced reproductive success". Same comment as above. Considering adding "may" to the start of this phrase.
This has been corrected.

Line 49: "diversification" Perhaps the authors mean divergence or speciation?
“diversification” replaced by “divergence eventually leading to speciation”

Line 52: "allowing to test" This sentence is missing a subject.
Subject “us” has been added.

Line 53: A comma is needed after "Specifically".
This has been corrected.

Line 65: First mention of KZNFs here without clear explanation of why they are singled out.
KZNFs have been removed from the sentence. The rationale was that KZNFs are known to be involved in species differences between primates, this has been referred to in the discussion.

Line 98: "Corresponding to lineage B" please add detail here on the citation of lineage B and V.
The detailed citations have been added.

Results: Please report the total size of each assembly in the text.
This information has been added.

Line 129: The Anolis carolinensis genome was not generated via high-coverage Illumina sequencing.
This has been corrected.

Line 129 (and Table S1): Also, while it is accurate that contig N50 in the current study exceed contig N50 for Anolis carolinensis, the scaffold N50 for the carolinensis genome is

multiple orders of magnitude more contiguous (151 Mb - note this has been often misreported at 4Mb in the literature, see Tollis et al 2018 for correct scaffold N50). I suggest making this point clear.
This has been corrected in the supplement. An additional sentence on higher N50 of anolis genome was added.

Line 147: The reported karyotype is that of Anolis carolinensis, but is not shared by all anoles. Perhaps rephrase?
A. carolinensis has been specifically mentioned.

Line 151: Please provide a citation for a divergence time of 150Mya or more.
Citation has been provided.

Line 186: This estimate of Fst should be considered a maximum as using heterozygosity from single individuals will have the effect of maximizing the estimate of Dxy and minimizes estimates of pi. I suggest making it clear that actual Fst is likely lower.
The heterozygosity of an individual is an unbiased estimate of pairwise pi (given a panmictic population) and d_xy is similarly sample size independent. Hence we would like to kindly inform that our estimate of F_st is indeed unbiased and not some upper lower bound.

Line 187, 203, 489: While I commend the authors for including their Mathematica code as a supplement, I think addition detail on this method and results should be provided. What are the likelihoods of each model? How much better than other models was model M4.1? How were more complex models compared to models with fewer parameters?
This was previously present in the supplement. We have moved it to the main text including additional analysis with an admixture model.

Line 254: The maintenance of opsin paralogs over long periods of evolutionary times certainly suggest they are functionally important, but the phrase "similar diurnal adaptations" suggests parallel evolution, where the more likely scenario (I think) is simply conservation.
We completely agree and did not intend to imply parallel evolution. We have corrected this sentence to imply conservation.

Line 305: "Neutrally evolving genes" Please be specific about how genes are classified as "neutrally evolving". Does this mean they are unconstrained or that there is no evidence of positive selection in branch-site tests?
We have corrected this to imply genes with no signs of positive selection.

Line 311: "indels over-showing" I'm not clear what is meant by "over-showing". Please consider rephrasing?
This has been corrected with “obscuring”.

Line 316: How were multiple Boschloo exact-tests tests corrected?
FDRs were calculated and the values have been added.

Line 337: "Therefore, our results support the separate species status of L. viridis and L. bilineata." Which species concept is used to underpin this statement?
We rewrote this statement more carefully, since we do not want to indulge in any discussion

of the numerous and controversial species concepts here.

Line 338: "Confirmed" The demographic modeling supported unidirectional gene flow, but confirmed seems too strong of a sentiment here.
Agreed, this has been replaced with “supports”.

Line 340: While population subdivision can increase heterozygosity and estimates of effective population size via the Wahlund effect, the heterozygosity of a single individual (a member of just a single subpopulation) would not reflect this increase heterozygosity. I would suggest the authors consider other possible explanations. For example, their hypothesis of unidirectional gene flow into L. bilineata could, by itself, explain elevated heterozygosity if the sequenced individual contains L. viridis alleles due to introgression.
We have re-analysed the demographic patterns with additional data and more extensive models including simple divergence, isolation with migration and admixture. In this improved analyses, the best model supports gene flow predominantly from L. bilineata to L. viridis. Since gene flow occurs in this direction, L. viridis could have a higher effective population size.

Line 508: "on overage" Typo?
This has been corrected.

Line 509: "… this data" should be "… these data"
This has been corrected.

Line 640: Please cite any relevant animal use and care authorizations.
The capture permits were added in the acknowledgements, and the animal care for experimentation has been specified in the methods. The permits can be provided to the editor upon request. We do not have a specific ethics committee at our institution, hence we followed the guidelines provided by the Herpetological Animal Care and Use Committee (HACC) of the American Society of Ichthyologists and Herpetologists.

Close
