# Supplementary material for: Divergent evolution in the genomes of closely related lacertids, Lacerta viridis and L. bilineata, and implications for speciation
Source: Gigascience. 2018 Dec 10;8(2):giy160. doi: 10.1093/gigascience/giy160 (PMC6381762; doi:10.1093/gigascience/giy160)
Supplement: Response_to_Reviewer_Comments_Revision_1.pdf [file giy160_response_to_reviewer_comments_revision_1.pdf]

Author's Response To Reviewer Comments

Close

Dear Dr. Scott Edmunds, Executive Editor of GigaScience:

We would like to resubmit the revised manuscript entitled “Divergent evolution in the genomes of closely-related lacertids, Lacerta viridis and L. bilineata and implications for speciation”, previously submitted to GigaScience (GIGA-D-18-00173). We believe that all the comments and suggestions have been addressed and the current version meets the high quality of research published in GigaScience.
Sincerely,
Sree Rohit Raj Kolora, on behalf of all authors

Line 140: "since" seems out of place in the sentence
This has been corrected

Line 221: "our estimate of the split between L. viridis and L. bilineata corresponds to 1.15 Mya and 20.37 Mya respectively (Table S8)." Add comma before respectively
The comma has been added

Line 413: "Animals were captures" should be "Animals were captured"
This has been corrected

A. Please change the Data Access section to an Availability of Supporting Data Section, and in this please cite the GigaDB and Zenodo DOIs in the references.
The section has been modified and the databases have been cited

B. Please add relevant identifiers into the paper such as NCBI taxon IDs, ORCID IDs for the authors and RRIDs for software tools.
The relevant IDs have been added to the best of our knowledge

C. Please add an Abbreviations section at the end of the paper to include relevant abbreviations.
A section of abbreviations has been added

Close
