# Supplementary material for: Divergent evolution in the genomes of closely related lacertids, Lacerta viridis and L. bilineata, and implications for speciation
Source: Gigascience. 2018 Dec 10;8(2):giy160. doi: 10.1093/gigascience/giy160 (PMC6381762; doi:10.1093/gigascience/giy160)
Supplement: Reviewer_1_Report_(Original_Submission) -- Ben Wielstra — 6/20/2018 Reviewed [file giy160_reviewer_1_report_(original_submission).pdf]

Reviewer Report

Title: **Divergent evolution in the genomes of closely-related lacertids, Lacerta viridis and L. bilineata and implications for speciation**

Version: **Original Submission** **Date:** 6/20/2018

Reviewer name: **Ben Wielstra**

Reviewer Comments to Author:

Kolora and colleagues assemble the genomes of two sister species of green lizards to determine the basis of the genomic incompatibility between them. The study is quite explorative and descriptive, testing a whole bunch of stuff that turned out to be of interest in other taxa. The MS is generally well-written but there are some textual issues listed below.

In general I find this study lacking a clear focus. In lines 116-120 a clear aim of this study is missing. You do a whole bunch of analyses and the MS is quite long-winded, but the common thread is missing or at least obscured. The elements are all there though, so I would like to ask you to make this a bit tighter and to the point. The green lizard hybrid zone is a great system so leverage that in your advantage.

Lines 55-61: I find this section confusing. Your point ii simply seems a restatement of what you want to argue. From point iii it does not follow why this should prevent species merger, because could this not actually drive interspecific introgression?

Lines 71-76: For example, this is all very general and what is lacking is a clear reasoning why you are doing this analysis for the green lizards in particular.

Line 338-344: Your argument here is not clear (to me). The Ne of Lbil is larger than Lvir because of more structure in Lbil but then you appear to be talking about a subpopulation of Lvir (eastern clade) so that suggests Lvir has plenty of substructure?

Textual:

Line 46: The "non-essential" is a bit misleading of course, it depends on what perspective (something similar in abstract too).

Line 105: The "Furthermore" does not follow, it is a disadvantage of the mtDNA/nuDNA sequence study over allozymes that you highlight here. Your aim is to show that there are still uncertainties.

Line 109 and 111: Two times "This" is confusing. I don't see how it follows here that genomic re-arrangements are likely.

Line 333-334: "which are 95% identical", these words are unnecessary and make for a confusing sentence (identical to what exactly?).

Line 362: word "is" missing

Line 398: word "with" redundant

Methods

Are the methods appropriate to the aims of the study, are they well described, and are necessary controls included? Choose an item.

Conclusions

Are the conclusions adequately supported by the data shown? Choose an item.

Reporting Standards

Does the manuscript adhere to the journal's guidelines on minimum standards of reporting? Choose an item.

Choose an item.

Statistics

Are you able to assess all statistics in the manuscript, including the appropriateness of statistical tests used? Choose an item.

Quality of Written English

Please indicate the quality of language in the manuscript: Choose an item.

Declaration of Competing Interests

Please complete a declaration of competing interests, considering the following questions:

- Have you in the past five years received reimbursements, fees, funding, or salary from an organisation that may in any way gain or lose financially from the publication of this manuscript, either now or in the future?
- Do you hold any stocks or shares in an organisation that may in any way gain or lose financially from the publication of this manuscript, either now or in the future?
- Do you hold or are you currently applying for any patents relating to the content of the manuscript?
- Have you received reimbursements, fees, funding, or salary from an organization that holds or has applied for patents relating to the content of the manuscript?
- Do you have any other financial competing interests?
- Do you have any non-financial competing interests in relation to this paper?

If you can answer no to all of the above, write 'I declare that I have no competing interests' below. If your reply is yes to any, please give details below.

None

I agree to the open peer review policy of the journal. I understand that my name will be included on my report to the authors and, if the manuscript is accepted for publication, my named report including any attachments I upload will be posted on the website along with the authors' responses. I agree for my

report to be made available under an Open Access Creative Commons CC-BY license (http://creativecommons.org/licenses/by/4.0/). I understand that any comments which I do not wish to be included in my named report can be included as confidential comments to the editors, which will not be published.

I agree to the open peer review policy of the journal

To further support our reviewers, we have joined with Publons, where you can gain additional credit to further highlight your hard work (see: https://publons.com/journal/530/gigascience). On publication of this paper, your review will be automatically added to Publons, you can then choose whether or not to claim your Publons credit. I understand this statement.

Yes Choose an item.
