# Supplementary material for: Divergent evolution in the genomes of closely related lacertids, Lacerta viridis and L. bilineata, and implications for speciation
Source: Gigascience. 2018 Dec 10;8(2):giy160. doi: 10.1093/gigascience/giy160 (PMC6381762; doi:10.1093/gigascience/giy160)
Supplement: Reviewer_2_Report_(Original_Submission) -- Anthony Geneva — 6/22/2018 Reviewed [file giy160_reviewer_2_report_(original_submission).pdf]

Reviewer Report

Title: **Divergent evolution in the genomes of closely-related lacertids, Lacerta viridis and L. bilineata and implications for speciation**

Version: **Original Submission** **Date:** 6/22/2018

Reviewer name: **Anthony Geneva**

Reviewer Comments to Author:

In the manuscript "Divergent evolution in the genomes of closely-related lacertids, Lacerta viridis and L. bilineata and implications for speciation" Kolora and colleagues present de novo genome assemblies of two species of green lizards based on a combination of Illumina short reads and PacBio sequencing. This resulted in a pair of impressively contiguous and complete genomes which they annotate using a set of tissue-specific transcriptome sequencing. The authors go on to perform an exhaustive array of analyses to characterize structural, copy number, and sequence divergence between these two species, the ancestral lineage of these two species, and a selection of additional existing genomes. While I feel this work has the potential to be a great contribution, there are number of issues (detailed below) that I feel must first be addressed.

Line 18: "…identify intrinsic and extrinsic factors involved in reproductive isolation" While the divergence between these species is well characterized in this manuscript, the specific factors involved in reproductive isolation are not identified. The authors do suggest a number of viable hypotheses regarding the basis of isolation. Indeed, the authors discuss in their introduction how speciation genes have largely been identified in model organisms due to the tractability of genetic crosses. These speciation genes are specifically those show to play a direct role in reproductive isolation and not all genes that have experienced divergence or selection between lineages. I would consider revising this sentence and elsewhere to reflect this distinction.

Line 25: "…have been critical for reduced reproductive success". Same comment as above. Considering adding "may" to the start of this phrase.

Line 49: "diversification" Perhaps the authors mean divergence or speciation?

Line 52: "allowing to test" This sentence is missing a subject.

Line 53: A comma is needed after "Specifically".

Line 65: First mention of KZNFs here without clear explanation of why they are singled out.

Line 98: "Corresponding to lineage B" please add detail here on the citation of lineage B and V.

Results: Please report the total size of each assembly in the text.

Line 129: The Anolis carolinensis genome was not generated via high-coverage Illumina sequencing.

Line 129 (and Table S1): Also, while it is accurate that contig N50 in the current study exceed contig N50 for Anolis carolinensis, the scaffold N50 for the carolinensis genome is multiple orders of magnitude more contiguous (151 Mb - note this has been often misreported at 4Mb in the literature, see Tollis et al 2018 for correct scaffold N50). I suggest making this point clear.

Line 147: The reported karyotype is that of Anolis carolinensis, but is not shared by all anoles. Perhaps rephrase?

Line 151: Please provide a citation for a divergence time of 150Mya or more.

Line 186: This estimate of Fst should be considered a maximum as using heterozygosity from single individuals will have the effect of maximizing the estimate of Dxy and minimizes estimates of pi. I suggest making it clear that actual Fst is likely lower.

Line 187, 203, 489: While I commend the authors for including their Mathematica code as a supplement, I think addition detail on this method and results should be provided. What are the likelihoods of each model? How much better than other models was model M4.1? How were more complex models compared to models with fewer parameters?

Line 254: The maintenance of opsin paralogs over long periods of evolutionary times certainly suggest they are functionally important, but the phrase "similar diurnal adaptations" suggests parallel evolution, where the more likely scenario (I think) is simply conservation.

Line 305: "Neutrally evolving genes" Please be specific about how genes are classified as "neutrally evolving". Does this mean they are unconstrained or that there is no evidence of positive selection in branch-site tests?

Line 311: "indels over-showing" I'm not clear what is meant by "over-showing". Please consider rephrasing?

Line 316: How were multiple Boschloo exact-tests tests corrected?

Line 337: "Therefore, our results support the separate species status of L. viridis and L. bilineata." Which species concept is used to underpin this statement?

Line 338: "Confirmed" The demographic modeling supported unidirectional gene flow, but confirmed seems too strong of a sentiment here.

Line 340: While population subdivision can increase heterozygosity and estimates of effective population size via the Wahlund effect, the heterozygosity of a single individual (a member of just a single subpopulation) would not reflect this increase heterozygosity. I would suggest the authors consider other possible explanations. For example, their hypothesis of unidirectional gene flow into L. bilineata could, by itself, explain elevated heterozygosity if the sequenced individual contains L. viridis alleles due to introgression.

Line 508: "on overage" Typo?

Line 509: "… this data" should be "… these data"

Line 640: Please cite any relevant animal use and care authorizations.

Methods

Are the methods appropriate to the aims of the study, are they well described, and are necessary controls included? Choose an item.

Conclusions

Are the conclusions adequately supported by the data shown? Choose an item.

Reporting Standards

Does the manuscript adhere to the journal's guidelines on minimum standards of reporting? Choose an item.

Choose an item.

Statistics

Are you able to assess all statistics in the manuscript, including the appropriateness of statistical tests used? Choose an item.

Quality of Written English

Please indicate the quality of language in the manuscript: Choose an item.

Declaration of Competing Interests

Please complete a declaration of competing interests, considering the following questions:

- Have you in the past five years received reimbursements, fees, funding, or salary from an organisation that may in any way gain or lose financially from the publication of this manuscript, either now or in the future?
- Do you hold any stocks or shares in an organisation that may in any way gain or lose financially from the publication of this manuscript, either now or in the future?
- Do you hold or are you currently applying for any patents relating to the content of the manuscript?
- Have you received reimbursements, fees, funding, or salary from an organization that holds or has applied for patents relating to the content of the manuscript?
- Do you have any other financial competing interests?
- Do you have any non-financial competing interests in relation to this paper?

If you can answer no to all of the above, write 'I declare that I have no competing interests' below. If your reply is yes to any, please give details below.

I declare that I have no competing interests.

I agree to the open peer review policy of the journal. I understand that my name will be included on my report to the authors and, if the manuscript is accepted for publication, my named report including any attachments I upload will be posted on the website along with the authors' responses. I agree for my report to be made available under an Open Access Creative Commons CC-BY license (http://creativecommons.org/licenses/by/4.0/). I understand that any comments which I do not wish to be included in my named report can be included as confidential comments to the editors, which will not be published.

I agree to the open peer review policy of the journal

To further support our reviewers, we have joined with Publons, where you can gain additional credit to further highlight your hard work (see: https://publons.com/journal/530/gigascience). On publication of this paper, your review will be automatically added to Publons, you can then choose whether or not to claim your Publons credit. I understand this statement.

Yes Choose an item.
